# Direct Production of Bio-Recalcitrant Carboxyl-Rich Alicyclic Molecules Evidenced in a Bacterium-Induced Steroid Degradation Experiment

**DOI:** 10.1128/spectrum.04693-22

**Published:** 2023-02-06

**Authors:** Zijing Liu, Ruanhong Cai, Yi-Lung Chen, Xiaocun Zhuo, Chen He, Qiang Zheng, Ding He, Quan Shi, Nianzhi Jiao

**Affiliations:** a State Key Laboratory of Marine Environmental Science, College of Ocean and Earth Sciences, Fujian Key Laboratory of Marine Carbon Sequestration, Xiamen University, Xiamen, China; b Department of Microbiology, Soochow University, Taipei, Taiwan; c State Key Laboratory of Heavy Oil Processing, China University of Petroleum, Beijing, China; d Department of Ocean Science and the Southern Marine Science and Engineering Guangdong Laboratory, The Hong Kong University of Science and Technology, Hong Kong, China; University of Minnesota, Twin Cities

**Keywords:** steroids, refractory dissolved organic matter, carboxyl-rich alicyclic molecules, carbon sequestration, terrestrial contaminants

## Abstract

Carboxyl-rich alicyclic molecules (CRAM) are highly unsaturated compounds extensively distributed throughout aquatic environments and sediments. This molecular group is widely referred to as a major proxy of recalcitrant organic materials, but its direct biosynthesis remains unclear. Steroids are a typical anthropogenic contaminant and have been previously suggested to be precursors of CRAM; however, experimental evidence to support this hypothesis is lacking. Here, a steroid-degrading bacterium, Comamonas testosteroni ATCC 11996, was incubated in a liquid medium supplemented with testosterone (a typical steroid) as the sole carbon source for 90 days. Testosterone-induced metabolites (TIM) were extracted for molecular characterization and to examine the bioavailability during an additional 90-day incubation after inoculation with a natural coastal microbial assemblage. The results showed that 1,775 molecular formulas (MFs) were assigned to TIM using ultrahigh-resolution mass spectrometry, with 66.99% categorized as CRAM-like constituents. A large fraction of TIM was respired or transformed during the additional 90-day seawater incubation; nevertheless, 638 MFs of the TIM persisted or increased during incubation. Among the 638 MFs, 394 were commonly assigned in natural deep seawater samples (depths of 500 to 2,000 m) from the South China Sea. Compared to the catabolites of the well-established testosterone degradation pathway, we compiled a list of bio-refractory MFs and potential chemical structures, some of which shared structural homology with CRAM. These results demonstrated direct microbial production of bio-refractory CRAM from steroid hormones and indicated that some of the biogenic CRAM resisted microbial decomposition, potentially contributing to the aquatic refractory dissolved organic matter (DOM) pool.

**IMPORTANCE** CRAM are an operationally defined DOM group comprising a complex mixture of carboxylated and fused alicyclic structures. This DOM group is majorly characterized as refractory DOM in the marine environment. However, the origins of the complex CRAM remain unclear. In this study, we demonstrated that testosterone (a typical steroid) could be transformed into bio-refractory CRAM by a single bacterial strain and observed that some of the CRAM highly resisted microbial degradation. Through molecular comparison and screening, potential chemical structures of steroid-induced CRAM were suggested. This study established the biological connection between steroids and bio-refractory CRAM, and it provides a novel perspective explaining the fate of terrestrial contaminants in aquatic environments.

## INTRODUCTION

Natural dissolved organic matter (DOM) is widely distributed in aquatic environments and sediments of lakes, rivers, and oceans and represents a tremendous reservoir of reduced carbon on the Earth (1). Terrestrial DOM contains a fraction of anthropogenic contaminants that exert ecological effects across the aquatic continuum, from rivers to coastal oceans (2, 3). Steroids, as typical organic contaminants in aquatic environments (4), are mainly produced by eukaryotic organisms (e.g., mammals) and are discharged from domestic water, swine farms, and broiler litter (5, 6). Most steroids escape rapid degradation (7, 8) and are recognized as biologically recalcitrant organic matter that is widely distributed in rivers, coastal waters, and sediments (4, 9, 10). An estimate of global steroid disposal was 18,270 tons in 2015, approximately 70% of which was discharged into aquatic environments (11, 12). With their recalcitrant properties in aquatic environments, accumulated steroids can have detrimental ecological effects (13). For example, a typical steroid, testosterone, is derived from widely distributed cholesterol and has intermediate catabolites similar to those of cholesterol (4, 9, 10). Long-term testosterone exposure can affect the physiological activity of aquatic organisms (14, 15).

Steroids are a series of compounds with the structure of a four-membered hydrocarbon core (perhydrocyclopentanophenanthrene) (16). Within this, ring A is a cyclohexane ring on the left; it is attached to another six-membered ring, ring B. Ring C follows ring B, and ring D is a cyclopentane system. Recently, a study proposed that some microorganisms have the function of oxygen insertion and ring opening, allowing the conversion of steroids (with alicyclic structure) to a potentially higher biologically recalcitrant DOM known as carboxyl-rich alicyclic molecules (CRAM) (17, 18). CRAM are currently a widely referred and quickly acquirable refractory DOM (RDOM) proxy that has been analyzed by nuclear magnetic resonance and ultrahigh-resolution mass spectrometry (18–22). Therefore, it is thought that the microbial transformation of steroids into CRAM in natural aquatic environments may potentially mitigate the detrimental environmental effects of steroids, but this requires experimental evidence.

As first coined in the study of Hertkorn et al. in 2006 (23), CRAM are an operationally defined molecular group that is a complex mixture of DOM based on the functional groups in molecules. Increasing studies have demonstrated that CRAM can be produced after successive microbial processing of various organic substrates (18). A recent study revealed the direct production of CRAM by chemoautotrophs (ammonia-oxidizing archaea) and speculated that such CRAM are unlikely bio-recalcitrant accumulation (24). However, whether CRAM can be directly produced by a single heterotroph and whether such CRAM are bio-recalcitrant are still unknown.

To date, none of the CRAM compounds have been assigned to certain chemical structures, although some structural proxies of CRAM have been proposed (23). Specific CRAM compounds with a clear recalcitrant nature would additionally bridge the knowledge gap between the chemical structure of RDOM and its production (18, 24). Recently, potential metabolic pathways have been suggested for producing specific sterol-derived CRAM (such as cholesterol, testosterone, and other steroid hormones) (17, 25), providing a clue toward revealing the specific chemical structures of CRAM. However, experimental evidence is needed to ascertain this hypothesis. In addition, different paths may explain the potential fate of steroids, which can be microbially transformed into CRAM during their transportation from rivers to coastal oceans, thereby potentially favoring carbon sequestration in aquatic systems.

To test the hypotheses discussed above, the typical steroid hormone testosterone was selected as the sole carbon amended in a liquid medium, which was inoculated with a testosterone-degrading bacterium to for a 90-day incubation period. The molecular composition of testosterone-induced metabolites (TIM) was characterized using ultrahigh-resolution Fourier-transform ion cyclotron resonance mass spectrometry (FT-ICR MS). TIM were further amended into a coastal seawater sample containing a microbial assemblage to conduct an additional 90-day incubation assay. The molecular composition of TIM was further monitored and compared with natural deep sea DOM, to examine the bioavailability of TIM and screen for potential proxies of biologically refractory DOM in the ocean. This two-step incubation simulated a potential microbial transformation, as we examined the direct transformation from steroids to CRAM-like molecules by a single strain during their transportation from land to coastal seawater. Together, these experiments should shed light into the potential production of bio-refractory CRAM and provide a novel perspective to explain the fate of terrestrial contaminants in aquatic environments.

## RESULTS

### Molecular composition of TIM.

**FIG 1:**
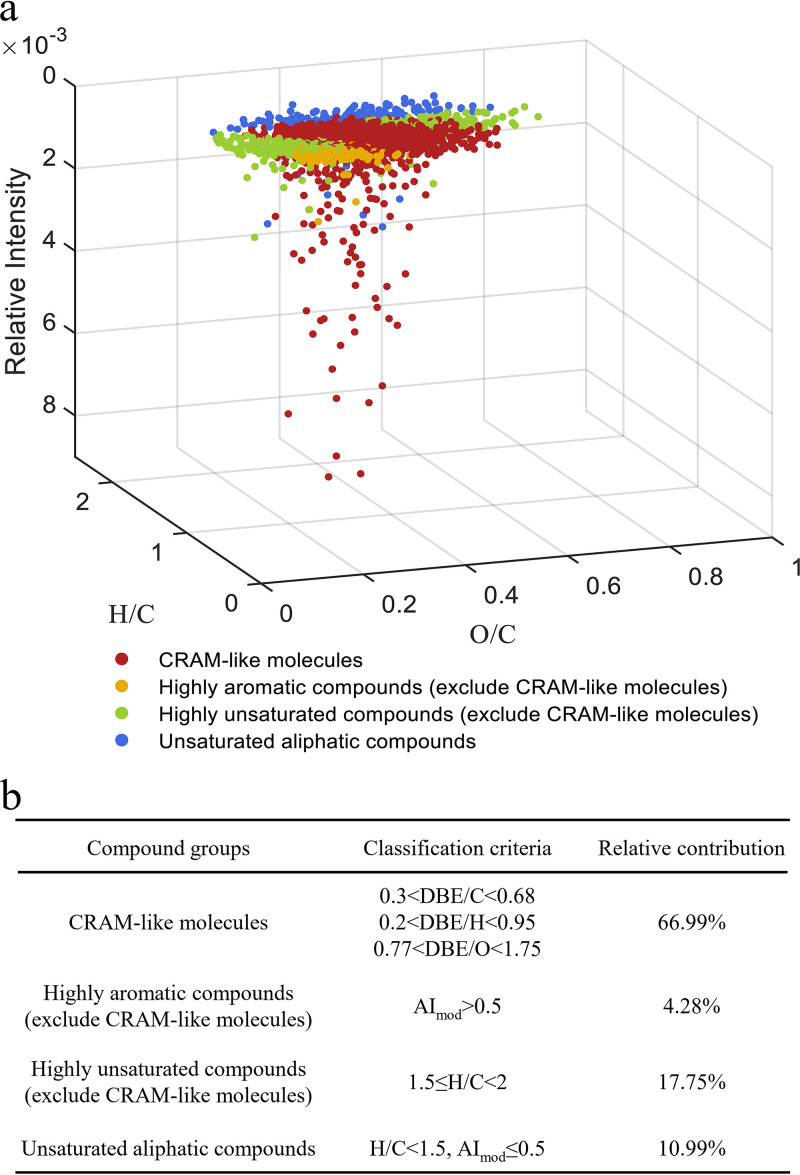
Chemical composition of TIM derived from the 90-day incubation period. (a) O/C, H/C, and the normalized intensity of the DOM molecules. The assigned formulas with different classifications are shown in different colors: CRAM-like molecules, reddish-brown; polycyclic aromatics, yellow; highly aromatic molecules, gray; highly unsaturated molecules, green; unsaturated aliphatic molecules, blue. (b) The ratios of each compound group were calculated by dividing its counts by the total counts of the assigned formulas in the TIM sample.

After the 90-day incubation with Comamonas testosteroni ATCC 11996, a total of 1,775 molecular formulas (MFs) were assigned to the TIM using ultrahigh-resolution FT-ICR MS. The 1,775 MFs were classified into four molecular classes: CHO, CHNO, CHOS, and CHNOS, accounting for 53.07%, 19.21%, 23.10%, and 4.62% of the total formula numbers, respectively (see Fig. S3 in the supplemental material). In addition, four representative compound groups, including CRAM-like, highly aromatic-like, highly unsaturated-like, and unsaturated aliphatic-like molecules (Fig. 1a), and their relative contributions (Fig. 1b) were also calculated. Among these, CRAM-like molecules accounted for 1,189 MFs and nearly 66.99% of the total MFs in the TIM (Fig. 1b). These results indicated that testosterone could be transformed into organic matter with molecular properties similar to those of the CRAM constituents.

### Bioavailability of TIM.

**FIG 2:**
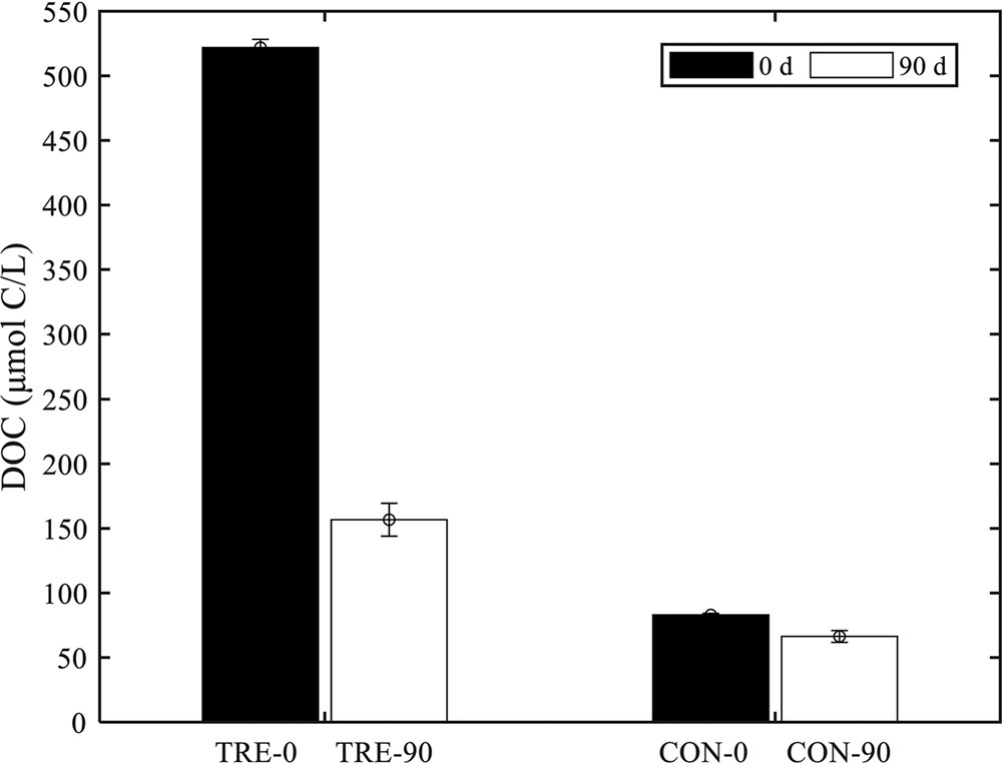
DOC concentrations on days 0 and 90 in the TIM-amended experiment. Water samples were collected from coastal seawater. TRE, TIM-amended treatments; CON, controls.

Dissolved organic carbon (DOC) concentration variation analysis showed that the starting DOC concentration was 83.02 ± 1.20 μmol C/liter in the control incubations and decreased to 66.37 ± 0.06 μmol C/liter at the end of the 90-day incubation (Fig. 2). In the TIM-amended incubations, the initial DOC concentration was 521.86 ± 6.36 μmol C/liter and decreased to 156.72 ± 12.74 μmol C/liter on day 90. Therefore, nearly 79.41% (~348.49 μmol C/liter) of the amended TIM was respired or transformed, indicating that the TIM contained a large fraction of labile or semilabile organic constituents.

### Compositional variations of TIM.

**FIG 3:**
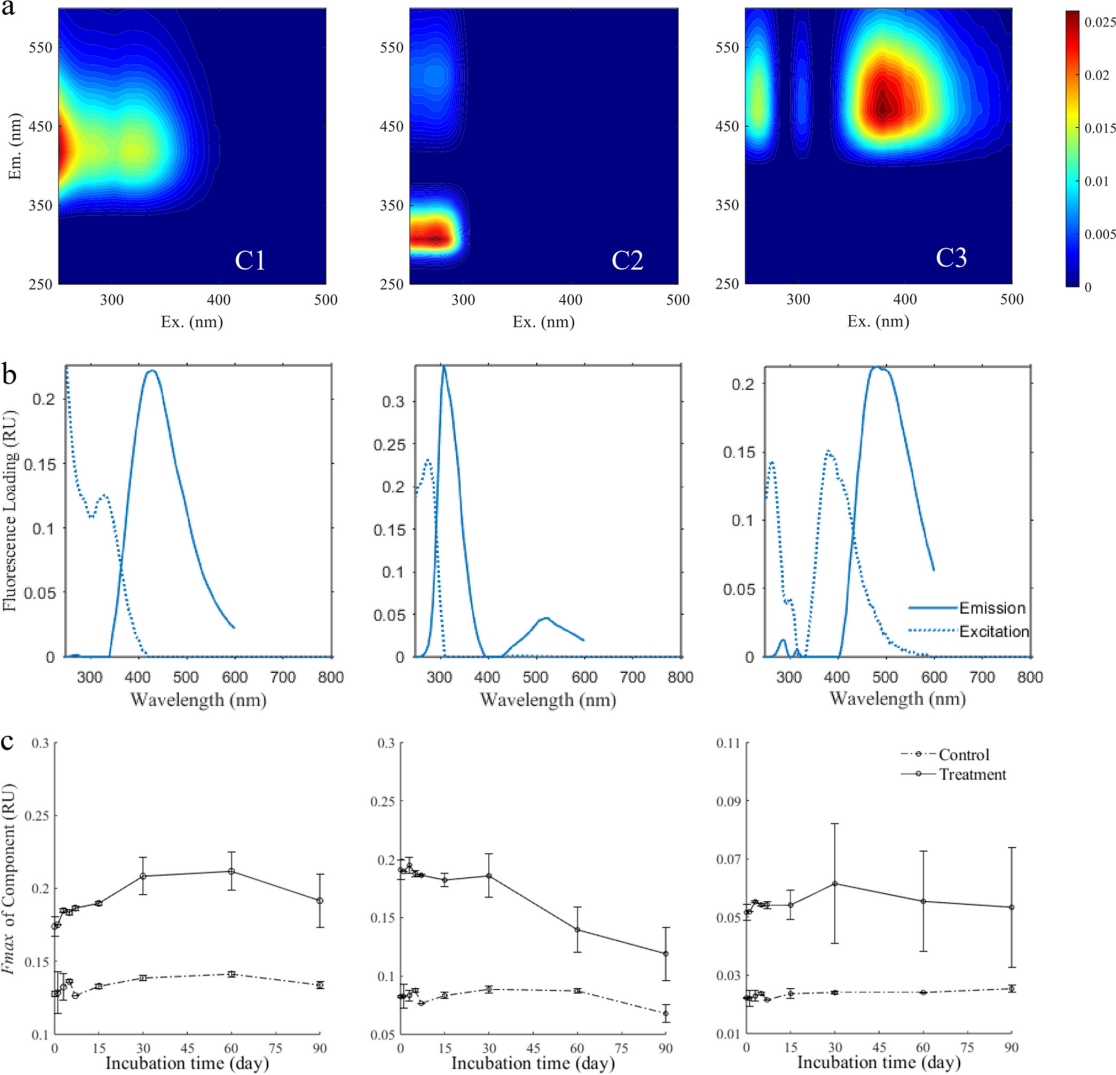
Variation of three fluorescent components analyzed by EEM. (a) Fluorescent components categorized by PARAFAC. (b) Loadings of four fluorescent components. (c) Variation of the four fluorescent components in the control and treatment groups during the 90-day incubation period with seawater.

Excitation-emission matrix (EEM) and FT-ICR MS were applied to reveal variations in the optical and molecular compositions of TIM during the 90-day seawater incubation. EEM fluorescence was applied with parallel factor analysis (PARAFAC) to track the variations in fluorescent DOM (FDOM) components in the control and TIM-amended treatments during the 90-day incubation experiment. Three different FDOM components were characterized (Fig. 3), among which components C1 and C3 exhibited fluorescence properties similar to those of humic-like substances, whereas component C2 was generally identified as tryptophan-like DOM (26, 27). The fluorescence intensity of C1 increased, whereas that of C2 decreased in the TIM-amended incubations, and the fluorescence intensity of C3 was relatively constant; therefore, the TIM contained both humic-like and protein-like components, and the protein-like FDOM could be degraded or transformed, resulting in the accumulation of humic-like components.

The molecular composition variation of TIM was monitored in the controls and TIM-amended treatments over 90 days using ultrahigh-resolution FT‐ICR MS analysis (Fig. S1). Table 1 summarizes the information of the assigned MFs and averages of molecular indices, including hydrogen to carbon (H/C_a_) ratios, oxygen to carbon (O/C_a_) ratios, double bond equivalents (DBE_a_), modified aromaticity indices (AI_moda_), and nominal oxidation state of carbon (NOCS_a_). The O/C_a_, AI_moda_, and DBE_a_ values in the TIM-amended treatments were 0.459 ± 0.011, 0.234 ± 0.002, and 7.961 ± 0.026, respectively. All these indices were relatively low compared with those in the initial controls (0.467 ± 0.019, 0.240 ± 0.002, and 8.291 ± 0.081 for O/C_a_, AI_moda_, and DBE_a_, respectively). The H/C_a_ value in the initial TIM-amended treatments was 1.277 ± 0.004, which was relatively high than that in the initial controls (1.273 ± 0.013). These results indicated that the TIM was less oxygenated and in a relatively highly saturated state. The values of AI_moda_ and DBE_a_ in the 90-day TIM-amended treatments were 0.253 ± 0.001 and 7.973 ± 0.071, respectively, and were slightly increased compared to those in the day 0 treatments (0.234 ± 0.002 for AI_moda_ and 7.961 ± 0.026 for DBE_a_).

**TABLE 1:** Averages of H/C_a_, O/C_a_, AI_moda_, DBE_a_, and NOSC_a_ of each sample in the coastal seawater incubation

Incubation^*a*^	H/c_a_	O/c_a_	AI_moda_	DBE_a_	NOSC_a_
CON 0-day	1.273 ± 0.013	0.467 ± 0.019	0.240 ± 0.002	8.291 ± 0.081	−0.291 ± 0.055
CON 90-day	1.254 ± 0.003	0.480 ± 0.004	0.246 ± 0.001	8.345 ± 0.015	−0.241 ± 0.014
TRE 0-day	1.277 ± 0.004	0.459 ± 0.011	0.234 ± 0.002	7.961 ± 0.026	−0.293 ± 0.028
TRE 90-day	1.261 ± 0.004	0.443 ± 0.003	0.253 ± 0.001	7.973 ± 0.071	−0.301 ± 0.009

a TRE, TIM addition groups; CON, no extra carbon source addition.

The relative contributions of the major formula classes (CHO, CHNO, CHOS, and CHNOS) were calculated and are shown in Fig. S3. On day 0, the respective proportions of CHNO, CHOS, and CHNOS were 37.12%, 20.43%, and 9.31% in the treatments, whereas they were 43.96%, 10.71%, and 3.80% in the controls. Therefore, the addition of TIM contained a relatively high abundance of S-bearing DOM in seawater, especially abundant S-containing molecules. The concentrations of S-containing compounds in the treatment groups were much higher than those in the controls until the end of the incubation period. These experimental results indicated that testosterone degradation may contribute to S-containing DOM in natural water.

### Potential RDOM proxy screened from the TIM.

**FIG 4:**
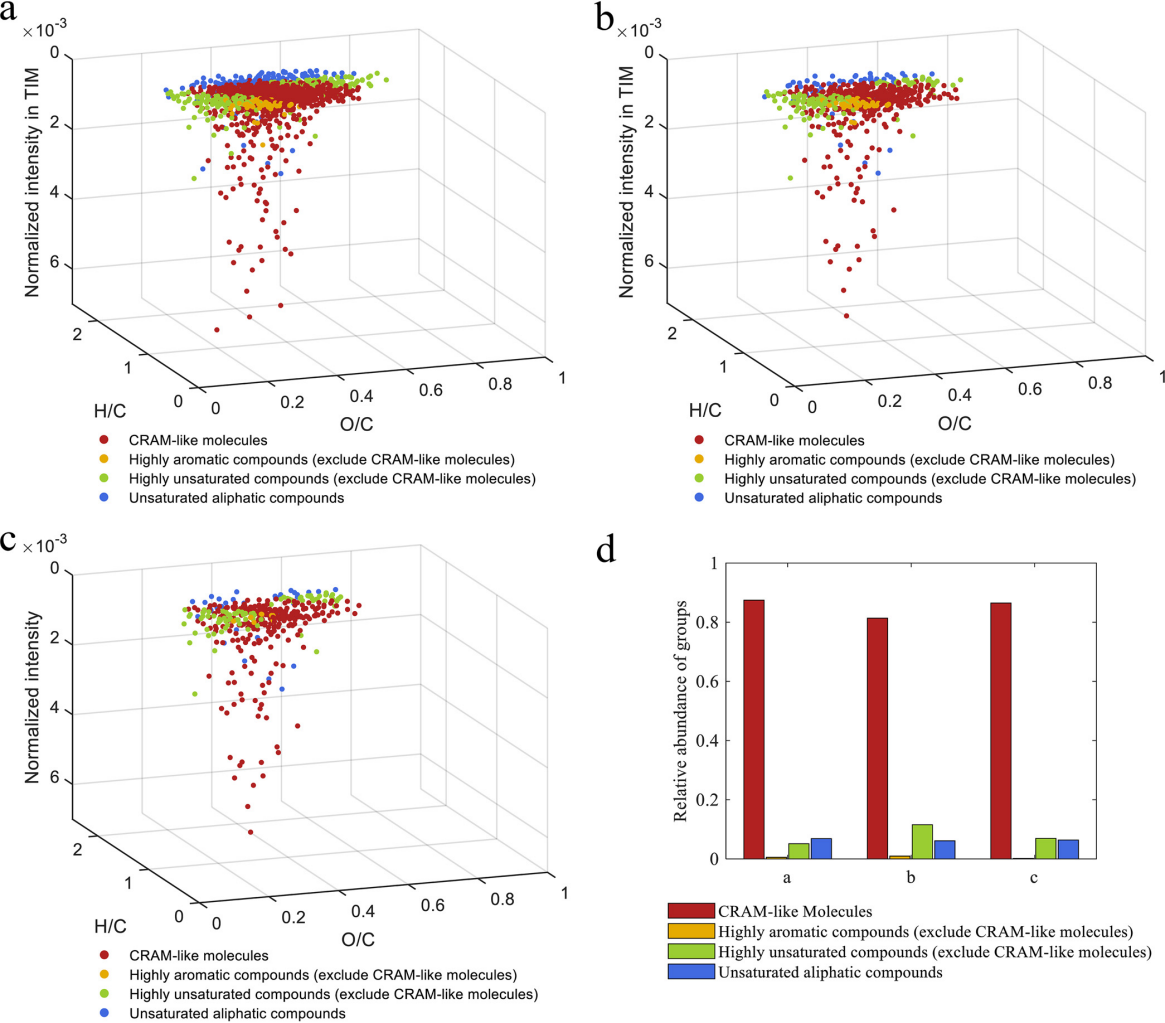
Three-dimensional van Krevelen diagrams showing the molecular H/C, O/C, and normalized intensity in TIM. (a) TIM that persisted during the whole seawater incubation. (b) TIM that accumulated during the seawater incubation. (c) Accumulated TIM that were widely distributed at four depths within deep seawater. (d) Relative abundances of molecular compound groups. Each compound group was calculated by dividing its formula intensity by the total intensity of the assigned formulas in the testosterone metabolite sample. The diagram shows the O/C, H/C, and normalized intensity of the DOM molecules. The assigned formulas with different classifications are shown in different colors: CRAM-like molecules, reddish-brown; highly aromatic compounds excluding CRAM-like molecules, yellow; highly unsaturated compounds excluding CRAM-like molecules, green; unsaturated aliphatic compounds, blue.

Although the TIM contained a large fraction of bio-labile DOM, some molecules within the 1,775 TIM persisted during the 90-day incubation. We further screened these molecules as potential proxies of biologically recalcitrant DOM. As shown below in “Fate of steroids in aquatic environments,” a total of 1,775 MFs were assigned from the TIM, among which 1,290 MFs (72.67% of the 1,775 MFs) consistently existed or were increased at a normalized intensity during the 90-day seawater incubation (Table 2; Table S1); these 1,290 MFs were defined as persistent molecules. Among the 1,290 MFs, 638 MFs (49.46% of the 1,290 MFs) were further screened because of their increased normalized intensities after the 90-day incubation (Table 2; Table S2). These 638 MFs were then defined as accumulated molecules and were further compared with DOM obtained from four depths of deep seawater samples (500, 800, 1,000, and 2,000 m) from the station South East Asia Time Series Study (SEATS) of the South China Sea (22). A total of 394 MFs (61.75% of the 638 accumulated molecules) were commonly found in the four deep seawater samples and were further defined as deep-sea-distributed molecules in this study (Table 2; Table S3). These screened molecules are shown as van Krevelen diagrams in Fig. 4.

**TABLE 2:** Counts of assigned formulas, relative abundances of CRAM-like molecules, and H/C_a_, O/C_a_, AI_moda_, DBE_a_, and NOSC_a_ values for specific molecule groups after screening

Screened molecules	MF count	MF counts/relative abundance of CRAM-like molecules	H/C_a_	O/C_a_	AI_moda_	DBE_a_	NOSC_a_
TIM	1,775	1,189/66.99%	1.343	0.342	0.258	7.015	−0.643
Persisted molecules	1,290	871/67.52%	1.349	0.343	0.256	6.873	−0.654
Accumulated molecules	638	416/65.20%	1.297	0.338	0.285	7.301	−0.603
Deep sea-distributed molecules	394	269/68.27%	1.307	0.342	0.279	7.211	−0.614

The relative contributions of CRAM and the averages of H/C_a_, O/C_a_, AI_moda_, DBE_a_, and NOSC_a_ of the TIM (1,775 MFs), persistent molecules (1,290 MFs), accumulated molecules (638 MFs), and deep-sea-distributed molecules (394 MFs) are summarized in Table 2. Both the accumulated molecules and deep-sea-distributed molecules showed relatively lower values of H/C_a_ but higher values of AI_moda_, DBE_a_, and NOSC_a_ than those of the persistent molecules and the TIM.

**FIG 5:**
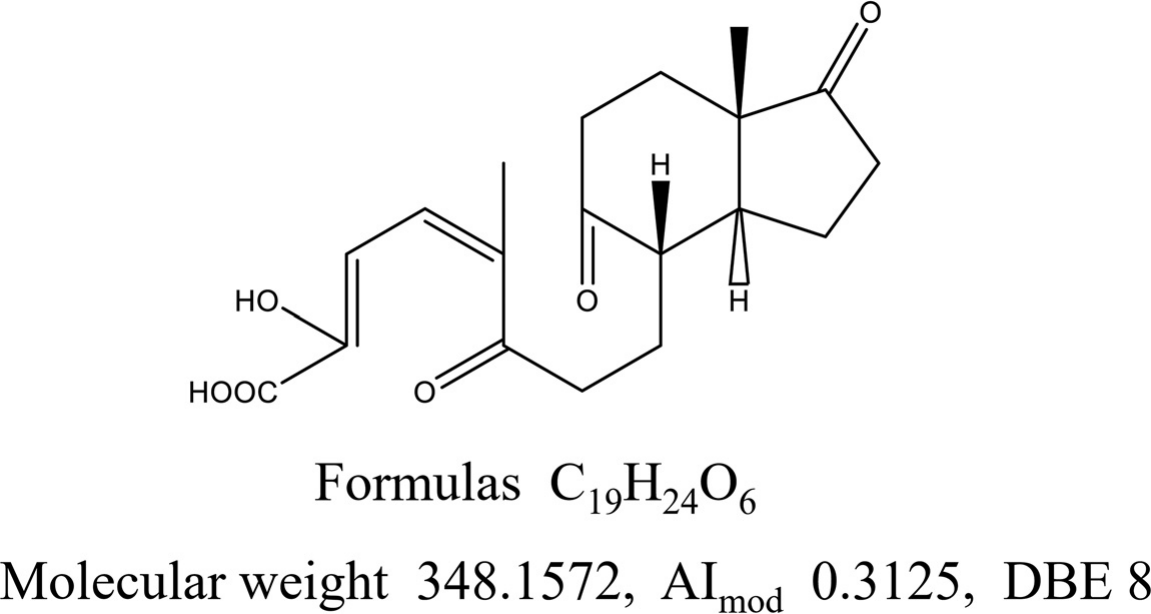
A potential RDOM structure: 5-9,10-diseco-3-hydroxy-5,9,17-trioxoandrosta-1 (10), 2-diene-4-oic acid (4,9-DSHA). The chemical structure of a molecule that accumulated during seawater incubation was also a testosterone catabolite under aerobic circumstances (28).

When the MFs of the TIM were compared with those of the previously established testosterone catabolites (28), 13 MFs in the TIM resembled the previously established testosterone catabolites (Fig. S4). The partial aerobic catabolic pathways of testosterone, the stability of these MFs in our seawater incubation experiments, and a determination of whether coenzyme A (CoA) is involved in the catabolism of these molecules are shown in Fig. S4. Among the 13 MFs, 6 MFs matched formulas of testosterone catabolites provided by Chiang et al. (28). The six MFs and their potential chemical structures are shown in Fig. S5. Three of the six MFs were assigned CRAM-like molecules, as they fell within the FT-ICR-MS boundaries for CRAM (DBE/C, 0.30 to 0.68; DBE/H, 0.20 to 0.95; DBE/O, 0.77 to 1.75) (24). The molecular structures of these six molecules might contain alicyclic rings fused with hydroxyl, acyl, and carboxyl functional groups (Fig. S4). Among them, we have provided a potential chemical structure for a typical CRAM-like molecule (C_19_H_24_O_6_) that showed the highest normalized intensity among the three CRAM-like molecules (Fig. 5). We note that this chemical structure requires further verification.

## DISCUSSION

### Fate of steroids in aquatic environments.

This study characterized the molecular composition of TIM induced by the testosterone degrader strain ATCC 11996, which can be isolated from sewage (29). The 90-day incubation of testosterone with the single strain indicated that testosterone is not as recalcitrant as previously suggested (25) and that microbial degradation can reduce testosterone abundance through respiration and transformation, thereby potentially mitigating the detrimental environmental effects of steroids in aquatic environments.

Ultrahigh-resolution FT-ICR MS revealed that 66.99% of the assigned MFs of the TIM matched the criteria for being categorized as CRAM (24) (Fig. 1). During the verification of the bioavailability of TIM, some MFs of the TIM resisted microbial degradation or even accumulated (Fig. 4). By comparing the TIM with testosterone metabolites provided in known metabolic pathways (30–32), we suggest that the TIM might contain common steroid metabolites, such as 3aα-H-4α (3′-propanoate)-7aβ-methylhexahydro-1,5-indanedione (HIP) and 5-9,10-diseco-3-hydroxy-5,9,17-trioxoandrosta-1 (10), 2-diene-4-oicacid (4,9-DSHA) (Fig. S4). These two molecules matched the criteria that categorize DOM molecules as CRAM. The HIP has been proposed as a crucial interdigitate catabolite for aerobic and anaerobic degradation of steroid compounds (7, 33). The current study demonstrated that anthropogenic contaminants could be microbially transformed into CRAM-like molecules, some of which closely resembled deep sea RDOM molecules and exhibited biological recalcitrance (Fig. 4).

Steroids not only originate from mammals but from diverse marine organisms, especially protists (34). Marine organism-derived steroids could also be transformed into HIP, 4,9-DSHA, and other potential RDOM proxy productions (35). Some microbial clades, including *Glaciecola*, *Marinobacterium*, *Pseudoalteromonas*, *Shewanella*, OM60, and SAR86, are potential steroid degraders (36). These bacteria are widely distributed in seawater and sediments (37–40), thus suggesting that steroid degradation could also occur in seawater and that steroid metabolites could contain organic molecules similar to the TIM characterized in this study. This phenomenon potentially explains the fate of steroids in marine environments.

### Bioavailability of TIM and a potential proxy of bio-recalcitrant CRAM.

In our experiment, some of the assigned CRAM molecules derived from the TIM were bioavailable in natural microbial assemblages (Fig. 2); this experimental result indicated that some CRAM compounds have different bioreactivities (41), especially when they are produced by a single cell, such as the bacteria-derived CRAM in the current study and chemoautotrophy-related CRAM in the study of Bayer et al. (23). Natural deep sea CRAM are generally bio-recalcitrant, which is reasonable, since these molecules have been subjected to multiple biotic and abiotic processes, such as successive processing by distinct microbial communities (42), photochemical degradation (43), priming, and cometabolism during the mixing of water masses (44). This study showed that the screened accumulated molecules (638 MFs) could be relatively bio-refractory compared to the TIM (1,775 MFs). Furthermore, the 638 screened accumulated molecules had higher average AI_mod_, DBE, and NOSC values but lower average H/C ratios than the TIM (Table 2). Higher values of AI_mod_ indicated a higher number of aromatic rings fused to DOM molecules (45). Lower H/C ratios indicated a higher unsaturated state of DOM in the molecules. DBE generally represents the sum of unsaturations plus rings in a molecule, accompanied by higher AI_mod_ values, suggesting the contribution of aromatic and condensed aromatic structures in the DOM samples (45). Relatively high AI_mod_ and DBE values and low H/C ratios have been observed in RDOM samples from the deep sea (22, 46) and long-term incubation studies (22, 47). The average NOSC value of the DOM sample hints at an implicit link with the molecular composition; large amounts of labile lipid and protein substances can be reflected in a low average NOSC value, whereas relatively bio-recalcitrant lignin and condensed hydrocarbons can shift the average NOSC to a high value (48). These indices were accompanied by variations in the DOC concentration (Fig. 2) and FDOM components (Fig. 3) during the 90-day incubation experiment. Overall, we suggest that the bio-recalcitrance of CRAM largely depends on the number of cycles experienced in natural biotic and abiotic processes.

Through the verification of the bioavailability of TIM, a subset of MFs was screened as potential bio-recalcitrant CRAM. In particular, 13 MFs in the TIM assembled previously were established testosterone catabolites (Fig. S4) (28). The chemical structure of a representative MF screened from the 13 MFs is shown in Fig. 5. We note that the chemical structure of CRAM remains ambiguous, because isomers may exist in any molecular formula obtained from FT-ICR MS. Further experiments are required to accurately compare the steroid metabolites and natural deep sea RDOM via a comprehensive fractionation of DOM and their identification using chromatography-applied MS/MS (49). This approach could further verify the consistency of the structural proxy with the RDOM structure in deep seawater.

### Potential fate of CRAM in natural aquatic environments.

The exact chemical structure of CRAM would also improve our understanding of the fate of RDOM in natural water. The recalcitrance and fate of RDOM have been studied for more than half a century (50, 51). An intrinsically recalcitrant DOM hypothesis states that some RDOM molecules are recalcitrant to microbial degradation because of their high chemical structural complexity. In specific environmental contexts, microbes lack specific genes to utilize RDOM molecules, or essential cofactors are lacking to catalyze the further degradation of RDOM (52, 53). CRAM is a group of carboxylate molecules fused with alicyclic structures (24), and the bio-recalcitrance of some CRAM compounds can be explained by the intrinsically recalcitrant hypothesis. Researchers have suggested that CoA (C_21_H_36_N_7_O_16_P_3_S) is a key cofactor in transferring the activated carboxyl group (54), which is the key structure of CRAM. In the steroid degradation process, C-C bonds can be broken by oxygenase or hydratase (30, 32), and the carboxylic acid functional group must be sequentially activated by ATP (C_10_H_16_N_5_O_13_P_3_) and CoA (55).

The recalcitrance of CRAM in the deep ocean might be related to CoA. A previous study also suggested that CoA is a key cofactor involved in the anaerobic degradation of aromatic compounds (56). Pantothenate (vitamin B_5_) is a potential precursor of CoA; however, both pantothenate and CoA are absent in the ocean or would be in much lower concentrations than the limit of detection (57, 58). Therefore, a lack of pantothenate may result in insufficient CoA needed to utilize CRAM and highly aromatic DOM. The relative contribution of CRAM and the values of AI_mod_ generally increase from the surface to deep seawater samples (22, 46), indicating that the molecular abundance of CRAM and highly aromatic DOM accumulate in the deep sea layer. In addition to steroid and aromatic DOM degradation, the β-oxidation process is likely to be involved in the degradation of linear and cyclic terpenoids (MDLT), such as hopanoids and carotenoids, which may produce RDOM with a linear terpenoid structure; for instance, carotenoids are RDOM precursors (59). The β-oxidation pathway must be initiated by CoA and acetyl-CoA. Therefore, we hypothesize that the lack of CoA, and even vitamin B_5_ (the CoA precursor), could be linked with the accumulation of carboxylic carbon compounds to an extent, such as that for aromatic compounds, MDLT analogs, and CRAM. Nevertheless, further experimental evidence is warranted to validate this hypothesis.

## MATERIALS AND METHODS

### Microbial degradation of testosterone.

Testosterone was selected as the sole carbon source in a chemically defined medium (Table S4). A testosterone-degrading strain, Comamonas testosteroni ATCC 11996, was inoculated in the medium and incubated for 90 days in the dark at 30°C. After the 90-day incubation, the culture medium was centrifuged at 10,000 × *g* and 4°C for 10 min, and the supernatant was filtered through precombusted (450°C for 4.5 h) GF/F glass fiber filters (0.7 μm, Whatman). DOM was then extracted from the filtrate using a standard solid-phase extraction (SPE) protocol described by Dittmar et al. (60). In this study, 1 g of styrene divinylbenzene copolymer cartridges (Agilent Bond Elut PPL, USA) was activated with methanol (high-performance liquid chromatography [HPLC] grade, Merck) and rinsed with 6 mL of acidified Milli-Q water (pH 2). The filtrate was acidified to pH 2 using 32% HCl (HPLC grade; Merck) and then passed through the PPL cartridge via gravity; the PPL cartridges were then extensively rinsed with 0.1% (vol/vol) aqueous formic acid solution and dried completely. Following elution with HPLC-grade methanol, the TIM was further analyzed using ultrahigh-resolution FT-ICR MS. Another eluted TIM was dried under ultrapure N_2_ and redissolved in pure water as the carbon source for another 90-day incubation period for bioavailability verification (see below). The extraction efficiency of the TIM was approximately 60%.

### Molecular composition analysis of TIM.

The molecular composition of TIM was analyzed using ultrahigh-resolution FT-ICR MS. Specifically, the DOM extract (TIM) was adjusted to yield an approximate DOC concentration of 25 mM and analyzed using a 9.4T Bruker Apex Ultra FT-ICR-MS coupled with negative-ion Apollo II electrospray ionization (ESI) at a rate of 250 μL/h (61). The operating conditions were as follows: a spray shield voltage of 2.7 kV, capillary column introduced voltage of 4.5 kV, and capillary column end voltage of −320 V. The ion transformation parameter for the quadrupole (Q1) was optimized at *m/z* 200, and the mass range was *m/z* 150 to 800 Da. Each mass spectrum was acquired by conducting 128 single scans with 4 M words to enhance the signal-noise (S/N) ratio (61).

The FT-ICR MS was calibrated using a known homologous series of the Suwannee River natural organic matter sample (obtained from the International Humic Substances Society, USA). Raw spectra of the detected samples are presented in Fig. S1. Mass peaks with an S/N greater than 6 were exported for data analysis using in-house software (61). Briefly, a two-mass scale-expanded segment near the abundant peak of the spectrum was selected, followed by detailed identification of each peak first, and then the peak of at least one of each class of species was arbitrarily selected as a reference peak. The MF assignments used in this study consisted of ^12^C_1–60_, ^1^H_1–120_, ^14^N_0–3_, ^16^O_0–30_, and ^32^S_0–1_ (61). All assigned formulas had to meet the following basic chemical criteria: (i) the number of H atoms was at least 1/3 that of C atoms and did not exceed 2C + N + 2; (ii) the sum of H and N atoms was even (the nitrogen rule); and (iii) the number of N or O atoms did not exceed the number of C atoms.

The H/C, O/C, AI_mod_, DBE, and NOSC were calculated for each assigned formula in each sample (45, 62). These indices were calculated using the following equations: DBE = 1 + 0.5(2C − H + N + P); AI_mod_ = (1 + C − 0.5O − S − 0.5H)/(C − 0.5O − S − N − P); and NOSC = [4 − (4C + H − 3N − 2O + 5P − 2S)]/C.

### Bioavailability verification of TIM in coastal seawater microbial assemblage.

The TIM extracted by SPE (described above in “Microbial degradation of testosterone”) were further amended into seawater with natural microbial assemblages to evaluate the bioavailability. Surface seawater samples were collected from the coastal area (station S05; 24°N 118°E) (Fig. S2) near Xiamen Island on 20 December 2018. The seawater sample was filtered through 3.0-μm polycarbonate filters (Millipore; prerinsed with Milli-Q water) to remove large particles and zooplankton. The incubation experiment was conducted in 10-liter polycarbonate bottles which were covered with aluminum foil to create dark conditions. Treatment groups and control groups were settled in duplicate under 27°C. All bottles were prewashed with acid and rinsed with Milli-Q water. For the incubation, 8 liters of filtered seawater was added to each bottle. The controls were unamended, and the treatment groups received an addition of TIM. The seawater in the control groups contained approximately 75.56 μmol/liter DOC at the beginning. The 0-day TIM treatment groups contained approximately 521.86 μmol/liter DOC.

Subsamples were collected on days 0, 1, 3, 5, 7, and 90 to analyze DOC concentrations and DOM compositions. DOM composition analyses included molecular composition and fluorescent properties. Additional subsamples were collected on days 15, 30, and 60 to analyze the DOC concentrations. Subsamples were taken from all duplicates of both the treatment and control groups. The DOM composition was analyzed using FT-ICR MS; 500-mL seawater samples were filtered with prerinsed 0.2-μm polycarbonate membrane filters (47 mm; Millipore) using a vacuum pump before SPE. The filtrates were acidified to a pH of 2 in 500-mL precombusted glass bottles and then subjected to a standard SPE protocol. The analysis technique used for the molecular composition was the same as that described above for “Molecular composition analysis of TIM.”

### DOC concentration analysis.

Samples (20 mL) for DOC concentration analysis were filtered through precombusted GF/F glass fiber filters (0.7 μm; Whatman) and collected in 40-mL glass vials. Collected samples were acidified with phosphoric acid to pH 2 and stored at −20°C before analysis. The DOC concentration was measured using a Shimadzu TOC-VCPH analyzer with high-temperature (680°C) catalytic oxidation following our previous study (47).

### Excitation-emission matrix fluorescence analysis.

The samples for the FDOM measurements were filtered through the precombusted GF/F glass fiber filters (0.7 μm; Whatman); 4 mL of these samples was stocked in 4-mL glass vials at −20°C. EEM fluorescence was used to characterize the FDOM components. Samples were defrosted and measured using a Varian Cary Eclipse spectrofluorometer. Emission spectra were scanned every 1.78 nm at wavelengths ranging from 83 to 630 nm, with excitation wavelengths ranging from 200 to 800 nm at 2-nm intervals. The samples collected from the same sampling date were blank-corrected using pure water EEMs measured on the same day. EEMs were analyzed and decomposed into individual components using PARAFAC in MATLAB 2019b, coupled with the DOM Fluor toolbox (63).
