# Supplementary material for: Direct Production of Bio-Recalcitrant Carboxyl-Rich Alicyclic Molecules Evidenced in a Bacterium-Induced Steroid Degradation Experiment
Source: Microbiol Spectr. 2023 Feb 6;11(2):e04693-22. doi: 10.1128/spectrum.04693-22 (PMC10100752; doi:10.1128/spectrum.04693-22)
Supplement: Supplemental file 1 — Supplemental material. Download spectrum.04693-22-s0001.pdf, PDF file, 2.1 MB [file spectrum.04693-22-s0001.pdf]

Supplementary Information for

Direct production of bio-recalcitrant carboxyl-rich alicyclic molecules evidenced by a bacterium-induced steroid degradation experiment

Zijing Liu[1], Ruanhong Cai[1,*], Yi-Lung Chen[2], Xiaocun Zhuo[3], Chen He[3], Qiang Zheng[1], Ding He[4], Quan Shi[3], and Nianzhi Jiao[1,*]

[1]State Key Laboratory of Marine Environmental Science and College of Ocean and Earth Sciences, Fujian Key Laboratory of Marine Carbon Sequestration, Xiamen University, Xiamen 361005, China

[2]Department of Microbiology, Soochow University, Taipei, 111, Taiwan

[3]State Key Laboratory of Heavy Oil Processing, China University of Petroleum (Beijing), Beijing 102249, China

[4]Department of Ocean Science and Hong Kong Branch of the Southern Marine Science and Engineering Guangdong Laboratory (Guangzhou), The Hong Kong University of Science and Technology, Hong Kong, China

Zijing Liu and Ruanhong Cai contribute equally to this work.

*Correspondence to Ruanhong Cai (email: crh1987@xmu.edu.cn; Tel.: 86 592 2180199; Fax: 86 592 2180150) or Nianzhi Jiao (jiao@xmu.edu.cn).

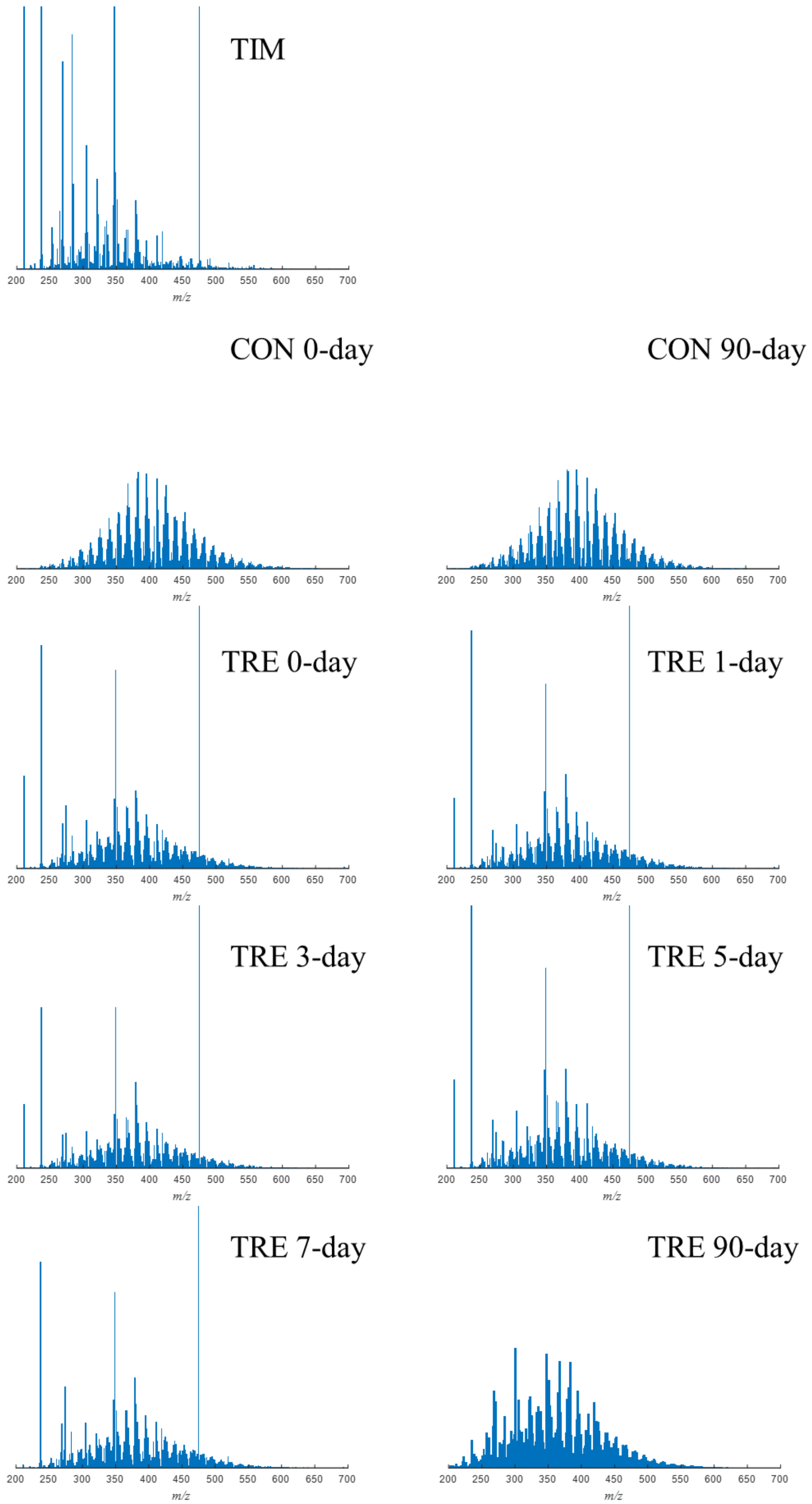

Fig. S1 Raw DOM spectra of TIM. Spectra of the incubations of controls (CON) at days 0 and 90, and TIM-amended treatments (TRE) at days 0, 1, 3, 5, 7, and 90 analyzed using FT-ICR MS were presented.

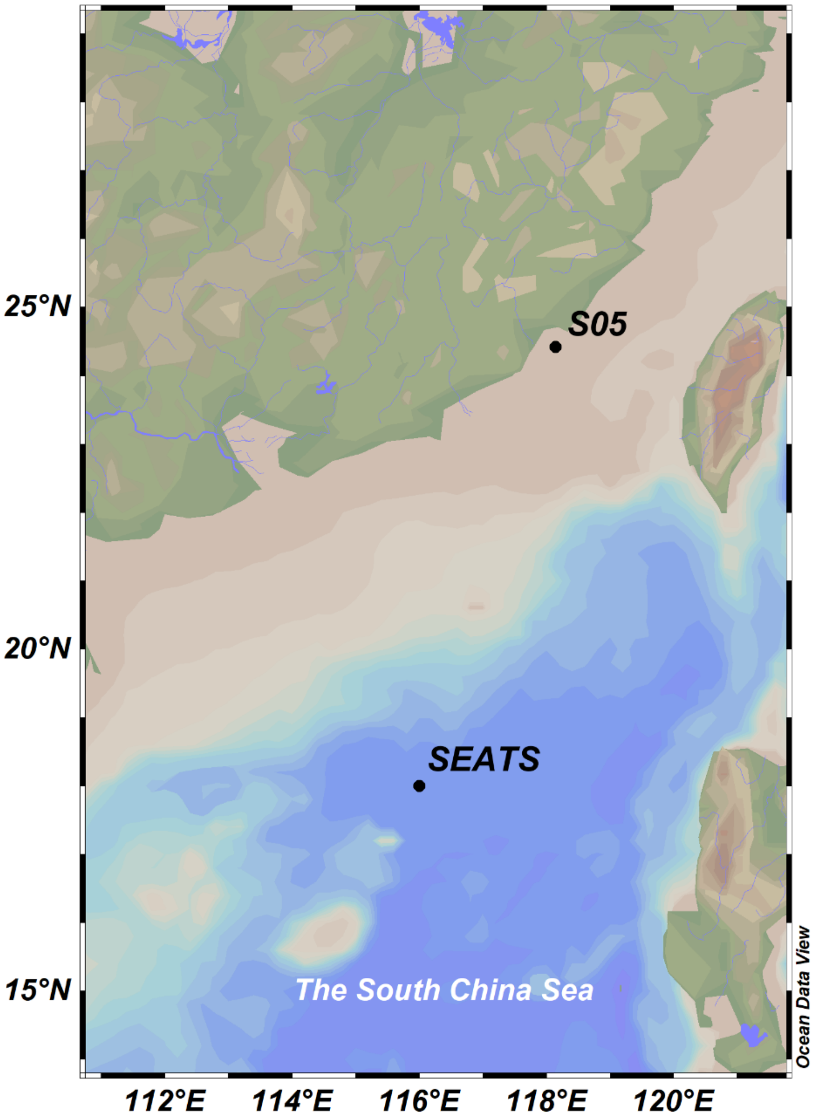

Fig. S2 Locations of natural seawater sample collection. Surface seawater samples were collected from the station S05 (24°N 118°E) off the Xiamen coast in December 2019 and were used for the 90-day incubation experiment. Deep-sea seawater samples were collected from the depths of 500m, 800m, 1000m, and 2000m at the station of the South East Asia Time series Study (SEATS, 18 °N, 116 °E) of the South China Sea in April 2018 and were severed as reference samples for comparison study. Note that data of the four depths DOM samples of the SEATS were previously published in the study of Lian et al. (1)

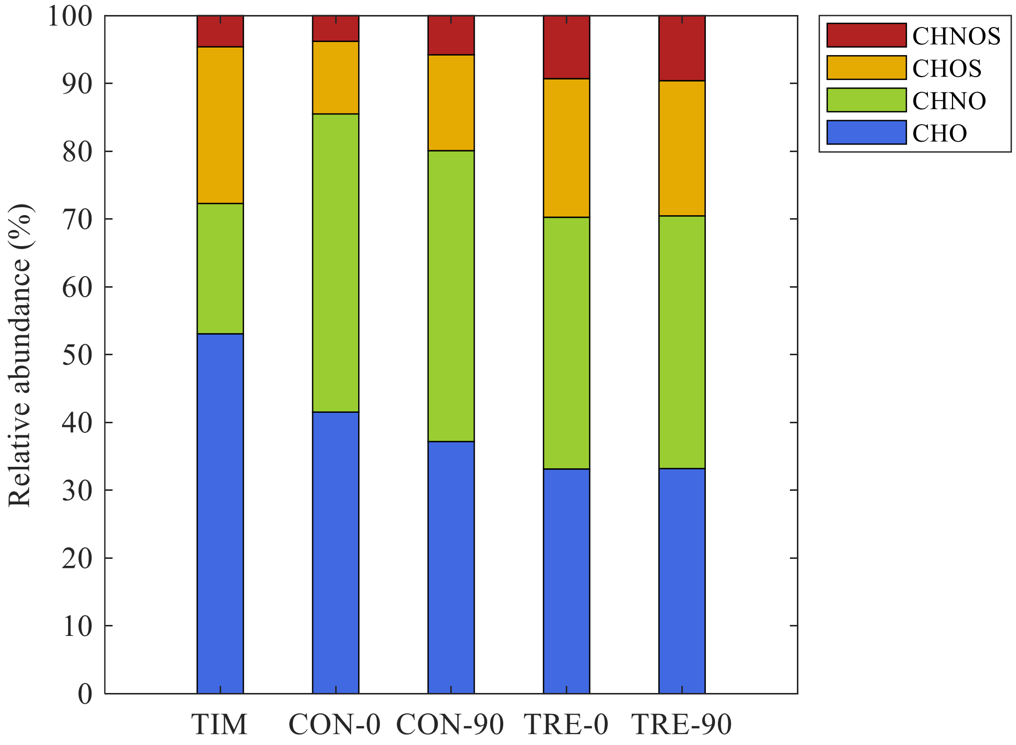

Fig. S3 Molecular formula classes in the testosterone-induce metabolites (TIM), and days 0 and 90 incubations of controls (CON) and TIM-amended treatments (TRE) were analyzed by FT-ICR MS. The relative abundance of molecular formulas was grouped into four classes: CHO, CHNO, CHOS and CHNOS.

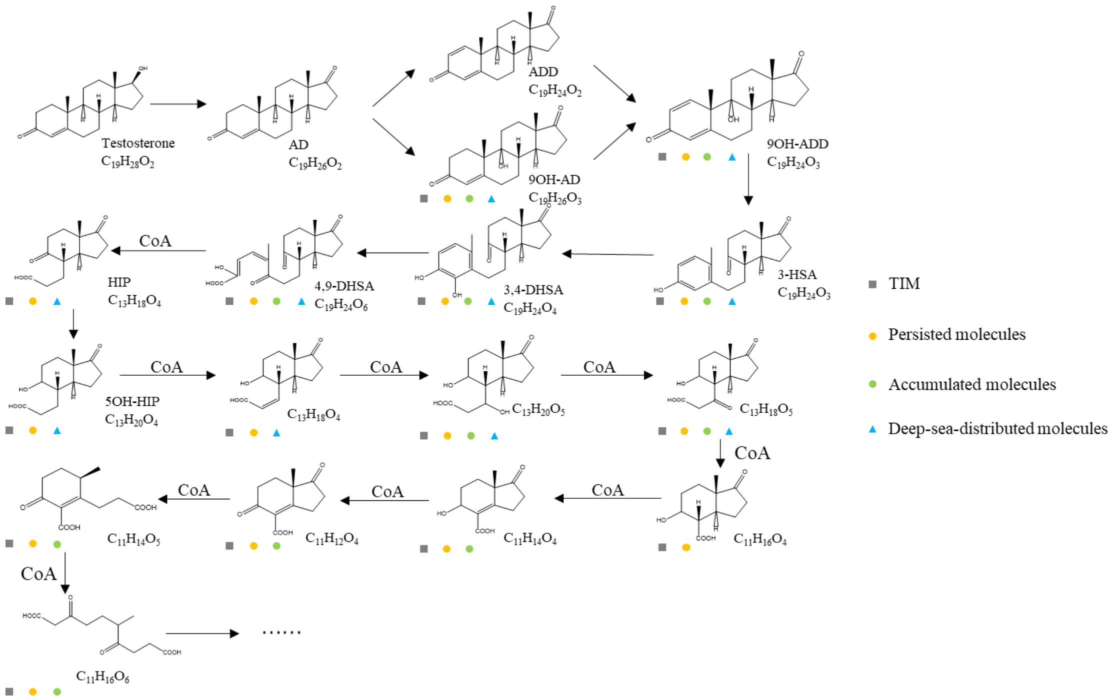

Fig. S4 An aerobic metabolic pathway of testosterone provided in the study of Chiang et al. (2). Labels placed below the formulas of the testosterone metabolic pathway indicated that the formula could exist in the TIM (gray squares), persisted molecules (orange circles), accumulated molecules (green circles), and deep-sea distributed molecules (blue triangles), which were analyzed using FT-ICR MS (Fig. 4 and Table 2).

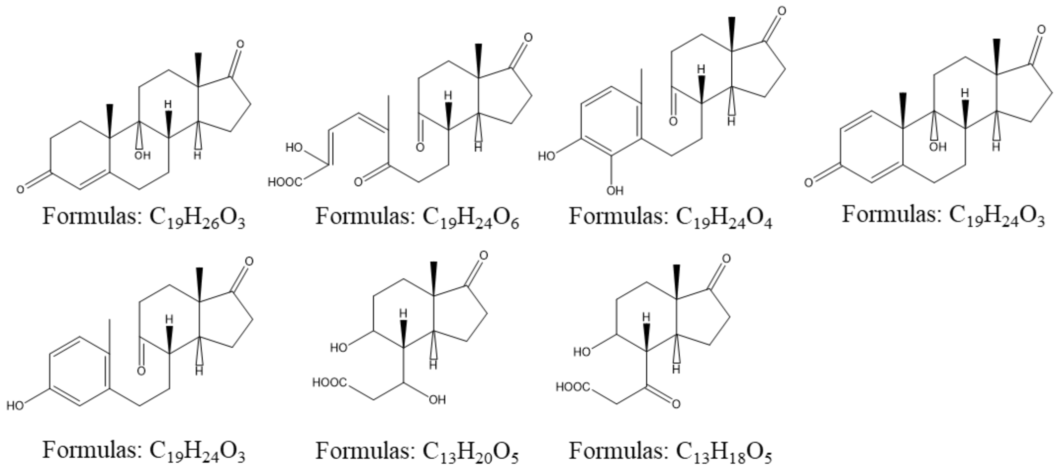

Fig. S5 Six formulas were screened and their potential chemical structures were presented in Chiang et al. (2). These 6 formulas were same with the formulas with previous described testosterone metabolites Chiang et al. 2020, and were found in the TIM, the accumulated molecules during the 90-day incubation, and the DOM samples collected from four depths (500, 800, 1000, and 2000 m) at the station of SEATS (Fig. S2). Note that the formula $C_{19}H_{24}O_3$ could have two isomers, two potential chemical structures were given.

Table S1. Formula, *m/z*, element class, oxygen to carbon ratio (O/C), hydrogen to carbon ratio (H/C), double bond equivalents (DEB), and modified aromatic index (AI_{mod}) of the 1290 persistent molecular formulas existed during the 90-day seawater incubation.

Formula	m/z	Class	O/C	H/C	DBE	AI
C12H14O3	205.0869	CHO	0.250	1.167	6	0.429
C13H18O2	205.1233	CHO	0.154	1.385	5	0.333
C11H12O4	207.0663	CHO	0.364	1.091	6	0.444
C12H16O3	207.1026	CHO	0.250	1.333	5	0.333
C11H14O4	209.0819	CHO	0.364	1.273	5	0.333
C12H18O3	209.1183	CHO	0.250	1.500	4	0.238
C10H12O5	211.0611	CHO	0.500	1.200	5	0.333
C11H16O4	211.0974	CHO	0.364	1.455	4	0.222
C10H14O5	213.0768	CHO	0.500	1.400	4	0.200
C10H16O5	215.0925	CHO	0.500	1.600	3	0.067
C12H10O4	217.0506	CHO	0.333	0.833	8	0.600
C12H12O4	219.0662	CHO	0.333	1.000	7	0.500
C13H16O3	219.1026	CHO	0.231	1.231	6	0.391
C11H10O5	221.0455	CHO	0.455	0.909	7	0.529
C12H14O4	221.0819	CHO	0.333	1.167	6	0.400
C13H18O3	221.1183	CHO	0.231	1.385	5	0.304
C14H22O2	221.1547	CHO	0.143	1.571	4	0.231
C11H12O5	223.0612	CHO	0.455	1.091	6	0.412
C12H16O4	223.0976	CHO	0.333	1.333	5	0.300
C13H20O3	223.1340	CHO	0.231	1.538	4	0.217
C11H14O5	225.0768	CHO	0.455	1.273	5	0.294
C12H18O4	225.1132	CHO	0.333	1.500	4	0.200
C11H16O5	227.0925	CHO	0.455	1.455	4	0.176
C11H18O5	229.1081	CHO	0.455	1.636	3	0.059
C13H12O4	231.0662	CHO	0.308	0.923	8	0.545
C14H16O3	231.1027	CHO	0.214	1.143	7	0.440
C15H20O2	231.1390	CHO	0.133	1.333	6	0.357
C12H10O5	233.0455	CHO	0.417	0.833	8	0.579
C13H14O4	233.0819	CHO	0.308	1.077	7	0.455
C14H18O3	233.1183	CHO	0.214	1.286	6	0.360
C15H22O2	233.1547	CHO	0.133	1.467	5	0.286
C12H12O5	235.0612	CHO	0.417	1.000	7	0.474
C13H16O4	235.0976	CHO	0.308	1.231	6	0.364
C14H20O3	235.1340	CHO	0.214	1.429	5	0.280
C11H10O6	237.0405	CHO	0.545	0.909	7	0.500
C12H14O5	237.0768	CHO	0.417	1.167	6	0.368
C13H18O4	237.1129	CHO	0.308	1.385	5	0.273
C14H22O3	237.1496	CHO	0.214	1.571	4	0.200
C11H13N1O5	238.0721	CHNO	0.455	1.182	6	0.400
C11H12O6	239.0561	CHO	0.545	1.091	6	0.375
C12H16O5	239.0925	CHO	0.417	1.333	5	0.263
C13H20O4	239.1288	CHO	0.308	1.538	4	0.182
C11H15N1O5	240.0878	CHNO	0.455	1.364	5	0.267
C11H14O6	241.0717	CHO	0.545	1.273	5	0.250
C12H18O5	241.1081	CHO	0.417	1.500	4	0.158
C16H18O2	241.1234	CHO	0.125	1.125	8	0.467

C10H12O5S1	243.0332	CHOS	0.500	1.200	5	0.231
C11H16O6	243.0874	CHO	0.545	1.455	4	0.125
C15H16O3	243.1027	CHO	0.200	1.067	8	0.481
C12H20O5	243.1237	CHO	0.417	1.667	3	0.053
C16H20O2	243.1390	CHO	0.125	1.250	7	0.400
C10H14O5S1	245.0489	CHOS	0.500	1.400	4	0.077
C14H14O4	245.0819	CHO	0.286	1.000	8	0.500
C11H18O6	245.1030	CHO	0.545	1.636	3	0.000
C15H18O3	245.1183	CHO	0.200	1.200	7	0.407
C16H22O2	245.1547	CHO	0.125	1.375	6	0.333
C13H13N1O4	246.0772	CHNO	0.308	1.000	8	0.550
C9H12O6S1	247.0282	CHOS	0.667	1.333	4	0.000
C13H12O5	247.0612	CHO	0.385	0.923	8	0.524
C14H16O4	247.0976	CHO	0.286	1.143	7	0.417
C15H20O3	247.1340	CHO	0.200	1.333	6	0.333
C16H24O2	247.1703	CHO	0.125	1.500	5	0.267
C12H10O6	249.0404	CHO	0.500	0.833	8	0.556
C13H14O5	249.0768	CHO	0.385	1.077	7	0.429
C14H18O4	249.1132	CHO	0.286	1.286	6	0.333
C15H22O3	249.1496	CHO	0.200	1.467	5	0.259
C12H13N1O5	250.0721	CHNO	0.417	1.083	7	0.471
C12H12O6	251.0561	CHO	0.500	1.000	7	0.444
C13H16O5	251.0925	CHO	0.385	1.231	6	0.333
C14H20O4	251.1289	CHO	0.286	1.429	5	0.250
C12H14O6	253.0718	CHO	0.500	1.167	6	0.333
C13H18O5	253.1082	CHO	0.385	1.385	5	0.238
C14H22O4	253.1446	CHO	0.286	1.571	4	0.167
C12H16O6	255.0874	CHO	0.500	1.333	5	0.222
C13H20O5	255.1238	CHO	0.385	1.538	4	0.143
C11H15N1O6	256.0827	CHNO	0.545	1.364	5	0.214
C11H14O5S1	257.0489	CHOS	0.455	1.273	5	0.200
C11H14O7	257.0667	CHO	0.636	1.273	5	0.200
C12H18O6	257.1030	CHO	0.500	1.500	4	0.111
C16H18O3	257.1183	CHO	0.188	1.125	8	0.448
C13H22O5	257.1394	CHO	0.385	1.692	3	0.048
C17H22O2	257.1547	CHO	0.118	1.294	7	0.375
C10H12O6S1	259.0282	CHOS	0.600	1.200	5	0.167
C15H16O4	259.0976	CHO	0.267	1.067	8	0.462
C12H20O6	259.1188	CHO	0.500	1.667	3	0.000
C16H20O3	259.1340	CHO	0.188	1.250	7	0.379
C17H24O2	259.1703	CHO	0.118	1.412	6	0.313
C10H14O6S1	261.0438	CHOS	0.600	1.400	4	0.000
C14H14O5	261.0768	CHO	0.357	1.000	8	0.478
C15H18O4	261.1132	CHO	0.267	1.200	7	0.385
C16H22O3	261.1496	CHO	0.188	1.375	6	0.310
C14H17N1O4	262.1085	CHNO	0.286	1.214	7	0.409
C13H12O6	263.0561	CHO	0.462	0.923	8	0.500
C14H16O5	263.0925	CHO	0.357	1.143	7	0.391
C15H20O4	263.1289	CHO	0.267	1.333	6	0.308
C16H24O3	263.1653	CHO	0.188	1.500	5	0.241

C13H15N1O5	264.0878	CHNO	0.385	1.154	7	0.421
C12H10O7	265.0354	CHO	0.583	0.833	8	0.529
C13H14O6	265.0717	CHO	0.462	1.077	7	0.400
C14H18O5	265.1081	CHO	0.357	1.286	6	0.304
C15H22O4	265.1445	CHO	0.267	1.467	5	0.231
C13H17N1O5	266.1034	CHNO	0.385	1.308	6	0.316
C12H12O7	267.0511	CHO	0.583	1.000	7	0.412
C13H16O6	267.0874	CHO	0.462	1.231	6	0.300
C14H20O5	267.1238	CHO	0.357	1.429	5	0.217
C15H24O4	267.1602	CHO	0.267	1.600	4	0.154
C13H19N1O5	268.1190	CHNO	0.385	1.462	5	0.211
C12H14O7	269.0667	CHO	0.583	1.167	6	0.294
C13H18O6	269.1030	CHO	0.462	1.385	5	0.200
C17H18O3	269.1183	CHO	0.176	1.059	9	0.484
C14H22O5	269.1394	CHO	0.357	1.571	4	0.130
C15H26O4	269.1758	CHO	0.267	1.733	3	0.077
C12H17N1O6	270.0984	CHNO	0.500	1.417	5	0.188
C12H16O5S1	271.0646	CHOS	0.417	1.333	5	0.176
C12H16O7	271.0824	CHO	0.583	1.333	5	0.176
C16H16O4	271.0976	CHO	0.250	1.000	9	0.500
C13H20O6	271.1187	CHO	0.462	1.538	4	0.100
C17H20O3	271.1340	CHO	0.176	1.176	8	0.419
C14H24O5	271.1552	CHO	0.357	1.714	3	0.043
C12H19N1O6	272.1139	CHNO	0.500	1.583	4	0.063
C11H14O6S1	273.0438	CHOS	0.545	1.273	5	0.143
C15H14O5	273.0769	CHO	0.333	0.933	9	0.520
C12H18O7	273.0979	CHO	0.583	1.500	4	0.059
C16H18O4	273.1132	CHO	0.250	1.125	8	0.429
C13H22O6	273.1344	CHO	0.462	1.692	3	0.000
C17H22O3	273.1496	CHO	0.176	1.294	7	0.355
C14H13N1O5	274.0721	CHNO	0.357	0.929	9	0.571
C15H17N1O4	274.1084	CHNO	0.267	1.133	8	0.458
C14H12O6	275.0561	CHO	0.429	0.857	9	0.545
C11H16O6S1	275.0594	CHOS	0.545	1.455	4	0.000
C15H16O5	275.0925	CHO	0.333	1.067	8	0.440
C12H20O7	275.1135	CHO	0.583	1.667	3	-0.059
C16H20O4	275.1289	CHO	0.250	1.250	7	0.357
C17H24O3	275.1653	CHO	0.176	1.412	6	0.290
C10H14O7S1	277.0388	CHOS	0.700	1.400	4	-0.091
C14H14O6	277.0718	CHO	0.429	1.000	8	0.455
C15H18O5	277.1082	CHO	0.333	1.200	7	0.360
C16H22O4	277.1445	CHO	0.250	1.375	6	0.286
C17H26O3	277.1810	CHO	0.176	1.529	5	0.226
C14H17N1O5	278.1034	CHNO	0.357	1.214	7	0.381
C13H12O7	279.0510	CHO	0.538	0.923	8	0.474
C14H16O6	279.0874	CHO	0.429	1.143	7	0.364
C15H20O5	279.1238	CHO	0.333	1.333	6	0.280
C16H24O4	279.1602	CHO	0.250	1.500	5	0.214
C14H19N1O5	280.1191	CHNO	0.357	1.357	6	0.286
C13H14O7	281.0667	CHO	0.538	1.077	7	0.368

C14H18O6	281.1031	CHO	0.429	1.286	6	0.273
C15H22O5	281.1395	CHO	0.333	1.467	5	0.200
C16H26O4	281.1759	CHO	0.250	1.625	4	0.143
C14H21N1O5	282.1347	CHNO	0.357	1.500	5	0.190
C12H12O6S1	283.0283	CHOS	0.500	1.000	7	0.375
C13H16O5S1	283.0646	CHOS	0.385	1.231	6	0.263
C13H16O7	283.0824	CHO	0.538	1.231	6	0.263
C17H16O4	283.0976	CHO	0.235	0.941	10	0.533
C14H20O6	283.1188	CHO	0.429	1.429	5	0.182
C18H20O3	283.1341	CHO	0.167	1.111	9	0.455
C15H24O5	283.1551	CHO	0.333	1.600	4	0.120
C13H19N1O6	284.1140	CHNO	0.462	1.462	5	0.167
C12H14O6S1	285.0439	CHOS	0.500	1.167	6	0.250
C12H14O8	285.0617	CHO	0.667	1.167	6	0.250
C16H14O5	285.0768	CHO	0.313	0.875	10	0.556
C13H18O5S1	285.0803	CHOS	0.385	1.385	5	0.158
C13H18O7	285.0980	CHO	0.538	1.385	5	0.158
C17H18O4	285.1133	CHO	0.235	1.059	9	0.467
C14H22O6	285.1344	CHO	0.429	1.571	4	0.091
C18H22O3	285.1496	CHO	0.167	1.222	8	0.394
C15H26O5	285.1708	CHO	0.333	1.733	3	0.040
C12H16O8	287.0773	CHO	0.667	1.333	5	0.125
C16H16O5	287.0925	CHO	0.313	1.000	9	0.481
C13H20O7	287.1136	CHO	0.538	1.538	4	0.053
C17H20O4	287.1289	CHO	0.235	1.176	8	0.400
C14H24O6	287.1500	CHO	0.429	1.714	3	0.000
C18H24O3	287.1653	CHO	0.167	1.333	7	0.333
C16H19N1O4	288.1241	CHNO	0.250	1.188	8	0.423
C15H14O6	289.0717	CHO	0.400	0.933	9	0.500
C12H18O6S1	289.0751	CHOS	0.500	1.500	4	0.000
C14H14N2O5	289.0833	CHNO	0.357	1.000	9	0.579
C16H18O5	289.1081	CHO	0.313	1.125	8	0.407
C17H22O4	289.1445	CHO	0.235	1.294	7	0.333
C18H26O3	289.1809	CHO	0.167	1.444	6	0.273
C15H17N1O5	290.1034	CHNO	0.333	1.133	8	0.435
C14H12O7	291.0509	CHO	0.500	0.857	9	0.524
C11H16O7S1	291.0543	CHOS	0.636	1.455	4	-0.077
C15H16O4S1	291.0696	CHOS	0.267	1.067	8	0.417
C15H16O6	291.0874	CHO	0.400	1.067	8	0.417
C16H20O5	291.1238	CHO	0.313	1.250	7	0.333
C17H24O4	291.1602	CHO	0.235	1.412	6	0.267
C14H15N1O6	292.0827	CHNO	0.429	1.071	8	0.450
C15H19N1O5	292.1190	CHNO	0.333	1.267	7	0.348
C14H14O7	293.0667	CHO	0.500	1.000	8	0.429
C15H18O6	293.1031	CHO	0.400	1.200	7	0.333
C16H22O5	293.1394	CHO	0.313	1.375	6	0.259
C17H26O4	293.1758	CHO	0.235	1.529	5	0.200
C14H17N1O6	294.0983	CHNO	0.429	1.214	7	0.350
C15H21N1O5	294.1347	CHNO	0.333	1.400	6	0.261
C14H16O5S1	295.0646	CHOS	0.357	1.143	7	0.333

C14H16O7	295.0823	CHO	0.500	1.143	7	0.333
C18H16O4	295.0976	CHO	0.222	0.889	11	0.563
C15H20O6	295.1187	CHO	0.400	1.333	6	0.250
C16H24O5	295.1551	CHO	0.313	1.500	5	0.185
C17H28O4	295.1915	CHO	0.235	1.647	4	0.133
C14H19N1O6	296.1140	CHNO	0.429	1.357	6	0.250
C13H14O6S1	297.0438	CHOS	0.462	1.077	7	0.333
C13H14O8	297.0615	CHO	0.615	1.077	7	0.333
C14H18O5S1	297.0802	CHOS	0.357	1.286	6	0.238
C14H18O7	297.0980	CHO	0.500	1.286	6	0.238
C18H18O4	297.1133	CHO	0.222	1.000	10	0.500
C15H22O6	297.1344	CHO	0.400	1.467	5	0.167
C19H22O3	297.1496	CHO	0.158	1.158	9	0.429
C16H26O5	297.1708	CHO	0.313	1.625	4	0.111
C14H21N1O6	298.1296	CHNO	0.429	1.500	5	0.150
C13H16O6S1	299.0595	CHOS	0.462	1.231	6	0.222
C13H16O8	299.0772	CHO	0.615	1.231	6	0.222
C17H16O5	299.0925	CHO	0.294	0.941	10	0.517
C14H20O5S1	299.0959	CHOS	0.357	1.429	5	0.143
C14H20O7	299.1136	CHO	0.500	1.429	5	0.143
C18H20O4	299.1289	CHO	0.222	1.111	9	0.438
C15H24O6	299.1500	CHO	0.400	1.600	4	0.083
C19H24O3	299.1653	CHO	0.158	1.263	8	0.371
C16H28O5	299.1865	CHO	0.313	1.750	3	0.037
C16H14O6	301.0718	CHO	0.375	0.875	10	0.538
C13H18O6S1	301.0751	CHOS	0.462	1.385	5	0.111
C13H18O8	301.0929	CHO	0.615	1.385	5	0.111
C17H18O5	301.1081	CHO	0.294	1.059	9	0.448
C14H22O5S1	301.1115	CHOS	0.357	1.571	4	0.048
C14H22O7	301.1293	CHO	0.500	1.571	4	0.048
C18H22O4	301.1445	CHO	0.222	1.222	8	0.375
C15H26O6	301.1657	CHO	0.400	1.733	3	0.000
C19H26O3	301.1809	CHO	0.158	1.368	7	0.314
C16H17N1O5	302.1034	CHNO	0.313	1.063	9	0.480
C17H21N1O4	302.1398	CHNO	0.235	1.235	8	0.393
C15H12O7	303.0510	CHO	0.467	0.800	10	0.565
C12H16O7S1	303.0544	CHOS	0.583	1.333	5	0.067
C16H16O6	303.0874	CHO	0.375	1.000	9	0.462
C13H20O6S1	303.0908	CHOS	0.462	1.538	4	0.000
C15H16N2O5	303.0985	CHNO	0.333	1.067	9	0.524
C13H20O8	303.1085	CHO	0.615	1.538	4	0.000
C17H20O5	303.1238	CHO	0.294	1.176	8	0.379
C14H24O7	303.1449	CHO	0.500	1.714	3	-0.048
C18H24O4	303.1602	CHO	0.222	1.333	7	0.313
C16H19N1O5	304.1190	CHNO	0.313	1.188	8	0.400
C17H23N1O4	304.1554	CHNO	0.235	1.353	7	0.321
C11H14O8S1	305.0336	CHOS	0.727	1.273	5	0.000
C15H14O7	305.0666	CHO	0.467	0.933	9	0.478
C12H18O7S1	305.0700	CHOS	0.583	1.500	4	-0.067
C16H18O6	305.1030	CHO	0.375	1.125	8	0.385

C15H18N2O5	305.1142	CHNO	0.333	1.200	8	0.429
C17H22O5	305.1394	CHO	0.294	1.294	7	0.310
C18H26O4	305.1758	CHO	0.222	1.444	6	0.250
C15H17N1O6	306.0983	CHNO	0.400	1.133	8	0.409
C16H21N1O5	306.1347	CHNO	0.313	1.313	7	0.320
C14H12O8	307.0460	CHO	0.571	0.857	9	0.500
C11H16O8S1	307.0493	CHOS	0.727	1.455	4	-0.167
C15H16O7	307.0823	CHO	0.467	1.067	8	0.391
C16H20O6	307.1187	CHO	0.375	1.250	7	0.308
C17H24O5	307.1550	CHO	0.294	1.412	6	0.241
C18H28O4	307.1915	CHO	0.222	1.556	5	0.188
C15H19N1O6	308.1140	CHNO	0.400	1.267	7	0.318
C16H23N1O5	308.1503	CHNO	0.313	1.438	6	0.240
C14H14O6S1	309.0439	CHOS	0.429	1.000	8	0.400
C14H14O8	309.0616	CHO	0.571	1.000	8	0.400
C15H18O7	309.0980	CHO	0.467	1.200	7	0.304
C16H22O6	309.1344	CHO	0.375	1.375	6	0.231
C17H26O5	309.1708	CHO	0.294	1.529	5	0.172
C14H17N1O7	310.0933	CHNO	0.500	1.214	7	0.316
C15H21N1O6	310.1296	CHNO	0.400	1.400	6	0.227
C14H16O8	311.0773	CHO	0.571	1.143	7	0.300
C18H16O5	311.0925	CHO	0.278	0.889	11	0.548
C15H20O5S1	311.0960	CHOS	0.333	1.333	6	0.217
C15H20O7	311.1137	CHO	0.467	1.333	6	0.217
C19H20O4	311.1290	CHO	0.211	1.053	10	0.471
C16H24O6	311.1501	CHO	0.375	1.500	5	0.154
C17H28O5	311.1865	CHO	0.294	1.647	4	0.103
C14H19N1O7	312.1090	CHNO	0.500	1.357	6	0.211
C15H23N1O6	312.1453	CHNO	0.400	1.533	5	0.136
C13H14O7S1	313.0388	CHOS	0.538	1.077	7	0.294
C17H14O6	313.0719	CHO	0.353	0.824	11	0.571
C14H18O6S1	313.0752	CHOS	0.429	1.286	6	0.200
C14H18O8	313.0930	CHO	0.571	1.286	6	0.200
C18H18O5	313.1082	CHO	0.278	1.000	10	0.484
C15H22O5S1	313.1116	CHOS	0.333	1.467	5	0.130
C15H22O7	313.1294	CHO	0.467	1.467	5	0.130
C19H22O4	313.1446	CHO	0.211	1.158	9	0.412
C16H26O6	313.1657	CHO	0.375	1.625	4	0.077
C17H17N1O5	314.1034	CHNO	0.294	1.000	10	0.519
C13H16O7S1	315.0544	CHOS	0.538	1.231	6	0.176
C17H16O6	315.0875	CHO	0.353	0.941	10	0.500
C14H20O6S1	315.0910	CHOS	0.429	1.429	5	0.100
C14H20O8	315.1086	CHO	0.571	1.429	5	0.100
C18H20O5	315.1238	CHO	0.278	1.111	9	0.419
C15H24O7	315.1450	CHO	0.467	1.600	4	0.043
C19H24O4	315.1602	CHO	0.211	1.263	8	0.353
C17H19N1O5	316.1190	CHNO	0.294	1.118	9	0.444
C16H14O7	317.0666	CHO	0.438	0.875	10	0.520
C13H18O7S1	317.0700	CHOS	0.538	1.385	5	0.059
C13H18O9	317.0877	CHO	0.692	1.385	5	0.059

C17H18O6	317.1030	CHO	0.353	1.059	9	0.429
C14H22O6S1	317.1064	CHOS	0.429	1.571	4	0.000
C16H18N2O5	317.1144	CHNO	0.313	1.125	9	0.478
C14H22O8	317.1241	CHO	0.571	1.571	4	0.000
C18H22O5	317.1394	CHO	0.278	1.222	8	0.355
C15H26O7	317.1605	CHO	0.467	1.733	3	-0.043
C19H26O4	317.1758	CHO	0.211	1.368	7	0.294
C16H17N1O6	318.0982	CHNO	0.375	1.063	9	0.458
C17H21N1O5	318.1347	CHNO	0.294	1.235	8	0.370
C15H12O8	319.0459	CHO	0.533	0.800	10	0.545
C16H16O7	319.0823	CHO	0.438	1.000	9	0.440
C13H20O7S1	319.0857	CHOS	0.538	1.538	4	-0.059
C15H16N2O6	319.0937	CHNO	0.400	1.067	9	0.500
C17H20O6	319.1187	CHO	0.353	1.176	8	0.357
C16H20N2O5	319.1299	CHNO	0.313	1.250	8	0.391
C14H24O8	319.1399	CHO	0.571	1.714	3	-0.100
C18H24O5	319.1551	CHO	0.278	1.333	7	0.290
C19H28O4	319.1915	CHO	0.211	1.474	6	0.235
C16H19N1O6	320.1141	CHNO	0.375	1.188	8	0.375
C17H23N1O5	320.1504	CHNO	0.294	1.353	7	0.296
C15H14O8	321.0617	CHO	0.533	0.933	9	0.455
C12H18O8S1	321.0650	CHOS	0.667	1.500	4	-0.143
C16H18O7	321.0981	CHO	0.438	1.125	8	0.360
C17H22O6	321.1344	CHO	0.353	1.294	7	0.286
C18H26O5	321.1708	CHO	0.278	1.444	6	0.226
C19H30O4	321.2073	CHO	0.211	1.579	5	0.176
C15H17N1O7	322.0933	CHNO	0.467	1.133	8	0.381
C16H21N1O6	322.1297	CHNO	0.375	1.313	7	0.292
C17H25N1O5	322.1661	CHNO	0.294	1.471	6	0.222
C15H16O6S1	323.0595	CHOS	0.400	1.067	8	0.364
C15H16O8	323.0773	CHO	0.533	1.067	8	0.364
C16H20O7	323.1136	CHO	0.438	1.250	7	0.280
C17H24O6	323.1500	CHO	0.353	1.412	6	0.214
C18H28O5	323.1864	CHO	0.278	1.556	5	0.161
C15H19N1O7	324.1088	CHNO	0.467	1.267	7	0.286
C16H23N1O6	324.1452	CHNO	0.375	1.438	6	0.208
C14H14O7S1	325.0387	CHOS	0.500	1.000	8	0.368
C15H18O6S1	325.0751	CHOS	0.400	1.200	7	0.273
C15H18O8	325.0929	CHO	0.533	1.200	7	0.273
C16H22O5S1	325.1115	CHOS	0.313	1.375	6	0.200
C16H22O7	325.1293	CHO	0.438	1.375	6	0.200
C20H22O4	325.1445	CHO	0.200	1.100	10	0.444
C17H26O6	325.1656	CHO	0.353	1.529	5	0.143
C18H30O5	325.2020	CHO	0.278	1.667	4	0.097
C16H25N1O6	326.1609	CHNO	0.375	1.563	5	0.125
C14H16O7S1	327.0545	CHOS	0.500	1.143	7	0.263
C18H16O6	327.0875	CHO	0.333	0.889	11	0.533
C15H20O6S1	327.0909	CHOS	0.400	1.333	6	0.182
C15H20O8	327.1086	CHO	0.533	1.333	6	0.182
C19H20O5	327.1239	CHO	0.263	1.053	10	0.455

C16H24O5S1	327.1273	CHOS	0.313	1.500	5	0.120
C16H24O7	327.1450	CHO	0.438	1.500	5	0.120
C20H24O4	327.1602	CHO	0.200	1.200	9	0.389
C17H28O6	327.1814	CHO	0.353	1.647	4	0.071
C18H19N1O5	328.1191	CHNO	0.278	1.056	10	0.483
C14H18O7S1	329.0699	CHOS	0.500	1.286	6	0.158
C14H18O9	329.0877	CHO	0.643	1.286	6	0.158
C18H18O6	329.1031	CHO	0.333	1.000	10	0.467
C15H22O6S1	329.1064	CHOS	0.400	1.467	5	0.091
C15H22O8	329.1242	CHO	0.533	1.467	5	0.091
C19H22O5	329.1395	CHO	0.263	1.158	9	0.394
C16H26O7	329.1606	CHO	0.438	1.625	4	0.040
C20H26O4	329.1759	CHO	0.200	1.300	8	0.333
C17H30O6	329.1970	CHO	0.353	1.765	3	0.000
C18H21N1O5	330.1347	CHNO	0.278	1.167	9	0.414
C13H16O8S1	331.0494	CHOS	0.615	1.231	6	0.125
C17H16O7	331.0824	CHO	0.412	0.941	10	0.481
C14H20O7S1	331.0857	CHOS	0.500	1.429	5	0.053
C16H16N2O6	331.0936	CHNO	0.375	1.000	10	0.545
C14H20O9	331.1036	CHO	0.643	1.429	5	0.053
C18H20O6	331.1187	CHO	0.333	1.111	9	0.400
C15H24O6S1	331.1219	CHOS	0.400	1.600	4	0.000
C17H20N2O5	331.1300	CHNO	0.294	1.176	9	0.440
C15H24O8	331.1398	CHO	0.533	1.600	4	0.000
C19H24O5	331.1551	CHO	0.263	1.263	8	0.333
C16H28O7	331.1762	CHO	0.438	1.750	3	-0.040
C20H28O4	331.1915	CHO	0.200	1.400	7	0.278
C17H19N1O6	332.1141	CHNO	0.353	1.118	9	0.423
C18H23N1O5	332.1504	CHNO	0.278	1.278	8	0.345
C16H14O8	333.0617	CHO	0.500	0.875	10	0.500
C13H18O8S1	333.0650	CHOS	0.615	1.385	5	0.000
C17H18O7	333.0981	CHO	0.412	1.059	9	0.407
C14H22O7S1	333.1014	CHOS	0.500	1.571	4	-0.053
C14H22O9	333.1191	CHO	0.643	1.571	4	-0.053
C18H22O6	333.1344	CHO	0.333	1.222	8	0.333
C17H22N2O5	333.1457	CHNO	0.294	1.294	8	0.360
C15H26O8	333.1556	CHO	0.533	1.733	3	-0.091
C19H26O5	333.1708	CHO	0.263	1.368	7	0.273
C20H30O4	333.2072	CHO	0.200	1.500	6	0.222
C16H17N1O7	334.0934	CHNO	0.438	1.063	9	0.435
C17H21N1O6	334.1296	CHNO	0.353	1.235	8	0.346
C18H25N1O5	334.1660	CHNO	0.278	1.389	7	0.276
C16H16O8	335.0772	CHO	0.500	1.000	9	0.417
C13H20O8S1	335.0806	CHOS	0.615	1.538	4	-0.125
C17H20O7	335.1136	CHO	0.412	1.176	8	0.333
C14H24O7S1	335.1169	CHOS	0.500	1.714	3	-0.158
C18H24O4S1	335.1320	CHOS	0.222	1.333	7	0.267
C18H24O6	335.1500	CHO	0.333	1.333	7	0.267
C19H28O5	335.1864	CHO	0.263	1.474	6	0.212
C16H19N1O7	336.1089	CHNO	0.438	1.188	8	0.348

C17H23N1O6	336.1452	CHNO	0.353	1.353	7	0.269
C18H27N1O5	336.1816	CHNO	0.278	1.500	6	0.207
C16H18O6S1	337.0752	CHOS	0.375	1.125	8	0.333
C16H18O8	337.0930	CHO	0.500	1.125	8	0.333
C17H22O7	337.1294	CHO	0.412	1.294	7	0.259
C18H26O6	337.1657	CHO	0.333	1.444	6	0.200
C19H30O5	337.2021	CHO	0.263	1.579	5	0.152
C16H21N1O7	338.1246	CHNO	0.438	1.313	7	0.261
C17H25N1O6	338.1610	CHNO	0.353	1.471	6	0.192
C15H16O7S1	339.0544	CHOS	0.467	1.067	8	0.333
C15H16O9	339.0721	CHO	0.600	1.067	8	0.333
C16H20O6S1	339.0907	CHOS	0.375	1.250	7	0.250
C16H20O8	339.1085	CHO	0.500	1.250	7	0.250
C20H20O5	339.1238	CHO	0.250	1.000	11	0.486
C17H24O7	339.1449	CHO	0.412	1.412	6	0.185
C21H24O4	339.1602	CHO	0.190	1.143	10	0.421
C18H28O6	339.1813	CHO	0.333	1.556	5	0.133
C16H23N1O7	340.1402	CHNO	0.438	1.438	6	0.174
C15H18O7S1	341.0702	CHOS	0.467	1.200	7	0.238
C15H18O9	341.0879	CHO	0.600	1.200	7	0.238
C19H18O6	341.1032	CHO	0.316	0.947	11	0.500
C16H22O6S1	341.1064	CHOS	0.375	1.375	6	0.167
C16H22O8	341.1242	CHO	0.500	1.375	6	0.167
C20H22O5	341.1395	CHO	0.250	1.100	10	0.429
C17H26O5S1	341.1428	CHOS	0.294	1.529	5	0.111
C17H26O7	341.1607	CHO	0.412	1.529	5	0.111
C21H26O4	341.1759	CHO	0.190	1.238	9	0.368
C18H30O6	341.1970	CHO	0.333	1.667	4	0.067
C15H21N1O8	342.1196	CHNO	0.533	1.400	6	0.150
C19H21N1O5	342.1348	CHNO	0.263	1.105	10	0.452
C18H16O7	343.0823	CHO	0.389	0.889	11	0.517
C15H20O7S1	343.0856	CHOS	0.467	1.333	6	0.143
C15H20O9	343.1035	CHO	0.600	1.333	6	0.143
C19H20O6	343.1187	CHO	0.316	1.053	10	0.438
C16H24O6S1	343.1220	CHOS	0.375	1.500	5	0.083
C16H24O8	343.1398	CHO	0.500	1.500	5	0.083
C20H24O5	343.1551	CHO	0.250	1.200	9	0.371
C17H28O7	343.1762	CHO	0.412	1.647	4	0.037
C21H28O4	343.1915	CHO	0.190	1.333	8	0.316
C18H19N1O6	344.1139	CHNO	0.333	1.056	10	0.464
C19H23N1O5	344.1503	CHNO	0.263	1.211	9	0.387
C14H18O8S1	345.0650	CHOS	0.571	1.286	6	0.111
C18H18O7	345.0981	CHO	0.389	1.000	10	0.448
C15H22O7S1	345.1014	CHOS	0.467	1.467	5	0.048
C17H18N2O6	345.1093	CHNO	0.353	1.059	10	0.500
C19H22O6	345.1344	CHO	0.316	1.158	9	0.375
C16H26O8	345.1555	CHO	0.500	1.625	4	0.000
C20H26O5	345.1708	CHO	0.250	1.300	8	0.314
C17H17N1O7	346.0933	CHNO	0.412	1.000	10	0.480
C18H21N1O6	346.1297	CHNO	0.333	1.167	9	0.393

C19H25N1O5	346.1661	CHNO	0.263	1.316	8	0.323
C17H16O8	347.0772	CHO	0.471	0.941	10	0.462
C14H20O8S1	347.0807	CHOS	0.571	1.429	5	0.000
C18H20O7	347.1136	CHO	0.389	1.111	9	0.379
C15H24O7S1	347.1169	CHOS	0.467	1.600	4	-0.048
C15H24O9	347.1346	CHO	0.600	1.600	4	-0.048
C19H24O6	347.1499	CHO	0.316	1.263	8	0.313
C16H28O8	347.1711	CHO	0.500	1.750	3	-0.083
C20H28O5	347.1864	CHO	0.250	1.400	7	0.257
C17H19N1O7	348.1089	CHNO	0.412	1.118	9	0.400
C18H23N1O6	348.1453	CHNO	0.333	1.278	8	0.321
C17H18O8	349.0930	CHO	0.471	1.059	9	0.385
C14H22O8S1	349.0964	CHOS	0.571	1.571	4	-0.111
C18H22O7	349.1293	CHO	0.389	1.222	8	0.310
C19H26O6	349.1657	CHO	0.316	1.368	7	0.250
C20H30O5	349.2021	CHO	0.250	1.500	6	0.200
C16H17N1O8	350.0882	CHNO	0.500	1.063	9	0.409
C17H21N1O7	350.1246	CHNO	0.412	1.235	8	0.320
C18H25N1O6	350.1610	CHNO	0.333	1.389	7	0.250
C16H16O7S1	351.0542	CHOS	0.438	1.000	9	0.391
C16H16O9	351.0720	CHO	0.563	1.000	9	0.391
C17H20O6S1	351.0907	CHOS	0.353	1.176	8	0.308
C17H20O8	351.1085	CHO	0.471	1.176	8	0.308
C18H24O7	351.1449	CHO	0.389	1.333	7	0.241
C22H24O4	351.1601	CHO	0.182	1.091	11	0.450
C19H28O6	351.1813	CHO	0.316	1.474	6	0.188
C20H32O5	351.2177	CHO	0.250	1.600	5	0.143
C16H19N1O8	352.1038	CHNO	0.500	1.188	8	0.318
C17H23N1O7	352.1402	CHNO	0.412	1.353	7	0.240
C18H27N1O6	352.1766	CHNO	0.333	1.500	6	0.179
C16H18O7S1	353.0701	CHOS	0.438	1.125	8	0.304
C16H18O9	353.0879	CHO	0.563	1.125	8	0.304
C17H22O8	353.1243	CHO	0.471	1.294	7	0.231
C21H22O5	353.1396	CHO	0.238	1.048	11	0.459
C18H26O7	353.1607	CHO	0.389	1.444	6	0.172
C22H26O4	353.1760	CHO	0.182	1.182	10	0.400
C19H30O6	353.1971	CHO	0.316	1.579	5	0.125
C18H17N3O5	354.1098	CHNO	0.278	0.944	12	0.640
C16H21N1O8	354.1195	CHNO	0.500	1.313	7	0.227
C17H25N1O7	354.1558	CHNO	0.412	1.471	6	0.160
C16H20O7S1	355.0856	CHOS	0.438	1.250	7	0.217
C16H20O9	355.1034	CHO	0.563	1.250	7	0.217
C20H20O6	355.1187	CHO	0.300	1.000	11	0.471
C17H24O8	355.1398	CHO	0.471	1.412	6	0.154
C21H24O5	355.1550	CHO	0.238	1.143	10	0.405
C18H28O7	355.1762	CHO	0.389	1.556	5	0.103
C22H28O4	355.1915	CHO	0.182	1.273	9	0.350
C19H32O6	355.2125	CHO	0.316	1.684	4	0.063
C16H23N1O8	356.1352	CHNO	0.500	1.438	6	0.136
C20H23N1O5	356.1504	CHNO	0.250	1.150	10	0.424

C15H18O8S1	357.0649	CHOS	0.533	1.200	7	0.200
C15H18O10	357.0833	CHO	0.667	1.200	7	0.200
C19H18O7	357.0980	CHO	0.368	0.947	11	0.484
C16H22O7S1	357.1014	CHOS	0.438	1.375	6	0.130
C16H22O9	357.1191	CHO	0.563	1.375	6	0.130
C20H22O6	357.1344	CHO	0.300	1.100	10	0.412
C17H26O8	357.1555	CHO	0.471	1.529	5	0.077
C21H26O5	357.1708	CHO	0.238	1.238	9	0.351
C18H30O7	357.1919	CHO	0.389	1.667	4	0.034
C17H17N3O6	358.1046	CHNO	0.353	1.000	11	0.591
C19H21N1O6	358.1296	CHNO	0.316	1.105	10	0.433
C20H25N1O5	358.1659	CHNO	0.250	1.250	9	0.364
C15H20O8S1	359.0807	CHOS	0.533	1.333	6	0.100
C19H20O7	359.1137	CHO	0.368	1.053	10	0.419
C16H24O7S1	359.1171	CHOS	0.438	1.500	5	0.043
C18H20N2O6	359.1250	CHNO	0.333	1.111	10	0.462
C16H24O9	359.1348	CHO	0.563	1.500	5	0.043
C20H24O6	359.1501	CHO	0.300	1.200	9	0.353
C17H28O8	359.1713	CHO	0.471	1.647	4	0.000
C21H28O5	359.1864	CHO	0.238	1.333	8	0.297
C18H19N1O7	360.1090	CHNO	0.389	1.056	10	0.444
C19H23N1O6	360.1454	CHNO	0.316	1.211	9	0.367
C18H18O8	361.0928	CHO	0.444	1.000	10	0.429
C15H22O8S1	361.0963	CHOS	0.533	1.467	5	0.000
C19H22O7	361.1292	CHO	0.368	1.158	9	0.355
C18H22N2O6	361.1405	CHNO	0.333	1.222	9	0.385
C16H26O9	361.1502	CHO	0.563	1.625	4	-0.043
C20H26O6	361.1656	CHO	0.300	1.300	8	0.294
C21H30O5	361.2020	CHO	0.238	1.429	7	0.243
C18H21N1O7	362.1246	CHNO	0.389	1.167	9	0.370
C17H21N3O6	362.1356	CHNO	0.353	1.235	9	0.409
C19H25N1O6	362.1609	CHNO	0.316	1.316	8	0.300
C17H16O9	363.0723	CHO	0.529	0.941	10	0.440
C14H20O9S1	363.0754	CHOS	0.643	1.429	5	-0.059
C18H20O6S1	363.0909	CHOS	0.333	1.111	9	0.357
C18H20O8	363.1086	CHO	0.444	1.111	9	0.357
C15H24O8S1	363.1120	CHOS	0.533	1.600	4	-0.100
C19H24O7	363.1450	CHO	0.368	1.263	8	0.290
C18H24N2O6	363.1563	CHNO	0.333	1.333	8	0.308
C20H28O6	363.1814	CHO	0.300	1.400	7	0.235
C21H32O5	363.2178	CHO	0.238	1.524	6	0.189
C17H19N1O8	364.1038	CHNO	0.471	1.118	9	0.375
C18H23N1O7	364.1402	CHNO	0.389	1.278	8	0.296
C19H27N1O6	364.1765	CHNO	0.316	1.421	7	0.233
C20H14O7	365.0661	CHO	0.350	0.700	14	0.636
C17H18O7S1	365.0706	CHOS	0.412	1.059	9	0.360
C17H18O9	365.0879	CHO	0.529	1.059	9	0.360
C14H22O9S1	365.0912	CHOS	0.643	1.571	4	-0.176
C18H22O8	365.1242	CHO	0.444	1.222	8	0.286
C19H26O7	365.1606	CHO	0.368	1.368	7	0.226

C20H30O6	365.1970	CHO	0.300	1.500	6	0.176
C17H21N1O8	366.1196	CHNO	0.471	1.235	8	0.292
C18H25N1O7	366.1560	CHNO	0.389	1.389	7	0.222
C16H16O8S1	367.0494	CHOS	0.500	1.000	9	0.364
C17H20O7S1	367.0857	CHOS	0.412	1.176	8	0.280
C17H20O9	367.1034	CHO	0.529	1.176	8	0.280
C21H20O6	367.1186	CHO	0.286	0.952	12	0.500
C18H24O8	367.1398	CHO	0.444	1.333	7	0.214
C22H24O5	367.1551	CHO	0.227	1.091	11	0.436
C19H28O5S1	367.1583	CHOS	0.263	1.474	6	0.161
C19H28O7	367.1762	CHO	0.368	1.474	6	0.161
C23H28O4	367.1914	CHO	0.174	1.217	10	0.381
C20H32O6	367.2126	CHO	0.300	1.600	5	0.118
C19H19N3O5	368.1255	CHNO	0.263	1.000	12	0.593
C17H23N1O8	368.1352	CHNO	0.471	1.353	7	0.208
C18H27N1O7	368.1716	CHNO	0.389	1.500	6	0.148
C16H18O8S1	369.0650	CHOS	0.500	1.125	8	0.273
C16H18O10	369.0827	CHO	0.625	1.125	8	0.273
C17H22O7S1	369.1014	CHOS	0.412	1.294	7	0.200
C17H22O9	369.1192	CHO	0.529	1.294	7	0.200
C21H22O6	369.1344	CHO	0.286	1.048	11	0.444
C18H26O8	369.1556	CHO	0.444	1.444	6	0.143
C22H26O5	369.1708	CHO	0.227	1.182	10	0.385
C19H30O7	369.1920	CHO	0.368	1.579	5	0.097
C18H17N3O6	370.1047	CHNO	0.333	0.944	12	0.625
C16H21N1O9	370.1144	CHNO	0.563	1.313	7	0.190
C17H25N1O8	370.1507	CHNO	0.471	1.471	6	0.125
C16H20O8S1	371.0807	CHOS	0.500	1.250	7	0.182
C16H20O10	371.0986	CHO	0.625	1.250	7	0.182
C20H20O7	371.1137	CHO	0.350	1.000	11	0.455
C17H24O7S1	371.1170	CHOS	0.412	1.412	6	0.120
C17H24O9	371.1348	CHO	0.529	1.412	6	0.120
C21H24O6	371.1501	CHO	0.286	1.143	10	0.389
C18H28O6S1	371.1531	CHOS	0.333	1.556	5	0.071
C18H28O8	371.1712	CHO	0.444	1.556	5	0.071
C22H28O5	371.1865	CHO	0.227	1.273	9	0.333
C19H32O7	371.2076	CHO	0.368	1.684	4	0.032
C23H32O4	371.2227	CHO	0.174	1.391	8	0.286
C18H19N3O6	372.1202	CHNO	0.333	1.056	11	0.542
C16H23N1O9	372.1301	CHNO	0.563	1.438	6	0.095
C20H23N1O6	372.1453	CHNO	0.300	1.150	10	0.406
C15H18O9S1	373.0599	CHOS	0.600	1.200	7	0.158
C19H18O8	373.0929	CHO	0.421	0.947	11	0.467
C16H22O8S1	373.0963	CHOS	0.500	1.375	6	0.091
C20H22O7	373.1292	CHO	0.350	1.100	10	0.394
C17H26O7S1	373.1326	CHOS	0.412	1.529	5	0.040
C17H26O9	373.1504	CHO	0.529	1.529	5	0.040
C21H26O6	373.1656	CHO	0.286	1.238	9	0.333
C18H30O8	373.1867	CHO	0.444	1.667	4	0.000
C22H30O5	373.2020	CHO	0.227	1.364	8	0.282

C19H21N1O7	374.1246	CHNO	0.368	1.105	10	0.414
C20H25N1O6	374.1610	CHNO	0.300	1.250	9	0.344
C15H20O9S1	375.0754	CHOS	0.600	1.333	6	0.053
C19H20O8	375.1085	CHO	0.421	1.053	10	0.400
C16H24O8S1	375.1119	CHOS	0.500	1.500	5	0.000
C20H24O7	375.1449	CHO	0.350	1.200	9	0.333
C17H28O7S1	375.1479	CHOS	0.412	1.647	4	-0.040
C21H28O6	375.1813	CHO	0.286	1.333	8	0.278
C18H19N1O8	376.1038	CHNO	0.444	1.056	10	0.423
C19H23N1O7	376.1402	CHNO	0.368	1.211	9	0.345
C18H23N3O6	376.1515	CHNO	0.333	1.278	9	0.375
C20H27N1O6	376.1765	CHNO	0.300	1.350	8	0.281
C18H18O7S1	377.0702	CHOS	0.389	1.000	10	0.407
C18H18O9	377.0879	CHO	0.500	1.000	10	0.407
C19H22O8	377.1243	CHO	0.421	1.158	9	0.333
C16H26O8S1	377.1273	CHOS	0.500	1.625	4	-0.091
C18H22N2O7	377.1356	CHNO	0.389	1.222	9	0.360
C20H26O7	377.1607	CHO	0.350	1.300	8	0.273
C21H30O6	377.1971	CHO	0.286	1.429	7	0.222
C18H21N1O8	378.1194	CHNO	0.444	1.167	9	0.346
C17H21N3O7	378.1306	CHNO	0.412	1.235	9	0.381
C19H25N1O7	378.1558	CHNO	0.368	1.316	8	0.276
C20H29N1O6	378.1922	CHNO	0.300	1.450	7	0.219
C18H20O7S1	379.0858	CHOS	0.389	1.111	9	0.333
C18H20O9	379.1035	CHO	0.500	1.111	9	0.333
C19H24O8	379.1399	CHO	0.421	1.263	8	0.267
C16H28O8S1	379.1429	CHOS	0.500	1.750	3	-0.182
C18H24N2O7	379.1512	CHNO	0.389	1.333	8	0.280
C20H28O7	379.1763	CHO	0.350	1.400	7	0.212
C21H32O6	379.2126	CHO	0.286	1.524	6	0.167
C18H23N1O8	380.1352	CHNO	0.444	1.278	8	0.269
C19H27N1O7	380.1715	CHNO	0.368	1.421	7	0.207
C17H18O8S1	381.0649	CHOS	0.471	1.059	9	0.333
C17H18O10	381.0826	CHO	0.588	1.059	9	0.333
C21H18O7	381.0984	CHO	0.333	0.857	13	0.543
C18H22O9	381.1191	CHO	0.500	1.222	8	0.259
C22H22O6	381.1347	CHO	0.273	1.000	12	0.474
C19H26O6S1	381.1377	CHOS	0.316	1.368	7	0.200
C19H26O8	381.1554	CHO	0.421	1.368	7	0.200
C23H26O5	381.1707	CHO	0.217	1.130	11	0.415
C20H30O7	381.1918	CHO	0.350	1.500	6	0.152
C21H34O6	381.2282	CHO	0.286	1.619	5	0.111
C19H17N3O6	382.1048	CHNO	0.316	0.895	13	0.654
C17H21N1O9	382.1145	CHNO	0.529	1.235	8	0.261
C21H21N1O6	382.1298	CHNO	0.286	1.000	12	0.500
C20H21N3O5	382.1412	CHNO	0.250	1.050	12	0.552
C18H25N1O8	382.1509	CHNO	0.444	1.389	7	0.192
C19H29N1O7	382.1871	CHNO	0.368	1.526	6	0.138
C17H20O8S1	383.0806	CHOS	0.471	1.176	8	0.250
C18H24O7S1	383.1169	CHOS	0.389	1.333	7	0.185

C18H24O9	383.1347	CHO	0.500	1.333	7	0.185
C22H24O6	383.1500	CHO	0.273	1.091	11	0.421
C19H28O6S1	383.1533	CHOS	0.316	1.474	6	0.133
C19H28O8	383.1711	CHO	0.421	1.474	6	0.133
C23H28O5	383.1864	CHO	0.217	1.217	10	0.366
C20H32O7	383.2075	CHO	0.350	1.600	5	0.091
C18H15N3O7	384.0840	CHNO	0.389	0.833	13	0.696
C20H19N1O7	384.1088	CHNO	0.350	0.950	12	0.516
C19H19N3O6	384.1204	CHNO	0.316	1.000	12	0.577
C17H23N1O9	384.1301	CHNO	0.529	1.353	7	0.174
C21H23N1O6	384.1454	CHNO	0.286	1.095	11	0.441
C20H23N3O5	384.1569	CHNO	0.250	1.150	11	0.483
C18H27N1O8	384.1664	CHNO	0.444	1.500	6	0.115
C20H18O8	385.0931	CHO	0.400	0.900	12	0.500
C17H22O8S1	385.0963	CHOS	0.471	1.294	7	0.167
C17H22O10	385.1140	CHO	0.588	1.294	7	0.167
C21H22O7	385.1293	CHO	0.333	1.048	11	0.429
C18H26O7S1	385.1327	CHOS	0.389	1.444	6	0.111
C18H26O9	385.1505	CHO	0.500	1.444	6	0.111
C22H26O6	385.1657	CHO	0.273	1.182	10	0.368
C19H30O8	385.1869	CHO	0.421	1.579	5	0.067
C23H30O5	385.2021	CHO	0.217	1.304	9	0.317
C18H17N3O7	386.0996	CHNO	0.389	0.944	12	0.609
C20H21N1O7	386.1245	CHNO	0.350	1.050	11	0.452
C19H21N3O6	386.1357	CHNO	0.316	1.105	11	0.500
C21H25N1O6	386.1609	CHNO	0.286	1.190	10	0.382
C16H20O9S1	387.0757	CHOS	0.563	1.250	7	0.143
C20H20O8	387.1086	CHO	0.400	1.000	11	0.438
C17H24O8S1	387.1120	CHOS	0.471	1.412	6	0.083
C17H24O10	387.1298	CHO	0.588	1.412	6	0.083
C21H24O7	387.1451	CHO	0.333	1.143	10	0.371
C18H28O7S1	387.1484	CHOS	0.389	1.556	5	0.037
C18H28O9	387.1663	CHO	0.500	1.556	5	0.037
C22H28O6	387.1814	CHO	0.273	1.273	9	0.316
C19H32O8	387.2027	CHO	0.421	1.684	4	0.000
C20H23N1O7	388.1400	CHNO	0.350	1.150	10	0.387
C19H23N3O6	388.1514	CHNO	0.316	1.211	10	0.423
C21H27N1O6	388.1765	CHNO	0.286	1.286	9	0.324
C19H18O9	389.0880	CHO	0.474	0.947	11	0.448
C16H22O9S1	389.0913	CHOS	0.563	1.375	6	0.048
C20H22O8	389.1243	CHO	0.400	1.100	10	0.375
C17H26O8S1	389.1276	CHOS	0.471	1.529	5	0.000
C21H26O7	389.1606	CHO	0.333	1.238	9	0.314
C22H30O6	389.1970	CHO	0.273	1.364	8	0.263
C19H21N1O8	390.1195	CHNO	0.421	1.105	10	0.393
C18H21N3O7	390.1308	CHNO	0.389	1.167	10	0.435
C20H25N1O7	390.1559	CHNO	0.350	1.250	9	0.323
C19H25N3O6	390.1672	CHNO	0.316	1.316	9	0.346
C21H29N1O6	390.1922	CHNO	0.286	1.381	8	0.265
C19H20O9	391.1035	CHO	0.474	1.053	10	0.379

C16H24O9S1	391.1067	CHOS	0.563	1.500	5	-0.048
C20H24O8	391.1398	CHO	0.400	1.200	9	0.313
C21H28O7	391.1762	CHO	0.333	1.333	8	0.257
C22H32O6	391.2126	CHO	0.273	1.455	7	0.211
C19H23N1O8	392.1352	CHNO	0.421	1.211	9	0.321
C20H27N1O7	392.1716	CHNO	0.350	1.350	8	0.258
C18H18O8S1	393.0649	CHOS	0.444	1.000	10	0.385
C18H18O10	393.0827	CHO	0.556	1.000	10	0.385
C19H22O9	393.1190	CHO	0.474	1.158	9	0.310
C20H26O6S1	393.1376	CHOS	0.300	1.300	8	0.250
C20H26O8	393.1554	CHO	0.400	1.300	8	0.250
C19H26N2O7	393.1666	CHNO	0.368	1.368	8	0.259
C21H30O7	393.1918	CHO	0.333	1.429	7	0.200
C20H17N3O6	394.1048	CHNO	0.300	0.850	14	0.679
C19H25N1O8	394.1509	CHNO	0.421	1.316	8	0.250
C20H29N1O7	394.1872	CHNO	0.350	1.450	7	0.194
C18H20O8S1	395.0805	CHOS	0.444	1.111	9	0.308
C18H20O10	395.0983	CHO	0.556	1.111	9	0.308
C19H24O9	395.1347	CHO	0.474	1.263	8	0.241
C23H24O6	395.1500	CHO	0.261	1.043	12	0.450
C20H28O8	395.1711	CHO	0.400	1.400	7	0.188
C24H28O5	395.1864	CHO	0.208	1.167	11	0.395
C21H32O7	395.2075	CHO	0.333	1.524	6	0.143
C20H19N3O6	396.1204	CHNO	0.300	0.950	13	0.607
C18H23N1O9	396.1299	CHNO	0.500	1.278	8	0.240
C21H23N3O5	396.1568	CHNO	0.238	1.095	12	0.516
C19H27N1O8	396.1665	CHNO	0.421	1.421	7	0.179
C18H22O8S1	397.0963	CHOS	0.444	1.222	8	0.231
C18H22O10	397.1140	CHO	0.556	1.222	8	0.231
C22H22O7	397.1293	CHO	0.318	1.000	12	0.459
C19H26O7S1	397.1327	CHOS	0.368	1.368	7	0.172
C19H26O9	397.1504	CHO	0.474	1.368	7	0.172
C23H26O6	397.1657	CHO	0.261	1.130	11	0.400
C20H30O6S1	397.1688	CHOS	0.300	1.500	6	0.125
C20H30O8	397.1868	CHO	0.400	1.500	6	0.125
C24H30O5	397.2021	CHO	0.208	1.250	10	0.349
C21H34O7	397.2233	CHO	0.333	1.619	5	0.086
C19H17N3O7	398.0996	CHNO	0.368	0.895	13	0.640
C20H21N3O6	398.1360	CHNO	0.300	1.050	12	0.536
C18H25N1O9	398.1457	CHNO	0.500	1.389	7	0.160
C22H25N1O6	398.1608	CHNO	0.273	1.136	11	0.417
C17H20O9S1	399.0756	CHOS	0.529	1.176	8	0.217
C21H20O8	399.1087	CHO	0.381	0.952	12	0.471
C18H24O8S1	399.1120	CHOS	0.444	1.333	7	0.154
C18H24O10	399.1298	CHO	0.556	1.333	7	0.154
C22H24O7	399.1450	CHO	0.318	1.091	11	0.405
C19H28O7S1	399.1483	CHOS	0.368	1.474	6	0.103
C19H28O9	399.1662	CHO	0.474	1.474	6	0.103
C23H28O6	399.1814	CHO	0.261	1.217	10	0.350
C20H32O8	399.2025	CHO	0.400	1.600	5	0.063

C24H32O5	399.2178	CHO	0.208	1.333	9	0.302
C19H19N3O7	400.1152	CHNO	0.368	1.000	12	0.560
C20H23N3O6	400.1517	CHNO	0.300	1.150	11	0.464
C18H27N1O9	400.1613	CHNO	0.500	1.500	6	0.080
C22H27N1O6	400.1765	CHNO	0.273	1.227	10	0.361
C20H18O9	401.0883	CHO	0.450	0.900	12	0.484
C17H22O9S1	401.0913	CHOS	0.529	1.294	7	0.130
C17H22O11	401.1090	CHO	0.647	1.294	7	0.130
C21H22O8	401.1243	CHO	0.381	1.048	11	0.412
C18H26O8S1	401.1277	CHOS	0.444	1.444	6	0.077
C18H26O10	401.1454	CHO	0.556	1.444	6	0.077
C22H26O7	401.1607	CHO	0.318	1.182	10	0.351
C19H30O7S1	401.1640	CHOS	0.368	1.579	5	0.034
C19H30O9	401.1817	CHO	0.474	1.579	5	0.034
C23H30O6	401.1971	CHO	0.261	1.304	9	0.300
C18H17N3O8	402.0945	CHNO	0.444	0.944	12	0.591
C21H25N1O7	402.1558	CHNO	0.333	1.190	10	0.364
C20H25N3O6	402.1671	CHNO	0.300	1.250	10	0.393
C20H20O9	403.1036	CHO	0.450	1.000	11	0.419
C17H24O9S1	403.1069	CHOS	0.529	1.412	6	0.043
C17H24O11	403.1245	CHO	0.647	1.412	6	0.043
C21H24O8	403.1400	CHO	0.381	1.143	10	0.353
C18H28O8S1	403.1433	CHOS	0.444	1.556	5	0.000
C22H28O7	403.1764	CHO	0.318	1.273	9	0.297
C23H32O6	403.2127	CHO	0.261	1.391	8	0.250
C20H23N1O8	404.1353	CHNO	0.400	1.150	10	0.367
C19H23N3O7	404.1463	CHNO	0.368	1.211	10	0.400
C21H27N1O7	404.1714	CHNO	0.333	1.286	9	0.303
C20H22O9	405.1192	CHO	0.450	1.100	10	0.355
C21H26O8	405.1556	CHO	0.381	1.238	9	0.294
C20H26N2O7	405.1668	CHNO	0.350	1.300	9	0.310
C22H30O7	405.1920	CHO	0.318	1.364	8	0.243
C23H34O6	405.2282	CHO	0.261	1.478	7	0.200
C20H25N1O8	406.1507	CHNO	0.400	1.250	9	0.300
C19H25N3O7	406.1619	CHNO	0.368	1.316	9	0.320
C21H29N1O7	406.1871	CHNO	0.333	1.381	8	0.242
C19H20O8S1	407.0807	CHOS	0.421	1.053	10	0.357
C19H20O10	407.0984	CHO	0.526	1.053	10	0.357
C20H24O9	407.1348	CHO	0.450	1.200	9	0.290
C21H28O8	407.1712	CHO	0.381	1.333	8	0.235
C22H32O7	407.2076	CHO	0.318	1.455	7	0.189
C20H17N3O7	410.0997	CHNO	0.350	0.850	14	0.667
C21H21N3O6	410.1360	CHNO	0.286	1.000	13	0.567
C19H25N1O9	410.1458	CHNO	0.474	1.316	8	0.222
C20H29N1O8	410.1823	CHNO	0.400	1.450	7	0.167
C18H20O9S1	411.0756	CHOS	0.500	1.111	9	0.280
C22H20O8	411.1089	CHO	0.364	0.909	13	0.500
C19H24O8S1	411.1119	CHOS	0.421	1.263	8	0.214
C19H24O10	411.1297	CHO	0.526	1.263	8	0.214
C23H24O7	411.1450	CHO	0.304	1.043	12	0.436

C20H28O7S1	411.1483	CHOS	0.350	1.400	7	0.161
C20H28O9	411.1660	CHO	0.450	1.400	7	0.161
C24H28O6	411.1813	CHO	0.250	1.167	11	0.381
C21H32O6S1	411.1839	CHOS	0.286	1.524	6	0.118
C21H32O8	411.2024	CHO	0.381	1.524	6	0.118
C20H19N3O7	412.1154	CHNO	0.350	0.950	13	0.593
C22H23N1O7	412.1402	CHNO	0.318	1.045	12	0.457
C21H23N3O6	412.1518	CHNO	0.286	1.095	12	0.500
C19H27N1O9	412.1615	CHNO	0.474	1.421	7	0.148
C21H18O9	413.0885	CHO	0.429	0.857	13	0.515
C18H22O9S1	413.0911	CHOS	0.500	1.222	8	0.200
C22H22O8	413.1243	CHO	0.364	1.000	12	0.444
C19H26O8S1	413.1275	CHOS	0.421	1.368	7	0.143
C19H26O10	413.1453	CHO	0.526	1.368	7	0.143
C23H26O7	413.1605	CHO	0.304	1.130	11	0.385
C20H30O7S1	413.1637	CHOS	0.350	1.500	6	0.097
C20H30O9	413.1817	CHO	0.450	1.500	6	0.097
C24H30O6	413.1970	CHO	0.250	1.250	10	0.333
C21H34O8	413.2181	CHO	0.381	1.619	5	0.059
C19H17N3O8	414.0946	CHNO	0.421	0.895	13	0.625
C20H21N3O7	414.1310	CHNO	0.350	1.050	12	0.519
C22H25N1O7	414.1558	CHNO	0.318	1.136	11	0.400
C21H25N3O6	414.1674	CHNO	0.286	1.190	11	0.433
C21H20O9	415.1036	CHO	0.429	0.952	12	0.455
C18H24O9S1	415.1068	CHOS	0.500	1.333	7	0.120
C18H24O11	415.1244	CHO	0.611	1.333	7	0.120
C22H24O8	415.1398	CHO	0.364	1.091	11	0.389
C19H28O8S1	415.1432	CHOS	0.421	1.474	6	0.071
C19H28O10	415.1609	CHO	0.526	1.474	6	0.071
C23H28O7	415.1762	CHO	0.304	1.217	10	0.333
C20H32O9	415.1972	CHO	0.450	1.600	5	0.032
C24H32O6	415.2126	CHO	0.250	1.333	9	0.286
C19H19N3O8	416.1105	CHNO	0.421	1.000	12	0.542
C21H23N1O8	416.1350	CHNO	0.381	1.095	11	0.406
C20H23N3O7	416.1466	CHNO	0.350	1.150	11	0.444
C22H27N1O7	416.1716	CHNO	0.318	1.227	10	0.343
C20H18O10	417.0828	CHO	0.500	0.900	12	0.467
C17H22O10S1	417.0860	CHOS	0.588	1.294	7	0.091
C21H22O9	417.1191	CHO	0.429	1.048	11	0.394
C18H26O9S1	417.1224	CHOS	0.500	1.444	6	0.040
C18H26O11	417.1398	CHO	0.611	1.444	6	0.040
C22H26O8	417.1554	CHO	0.364	1.182	10	0.333
C19H30O8S1	417.1588	CHOS	0.421	1.579	5	0.000
C23H30O7	417.1918	CHO	0.304	1.304	9	0.282
C20H34O9	417.2131	CHO	0.450	1.700	4	-0.032
C24H34O6	417.2283	CHO	0.250	1.417	8	0.238
C21H25N1O8	418.1508	CHNO	0.381	1.190	10	0.344
C20H25N3O7	418.1623	CHNO	0.350	1.250	10	0.370
C22H29N1O7	418.1873	CHNO	0.318	1.318	9	0.286
C20H20O10	419.0984	CHO	0.500	1.000	11	0.400

C17H24O10S1	419.1015	CHOS	0.588	1.412	6	0.000
C21H24O9	419.1347	CHO	0.429	1.143	10	0.333
C18H28O9S1	419.1374	CHOS	0.500	1.556	5	-0.040
C22H28O8	419.1710	CHO	0.364	1.273	9	0.278
C19H32O8S1	419.1739	CHOS	0.421	1.684	4	-0.071
C23H32O7	419.2075	CHO	0.304	1.391	8	0.231
C21H27N1O8	420.1666	CHNO	0.381	1.286	9	0.281
C20H22O8S1	421.0962	CHOS	0.400	1.100	10	0.333
C20H22O10	421.1139	CHO	0.500	1.100	10	0.333
C21H26O7S1	421.1326	CHOS	0.333	1.238	9	0.273
C21H26O9	421.1503	CHO	0.429	1.238	9	0.273
C22H30O8	421.1867	CHO	0.364	1.364	8	0.222
C23H34O7	421.2232	CHO	0.304	1.478	7	0.179
C22H21N3O6	422.1362	CHNO	0.273	0.955	14	0.594
C21H29N1O8	422.1822	CHNO	0.381	1.381	8	0.219
C19H20O9S1	423.0754	CHOS	0.474	1.053	10	0.333
C19H20O11	423.0930	CHO	0.579	1.053	10	0.333
C20H24O8S1	423.1119	CHOS	0.400	1.200	9	0.267
C20H24O10	423.1296	CHO	0.500	1.200	9	0.267
C24H24O7	423.1450	CHO	0.292	1.000	13	0.463
C21H28O7S1	423.1482	CHOS	0.333	1.333	8	0.212
C21H28O9	423.1660	CHO	0.429	1.333	8	0.212
C22H32O8	423.2024	CHO	0.364	1.455	7	0.167
C23H36O7	423.2388	CHO	0.304	1.565	6	0.128
C21H19N3O7	424.1154	CHNO	0.333	0.905	14	0.621
C20H27N1O9	424.1614	CHNO	0.450	1.350	8	0.207
C19H22O9S1	425.0912	CHOS	0.474	1.158	9	0.259
C19H22O11	425.1089	CHO	0.579	1.158	9	0.259
C23H22O8	425.1243	CHO	0.348	0.957	13	0.474
C20H26O8S1	425.1275	CHOS	0.400	1.300	8	0.200
C20H26O10	425.1453	CHO	0.500	1.300	8	0.200
C24H26O7	425.1606	CHO	0.292	1.083	12	0.415
C21H30O7S1	425.1639	CHOS	0.333	1.429	7	0.152
C21H30O9	425.1816	CHO	0.429	1.429	7	0.152
C25H30O6	425.1969	CHO	0.240	1.200	11	0.364
C22H34O8	425.2180	CHO	0.364	1.545	6	0.111
C21H21N3O7	426.1310	CHNO	0.333	1.000	13	0.552
C23H25N1O7	426.1560	CHNO	0.304	1.087	12	0.432
C22H25N3O6	426.1674	CHNO	0.273	1.136	12	0.469
C18H20O10S1	427.0703	CHOS	0.556	1.111	9	0.250
C19H24O9S1	427.1068	CHOS	0.474	1.263	8	0.185
C19H24O11	427.1245	CHO	0.579	1.263	8	0.185
C23H24O8	427.1399	CHO	0.348	1.043	12	0.421
C20H28O8S1	427.1432	CHOS	0.400	1.400	7	0.133
C20H28O10	427.1609	CHO	0.500	1.400	7	0.133
C24H28O7	427.1762	CHO	0.292	1.167	11	0.366
C21H32O7S1	427.1794	CHOS	0.333	1.524	6	0.091
C21H32O9	427.1973	CHO	0.429	1.524	6	0.091
C25H32O6	427.2125	CHO	0.240	1.280	10	0.318
C22H36O8	427.2337	CHO	0.364	1.636	5	0.056

C20H19N3O8	428.1103	CHNO	0.400	0.950	13	0.577
C21H23N3O7	428.1467	CHNO	0.333	1.095	12	0.483
C18H22O10S1	429.0861	CHOS	0.556	1.222	8	0.167
C22H22O9	429.1191	CHO	0.409	1.000	12	0.429
C19H26O9S1	429.1225	CHOS	0.474	1.368	7	0.111
C19H26O11	429.1402	CHO	0.579	1.368	7	0.111
C23H26O8	429.1554	CHO	0.348	1.130	11	0.368
C20H30O8S1	429.1588	CHOS	0.400	1.500	6	0.067
C20H30O10	429.1765	CHO	0.500	1.500	6	0.067
C24H30O7	429.1918	CHO	0.292	1.250	10	0.317
C21H34O9	429.2131	CHO	0.429	1.619	5	0.030
C25H34O6	429.2283	CHO	0.240	1.360	9	0.273
C20H21N3O8	430.1260	CHNO	0.400	1.050	12	0.500
C22H25N1O8	430.1509	CHNO	0.364	1.136	11	0.382
C21H25N3O7	430.1622	CHNO	0.333	1.190	11	0.414
C22H29N3O6	430.1986	CHNO	0.273	1.318	10	0.344
C21H20O10	431.0984	CHO	0.476	0.952	12	0.438
C18H24O10S1	431.1019	CHOS	0.556	1.333	7	0.083
C22H24O9	431.1348	CHO	0.409	1.091	11	0.371
C19H28O9S1	431.1382	CHOS	0.474	1.474	6	0.037
C23H28O8	431.1711	CHO	0.348	1.217	10	0.316
C20H32O8S1	431.1745	CHOS	0.400	1.600	5	0.000
C24H32O7	431.2075	CHO	0.292	1.333	9	0.268
C20H23N3O8	432.1413	CHNO	0.400	1.150	11	0.423
C22H27N1O8	432.1665	CHNO	0.364	1.227	10	0.324
C21H27N3O7	432.1780	CHNO	0.333	1.286	10	0.345
C21H22O10	433.1141	CHO	0.476	1.048	11	0.375
C18H26O10S1	433.1174	CHOS	0.556	1.444	6	0.000
C22H26O9	433.1504	CHO	0.409	1.182	10	0.314
C23H30O8	433.1868	CHO	0.348	1.304	9	0.263
C24H34O7	433.2232	CHO	0.292	1.417	8	0.220
C21H25N1O9	434.1457	CHNO	0.429	1.190	10	0.323
C22H29N1O8	434.1821	CHNO	0.364	1.318	9	0.265
C20H20O9S1	435.0756	CHOS	0.450	1.000	11	0.379
C21H24O10	435.1298	CHO	0.476	1.143	10	0.313
C18H28O10S1	435.1329	CHOS	0.556	1.556	5	-0.083
C22H28O7S1	435.1484	CHOS	0.318	1.273	9	0.257
C22H28O9	435.1661	CHO	0.409	1.273	9	0.257
C23H32O8	435.2025	CHO	0.348	1.391	8	0.211
C24H36O7	435.2389	CHO	0.292	1.500	7	0.171
C20H22O9S1	437.0913	CHOS	0.450	1.100	10	0.310
C20H22O11	437.1090	CHO	0.550	1.100	10	0.310
C21H26O10	437.1455	CHO	0.476	1.238	9	0.250
C22H30O9	437.1818	CHO	0.409	1.364	8	0.200
C23H34O8	437.2181	CHO	0.348	1.478	7	0.158
C21H17N3O8	438.0946	CHNO	0.381	0.810	15	0.679
C22H21N3O7	438.1309	CHNO	0.318	0.955	14	0.581
C23H20O9	439.1036	CHO	0.391	0.870	14	0.514
C20H24O9S1	439.1069	CHOS	0.450	1.200	9	0.241
C20H24O11	439.1247	CHO	0.550	1.200	9	0.241

C24H24O8	439.1400	CHO	0.333	1.000	13	0.450
C21H28O10	439.1612	CHO	0.476	1.333	8	0.188
C25H28O7	439.1764	CHO	0.280	1.120	12	0.395
C22H32O9	439.1975	CHO	0.409	1.455	7	0.143
C26H32O6	439.2127	CHO	0.231	1.231	11	0.348
C23H36O8	439.2337	CHO	0.348	1.565	6	0.105
C21H19N3O8	440.1101	CHNO	0.381	0.905	14	0.607
C22H23N3O7	440.1463	CHNO	0.318	1.045	13	0.516
C19H22O10S1	441.0862	CHOS	0.526	1.158	9	0.231
C23H22O9	441.1193	CHO	0.391	0.957	13	0.459
C20H26O9S1	441.1226	CHOS	0.450	1.300	8	0.172
C20H26O11	441.1404	CHO	0.550	1.300	8	0.172
C24H26O8	441.1557	CHO	0.333	1.083	12	0.400
C21H30O8S1	441.1590	CHOS	0.381	1.429	7	0.125
C21H30O10	441.1768	CHO	0.476	1.429	7	0.125
C25H30O7	441.1920	CHO	0.280	1.200	11	0.349
C22H34O9	441.2132	CHO	0.409	1.545	6	0.086
C21H21N3O8	442.1257	CHNO	0.381	1.000	13	0.536
C23H25N1O8	442.1507	CHNO	0.348	1.087	12	0.417
C22H25N3O7	442.1622	CHNO	0.318	1.136	12	0.452
C19H24O10S1	443.1019	CHOS	0.526	1.263	8	0.154
C23H24O9	443.1349	CHO	0.391	1.043	12	0.405
C20H28O9S1	443.1383	CHOS	0.450	1.400	7	0.103
C24H28O8	443.1713	CHO	0.333	1.167	11	0.350
C21H32O8S1	443.1741	CHOS	0.381	1.524	6	0.063
C21H32O10	443.1923	CHO	0.476	1.524	6	0.063
C25H32O7	443.2076	CHO	0.280	1.280	10	0.302
C21H23N3O8	444.1415	CHNO	0.381	1.095	12	0.464
C23H27N1O8	444.1664	CHNO	0.348	1.174	11	0.361
C22H27N3O7	444.1776	CHNO	0.318	1.227	11	0.387
C22H22O10	445.1141	CHO	0.455	1.000	12	0.412
C19H26O10S1	445.1174	CHOS	0.526	1.368	7	0.077
C23H26O9	445.1505	CHO	0.391	1.130	11	0.351
C20H30O9S1	445.1536	CHOS	0.450	1.500	6	0.034
C24H30O8	445.1869	CHO	0.333	1.250	10	0.300
C25H34O7	445.2233	CHO	0.280	1.360	9	0.256
C22H25N1O9	446.1459	CHNO	0.409	1.136	11	0.364
C23H29N1O8	446.1821	CHNO	0.348	1.261	10	0.306
C22H29N3O7	446.1935	CHNO	0.318	1.318	10	0.323
C22H24O10	447.1297	CHO	0.455	1.091	11	0.353
C19H28O10S1	447.1328	CHOS	0.526	1.474	6	0.000
C23H28O9	447.1660	CHO	0.391	1.217	10	0.297
C20H32O9S1	447.1690	CHOS	0.450	1.600	5	-0.034
C24H32O8	447.2025	CHO	0.333	1.333	9	0.250
C25H36O7	447.2389	CHO	0.280	1.440	8	0.209
C22H27N1O9	448.1616	CHNO	0.409	1.227	10	0.303
C21H22O11	449.1087	CHO	0.524	1.048	11	0.355
C22H26O8S1	449.1275	CHOS	0.364	1.182	10	0.294
C22H26O10	449.1453	CHO	0.455	1.182	10	0.294
C23H30O9	449.1816	CHO	0.391	1.304	9	0.243

C24H34O8	449.2180	CHO	0.333	1.417	8	0.200
C21H24O9S1	451.1069	CHOS	0.429	1.143	10	0.290
C21H24O11	451.1244	CHO	0.524	1.143	10	0.290
C22H28O8S1	451.1431	CHOS	0.364	1.273	9	0.235
C22H28O10	451.1609	CHO	0.455	1.273	9	0.235
C26H28O7	451.1762	CHO	0.269	1.077	13	0.422
C23H32O9	451.1973	CHO	0.391	1.391	8	0.189
C24H36O8	451.2337	CHO	0.333	1.500	7	0.150
C23H23N3O7	452.1466	CHNO	0.304	1.000	14	0.545
C20H22O10S1	453.0861	CHOS	0.500	1.100	10	0.286
C24H22O9	453.1191	CHO	0.375	0.917	14	0.487
C21H26O9S1	453.1225	CHOS	0.429	1.238	9	0.226
C21H26O11	453.1403	CHO	0.524	1.238	9	0.226
C25H26O8	453.1554	CHO	0.320	1.040	13	0.429
C22H30O8S1	453.1588	CHOS	0.364	1.364	8	0.176
C22H30O10	453.1766	CHO	0.455	1.364	8	0.176
C26H30O7	453.1918	CHO	0.269	1.154	12	0.378
C23H34O9	453.2128	CHO	0.391	1.478	7	0.135
C22H21N3O8	454.1256	CHNO	0.364	0.955	14	0.567
C20H24O10S1	455.1018	CHOS	0.500	1.200	9	0.214
C24H24O9	455.1348	CHO	0.375	1.000	13	0.436
C21H28O9S1	455.1382	CHOS	0.429	1.333	8	0.161
C21H28O11	455.1561	CHO	0.524	1.333	8	0.161
C25H28O8	455.1712	CHO	0.320	1.120	12	0.381
C22H32O8S1	455.1743	CHOS	0.364	1.455	7	0.118
C22H32O10	455.1923	CHO	0.455	1.455	7	0.118
C26H32O7	455.2077	CHO	0.269	1.231	11	0.333
C23H36O9	455.2288	CHO	0.391	1.565	6	0.081
C20H27N1O9S1	456.1332	CHNOS	0.450	1.350	8	0.148
C22H23N3O8	456.1414	CHNO	0.364	1.045	13	0.500
C23H22O10	457.1148	CHO	0.435	0.957	13	0.444
C20H26O10S1	457.1175	CHOS	0.500	1.300	8	0.143
C24H26O9	457.1506	CHO	0.375	1.083	12	0.385
C21H30O9S1	457.1538	CHOS	0.429	1.429	7	0.097
C21H30O11	457.1714	CHO	0.524	1.429	7	0.097
C25H30O8	457.1869	CHO	0.320	1.200	11	0.333
C22H34O10	457.2081	CHO	0.455	1.545	6	0.059
C26H34O7	457.2233	CHO	0.269	1.308	10	0.289
C24H29N1O8	458.1819	CHNO	0.333	1.208	11	0.342
C19H24O11S1	459.0968	CHOS	0.579	1.263	8	0.120
C23H24O10	459.1297	CHO	0.435	1.043	12	0.389
C20H28O10S1	459.1331	CHOS	0.500	1.400	7	0.071
C24H28O9	459.1661	CHO	0.375	1.167	11	0.333
C25H32O8	459.2025	CHO	0.320	1.280	10	0.286
C26H36O7	459.2390	CHO	0.269	1.385	9	0.244
C23H27N1O9	460.1614	CHNO	0.391	1.174	11	0.343
C22H22O11	461.1090	CHO	0.500	1.000	12	0.394
C19H26O11S1	461.1124	CHOS	0.579	1.368	7	0.040
C23H26O10	461.1453	CHO	0.435	1.130	11	0.333
C20H30O10S1	461.1486	CHOS	0.500	1.500	6	0.000

C24H30O9	461.1817	CHO	0.375	1.250	10	0.282
C25H34O8	461.2181	CHO	0.320	1.360	9	0.238
C26H38O7	461.2545	CHO	0.269	1.462	8	0.200
C23H29N1O9	462.1770	CHNO	0.391	1.261	10	0.286
C22H24O9S1	463.1066	CHOS	0.409	1.091	11	0.333
C22H24O11	463.1245	CHO	0.500	1.091	11	0.333
C19H28O11S1	463.1277	CHOS	0.579	1.474	6	-0.040
C23H28O10	463.1608	CHO	0.435	1.217	10	0.278
C24H32O9	463.1972	CHO	0.375	1.333	9	0.231
C25H36O8	463.2337	CHO	0.320	1.440	8	0.190
C22H26O9S1	465.1224	CHOS	0.409	1.182	10	0.273
C22H26O11	465.1402	CHO	0.500	1.182	10	0.273
C23H30O8S1	465.1588	CHOS	0.348	1.304	9	0.222
C23H30O10	465.1766	CHO	0.435	1.304	9	0.222
C24H34O9	465.2129	CHO	0.375	1.417	8	0.179
C21H24O10S1	467.1018	CHOS	0.476	1.143	10	0.267
C25H24O9	467.1346	CHO	0.360	0.960	14	0.463
C22H28O9S1	467.1382	CHOS	0.409	1.273	9	0.212
C22H28O11	467.1560	CHO	0.500	1.273	9	0.212
C23H32O8S1	467.1738	CHOS	0.348	1.391	8	0.167
C23H32O10	467.1924	CHO	0.435	1.391	8	0.167
C27H32O7	467.2075	CHO	0.259	1.185	12	0.362
C24H36O9	467.2286	CHO	0.375	1.500	7	0.128
C23H23N3O8	468.1411	CHNO	0.348	1.000	14	0.531
C21H26O10S1	469.1175	CHOS	0.476	1.238	9	0.200
C25H26O9	469.1505	CHO	0.360	1.040	13	0.415
C22H30O9S1	469.1540	CHOS	0.409	1.364	8	0.152
C22H30O11	469.1717	CHO	0.500	1.364	8	0.152
C26H30O8	469.1870	CHO	0.308	1.154	12	0.364
C23H34O10	469.2079	CHO	0.435	1.478	7	0.111
C27H34O7	469.2233	CHO	0.259	1.259	11	0.319
C23H25N3O8	470.1570	CHNO	0.348	1.087	13	0.469
C24H24O10	471.1296	CHO	0.417	1.000	13	0.421
C21H28O10S1	471.1331	CHOS	0.476	1.333	8	0.133
C25H28O9	471.1660	CHO	0.360	1.120	12	0.366
C22H32O9S1	471.1690	CHOS	0.409	1.455	7	0.091
C22H32O11	471.1870	CHO	0.500	1.455	7	0.091
C26H32O8	471.2025	CHO	0.308	1.231	11	0.318
C27H36O7	471.2387	CHO	0.259	1.333	10	0.277
C25H31N1O8	472.1977	CHNO	0.320	1.240	11	0.325
C20H26O11S1	473.1122	CHOS	0.550	1.300	8	0.111
C24H26O10	473.1452	CHO	0.417	1.083	12	0.368
C21H30O10S1	473.1486	CHOS	0.476	1.429	7	0.067
C25H30O9	473.1816	CHO	0.360	1.200	11	0.317
C26H34O8	473.2180	CHO	0.308	1.308	10	0.273
C23H24O11	475.1245	CHO	0.478	1.043	12	0.371
C24H28O10	475.1609	CHO	0.417	1.167	11	0.316
C21H32O10S1	475.1635	CHOS	0.476	1.524	6	0.000
C25H32O9	475.1973	CHO	0.360	1.280	10	0.268
C26H36O8	475.2333	CHO	0.308	1.385	9	0.227

C22H22O10S1	477.0863	CHOS	0.455	1.000	12	0.375
C23H26O9S1	477.1225	CHOS	0.391	1.130	11	0.314
C23H26O11	477.1403	CHO	0.478	1.130	11	0.314
C24H30O10	477.1767	CHO	0.417	1.250	10	0.263
C25H34O9	477.2131	CHO	0.360	1.360	9	0.220
C26H38O8	477.2494	CHO	0.308	1.462	8	0.182
C23H28O11	479.1560	CHO	0.478	1.217	10	0.257
C24H32O10	479.1924	CHO	0.417	1.333	9	0.211
C25H36O9	479.2288	CHO	0.360	1.440	8	0.171
C24H23N3O8	480.1414	CHNO	0.333	0.958	15	0.559
C25H27N3O7	480.1778	CHNO	0.280	1.080	14	0.486
C22H26O10S1	481.1174	CHOS	0.455	1.182	10	0.250
C22H26O12	481.1351	CHO	0.545	1.182	10	0.250
C26H26O9	481.1504	CHO	0.346	1.000	14	0.442
C23H30O9S1	481.1537	CHOS	0.391	1.304	9	0.200
C23H30O11	481.1715	CHO	0.478	1.304	9	0.200
C27H30O8	481.1868	CHO	0.296	1.111	13	0.391
C24H34O10	481.2079	CHO	0.417	1.417	8	0.158
C28H34O7	481.2229	CHO	0.250	1.214	12	0.347
C24H25N3O8	482.1571	CHNO	0.333	1.042	14	0.500
C22H28O10S1	483.1329	CHOS	0.455	1.273	9	0.188
C22H28O12	483.1507	CHO	0.545	1.273	9	0.188
C26H28O9	483.1659	CHO	0.346	1.077	13	0.395
C23H32O9S1	483.1687	CHOS	0.391	1.391	8	0.143
C23H32O11	483.1871	CHO	0.478	1.391	8	0.143
C27H32O8	483.2023	CHO	0.296	1.185	12	0.348
C24H36O10	483.2237	CHO	0.417	1.500	7	0.105
C21H26O11S1	485.1123	CHOS	0.524	1.238	9	0.172
C25H26O10	485.1453	CHO	0.400	1.040	13	0.400
C22H30O10S1	485.1488	CHOS	0.455	1.364	8	0.125
C26H30O9	485.1817	CHO	0.346	1.154	12	0.349
C27H34O8	485.2181	CHO	0.296	1.259	11	0.304
C24H24O11	487.1246	CHO	0.458	1.000	13	0.405
C21H28O11S1	487.1281	CHOS	0.524	1.333	8	0.103
C25H28O10	487.1611	CHO	0.400	1.120	12	0.350
C22H32O10S1	487.1639	CHOS	0.455	1.455	7	0.063
C26H32O9	487.1974	CHO	0.346	1.231	11	0.302
C27H36O8	487.2339	CHO	0.296	1.333	10	0.261
C28H40O7	487.2704	CHO	0.250	1.429	9	0.224
C24H26O11	489.1403	CHO	0.458	1.083	12	0.351
C21H30O11S1	489.1434	CHOS	0.524	1.429	7	0.034
C25H30O10	489.1766	CHO	0.400	1.200	11	0.300
C26H34O9	489.2130	CHO	0.346	1.308	10	0.256
C27H38O8	489.2494	CHO	0.296	1.407	9	0.217
C23H24O10S1	491.1016	CHOS	0.435	1.043	12	0.353
C23H24O12	491.1191	CHO	0.522	1.043	12	0.353
C24H28O11	491.1558	CHO	0.458	1.167	11	0.297
C25H32O10	491.1922	CHO	0.400	1.280	10	0.250
C26H36O9	491.2285	CHO	0.346	1.385	9	0.209
C23H26O10S1	493.1174	CHOS	0.435	1.130	11	0.294

C23H26O12	493.1352	CHO	0.522	1.130	11	0.294
C24H30O9S1	493.1539	CHOS	0.375	1.250	10	0.243
C24H30O11	493.1716	CHO	0.458	1.250	10	0.243
C28H30O8	493.1870	CHO	0.286	1.071	14	0.417
C25H34O8S1	493.1902	CHOS	0.320	1.360	9	0.200
C25H34O10	493.2080	CHO	0.400	1.360	9	0.200
C26H38O9	493.2443	CHO	0.346	1.462	8	0.163
C25H25N3O8	494.1570	CHNO	0.320	1.000	15	0.528
C23H28O10S1	495.1331	CHOS	0.435	1.217	10	0.235
C23H28O12	495.1509	CHO	0.522	1.217	10	0.235
C24H32O9S1	495.1694	CHOS	0.375	1.333	9	0.189
C24H32O11	495.1873	CHO	0.458	1.333	9	0.189
C28H32O8	495.2026	CHO	0.286	1.143	13	0.375
C25H36O10	495.2237	CHO	0.400	1.440	8	0.150
C29H36O7	495.2391	CHO	0.241	1.241	12	0.333
C26H40O9	495.2600	CHO	0.346	1.538	7	0.116
C26H26O10	497.1453	CHO	0.385	1.000	14	0.429
C23H30O10S1	497.1485	CHOS	0.435	1.304	9	0.176
C23H30O12	497.1663	CHO	0.522	1.304	9	0.176
C27H30O9	497.1817	CHO	0.333	1.111	13	0.378
C24H34O11	497.2029	CHO	0.458	1.417	8	0.135
C22H28O11S1	499.1281	CHOS	0.500	1.273	9	0.161
C26H28O10	499.1609	CHO	0.385	1.077	13	0.381
C27H32O9	499.1973	CHO	0.333	1.185	12	0.333
C28H36O8	499.2336	CHO	0.286	1.286	11	0.292
C25H26O11	501.1404	CHO	0.440	1.040	13	0.385
C22H30O11S1	501.1436	CHOS	0.500	1.364	8	0.097
C26H30O10	501.1767	CHO	0.385	1.154	12	0.333
C27H34O9	501.2131	CHO	0.333	1.259	11	0.289
C28H38O8	501.2495	CHO	0.286	1.357	10	0.250
C25H28O11	503.1559	CHO	0.440	1.120	12	0.333
C26H32O10	503.1923	CHO	0.385	1.231	11	0.286
C27H36O9	503.2286	CHO	0.333	1.333	10	0.244
C28H40O8	503.2651	CHO	0.286	1.429	9	0.208
C24H26O12	505.1350	CHO	0.500	1.083	12	0.333
C25H30O11	505.1714	CHO	0.440	1.200	11	0.282
C26H34O10	505.2078	CHO	0.385	1.308	10	0.238
C27H38O9	505.2442	CHO	0.333	1.407	9	0.200
C28H42O8	505.2806	CHO	0.286	1.500	8	0.167
C24H28O10S1	507.1332	CHOS	0.417	1.167	11	0.278
C24H28O12	507.1509	CHO	0.500	1.167	11	0.278
C25H32O9S1	507.1695	CHOS	0.360	1.280	10	0.231
C25H32O11	507.1873	CHO	0.440	1.280	10	0.231
C26H36O10	507.2237	CHO	0.385	1.385	9	0.190
C23H26O11S1	509.1123	CHOS	0.478	1.130	11	0.273
C24H30O10S1	509.1488	CHOS	0.417	1.250	10	0.222
C24H30O12	509.1665	CHO	0.500	1.250	10	0.222
C25H34O9S1	509.1849	CHOS	0.360	1.360	9	0.179
C25H34O11	509.2029	CHO	0.440	1.360	9	0.179
C26H38O10	509.2394	CHO	0.385	1.462	8	0.143

C23H28O11S1	511.1280	CHOS	0.478	1.217	10	0.212
C24H32O10S1	511.1646	CHOS	0.417	1.333	9	0.167
C24H32O12	511.1820	CHO	0.500	1.333	9	0.167
C28H32O9	511.1973	CHO	0.321	1.143	13	0.362
C25H36O11	511.2185	CHO	0.440	1.440	8	0.128
C29H36O8	511.2337	CHO	0.276	1.241	12	0.320
C26H40O10	511.2550	CHO	0.385	1.538	7	0.095
C23H30O11S1	513.1436	CHOS	0.478	1.304	9	0.152
C27H30O10	513.1768	CHO	0.370	1.111	13	0.364
C28H34O9	513.2129	CHO	0.321	1.214	12	0.319
C29H38O8	513.2494	CHO	0.276	1.310	11	0.280
C26H28O11	515.1558	CHO	0.423	1.077	13	0.366
C23H32O11S1	515.1595	CHOS	0.478	1.391	8	0.091
C27H32O10	515.1921	CHO	0.370	1.185	12	0.318
C28H36O9	515.2288	CHO	0.321	1.286	11	0.277
C26H30O11	517.1714	CHO	0.423	1.154	12	0.317
C27H34O10	517.2077	CHO	0.370	1.259	11	0.273
C28H38O9	517.2441	CHO	0.321	1.357	10	0.234
C25H28O10S1	519.1333	CHOS	0.400	1.120	12	0.316
C25H28O12	519.1508	CHO	0.480	1.120	12	0.316
C26H32O11	519.1874	CHO	0.423	1.231	11	0.268
C27H36O10	519.2237	CHO	0.370	1.333	10	0.227
C28H40O9	519.2600	CHO	0.321	1.429	9	0.191
C25H30O10S1	521.1487	CHOS	0.400	1.200	11	0.263
C25H30O12	521.1665	CHO	0.480	1.200	11	0.263
C26H34O9S1	521.1850	CHOS	0.346	1.308	10	0.220
C26H34O11	521.2029	CHO	0.423	1.308	10	0.220
C27H38O10	521.2393	CHO	0.370	1.407	9	0.182
C28H42O9	521.2759	CHO	0.321	1.500	8	0.149
C24H28O11S1	523.1283	CHOS	0.458	1.167	11	0.257
C25H32O10S1	523.1643	CHOS	0.400	1.280	10	0.211
C25H32O12	523.1820	CHO	0.480	1.280	10	0.211
C26H36O11	523.2182	CHO	0.423	1.385	9	0.171
C27H40O10	523.2548	CHO	0.370	1.481	8	0.136
C24H30O11S1	525.1438	CHOS	0.458	1.250	10	0.200
C25H34O12	525.1978	CHO	0.480	1.360	9	0.158
C26H38O11	525.2340	CHO	0.423	1.462	8	0.122
C30H38O8	525.2495	CHO	0.267	1.267	12	0.308
C28H32O10	527.1923	CHO	0.357	1.143	13	0.348
C29H36O9	527.2288	CHO	0.310	1.241	12	0.306
C27H30O11	529.1714	CHO	0.407	1.111	13	0.349
C28H34O10	529.2078	CHO	0.357	1.214	12	0.304
C29H38O9	529.2442	CHO	0.310	1.310	11	0.265
C27H32O11	531.1873	CHO	0.407	1.185	12	0.302
C28H36O10	531.2237	CHO	0.357	1.286	11	0.261
C26H30O12	533.1667	CHO	0.462	1.154	12	0.300
C27H34O11	533.2027	CHO	0.407	1.259	11	0.256
C28H38O10	533.2392	CHO	0.357	1.357	10	0.217
C26H32O10S1	535.1643	CHOS	0.385	1.231	11	0.250
C26H32O12	535.1820	CHO	0.462	1.231	11	0.250

C27H36O11	535.2185	CHO	0.407	1.333	10	0.209
C28H40O10	535.2547	CHO	0.357	1.429	9	0.174
C26H34O10S1	537.1802	CHOS	0.385	1.308	10	0.200
C26H34O12	537.1977	CHO	0.462	1.308	10	0.200
C25H32O11S1	539.1593	CHOS	0.440	1.280	10	0.189
C30H36O9	539.2285	CHO	0.300	1.200	13	0.333
C29H34O10	541.2080	CHO	0.345	1.172	13	0.333
C30H38O9	541.2444	CHO	0.300	1.267	12	0.294
C29H36O10	543.2235	CHO	0.345	1.241	12	0.292
C28H34O11	545.2028	CHO	0.393	1.214	12	0.289
C29H38O10	545.2392	CHO	0.345	1.310	11	0.250
C30H42O9	545.2757	CHO	0.300	1.400	10	0.216
C27H32O12	547.1822	CHO	0.444	1.185	12	0.286
C28H36O11	547.2186	CHO	0.393	1.286	11	0.244
C29H40O10	547.2549	CHO	0.345	1.379	10	0.208
C30H44O9	547.2913	CHO	0.300	1.467	9	0.176
C27H34O12	549.1975	CHO	0.444	1.259	11	0.238
C28H38O11	549.2337	CHO	0.393	1.357	10	0.200
C26H32O11S1	551.1594	CHOS	0.423	1.231	11	0.231
C27H36O12	551.2136	CHO	0.444	1.333	10	0.190
C26H34O11S1	553.1748	CHOS	0.423	1.308	10	0.179
C30H36O10	555.2236	CHO	0.333	1.200	13	0.320
C29H34O11	557.2028	CHO	0.379	1.172	13	0.319
C30H38O10	557.2392	CHO	0.333	1.267	12	0.280
C29H36O11	559.2183	CHO	0.379	1.241	12	0.277
C30H40O10	559.2547	CHO	0.333	1.333	11	0.240
C28H34O12	561.1976	CHO	0.429	1.214	12	0.273
C29H38O11	561.2341	CHO	0.379	1.310	11	0.234
C30H42O10	561.2706	CHO	0.333	1.400	10	0.200
C28H36O12	563.2132	CHO	0.429	1.286	11	0.227
C29H40O11	563.2497	CHO	0.379	1.379	10	0.191
C30H36O11	571.2184	CHO	0.367	1.200	13	0.306
C31H40O10	571.2549	CHO	0.323	1.290	12	0.269
C30H38O11	573.2342	CHO	0.367	1.267	12	0.265
C31H42O10	573.2705	CHO	0.323	1.355	11	0.231
C29H36O12	575.2135	CHO	0.414	1.241	12	0.261
C30H40O11	575.2498	CHO	0.367	1.333	11	0.224
C29H38O12	577.2291	CHO	0.414	1.310	11	0.217
C31H38O11	585.2341	CHO	0.355	1.226	13	0.294
C31H40O11	587.2499	CHO	0.355	1.290	12	0.255
C30H38O12	589.2288	CHO	0.400	1.267	12	0.250
C30H40O12	591.2447	CHO	0.400	1.333	11	0.208
C32H42O11	601.2653	CHO	0.344	1.313	12	0.245
C31H40O12	603.2451	CHO	0.387	1.290	12	0.240

Table S2. Formula, *m/z*, element class, O/C, H/C, DEB, and AI_{mod} of the 638 accumulated molecular formulas existed during the 90-day incubation.

Formula	m/z	Class	O/C	H/C	DBE	AI_{mod}
C11H12O4	207.0663	CHO	0.364	1.091	6	0.444
C12H16O3	207.1027	CHO	0.250	1.333	5	0.333
C11H14O4	209.0819	CHO	0.364	1.273	5	0.333
C12H18O3	209.1183	CHO	0.250	1.500	4	0.238
C10H12O5	211.0612	CHO	0.500	1.200	5	0.333
C10H14O5	213.0768	CHO	0.500	1.400	4	0.200
C12H12O4	219.0663	CHO	0.333	1.000	7	0.500
C11H10O5	221.0455	CHO	0.455	0.909	7	0.529
C12H14O4	221.0819	CHO	0.333	1.167	6	0.400
C13H18O3	221.1183	CHO	0.231	1.385	5	0.304
C11H12O5	223.0612	CHO	0.455	1.091	6	0.412
C12H16O4	223.0976	CHO	0.333	1.333	5	0.300
C13H20O3	223.1340	CHO	0.231	1.538	4	0.217
C11H14O5	225.0768	CHO	0.455	1.273	5	0.294
C12H18O4	225.1132	CHO	0.333	1.500	4	0.200
C11H16O5	227.0925	CHO	0.455	1.455	4	0.176
C12H10O5	233.0455	CHO	0.417	0.833	8	0.579
C13H14O4	233.0819	CHO	0.308	1.077	7	0.455
C12H12O5	235.0612	CHO	0.417	1.000	7	0.474
C13H16O4	235.0976	CHO	0.308	1.231	6	0.364
C14H20O3	235.1340	CHO	0.214	1.429	5	0.280
C11H10O6	237.0405	CHO	0.545	0.909	7	0.500
C12H14O5	237.0768	CHO	0.417	1.167	6	0.368
C11H13N1O5	238.0721	CHNO	0.455	1.182	6	0.400
C11H12O6	239.0561	CHO	0.545	1.091	6	0.375
C12H16O5	239.0925	CHO	0.417	1.333	5	0.263
C11H14O6	241.0718	CHO	0.545	1.273	5	0.250
C12H18O5	241.1081	CHO	0.417	1.500	4	0.158
C10H14O5S1	245.0489	CHOS	0.500	1.400	4	0.077
C13H12O5	247.0612	CHO	0.385	0.923	8	0.524
C14H16O4	247.0976	CHO	0.286	1.143	7	0.417
C12H10O6	249.0405	CHO	0.500	0.833	8	0.556
C13H14O5	249.0768	CHO	0.385	1.077	7	0.429
C14H18O4	249.1132	CHO	0.286	1.286	6	0.333
C15H22O3	249.1496	CHO	0.200	1.467	5	0.259
C12H13N1O5	250.0721	CHNO	0.417	1.083	7	0.471
C12H12O6	251.0561	CHO	0.500	1.000	7	0.444
C13H16O5	251.0925	CHO	0.385	1.231	6	0.333
C14H20O4	251.1289	CHO	0.286	1.429	5	0.250
C12H14O6	253.0718	CHO	0.500	1.167	6	0.333
C13H18O5	253.1081	CHO	0.385	1.385	5	0.238
C12H16O6	255.0874	CHO	0.500	1.333	5	0.222
C13H20O5	255.1238	CHO	0.385	1.538	4	0.143
C11H15N1O6	256.0827	CHNO	0.545	1.364	5	0.214
C14H14O5	261.0768	CHO	0.357	1.000	8	0.478
C15H18O4	261.1132	CHO	0.267	1.200	7	0.385
C14H17N1O4	262.1085	CHNO	0.286	1.214	7	0.409

C13H12O6	263.0561	CHO	0.462	0.923	8	0.500
C14H16O5	263.0925	CHO	0.357	1.143	7	0.391
C15H20O4	263.1289	CHO	0.267	1.333	6	0.308
C16H24O3	263.1653	CHO	0.188	1.500	5	0.241
C13H15N1O5	264.0877	CHNO	0.385	1.154	7	0.421
C12H10O7	265.0354	CHO	0.583	0.833	8	0.529
C13H14O6	265.0718	CHO	0.462	1.077	7	0.400
C14H18O5	265.1081	CHO	0.357	1.286	6	0.304
C13H17N1O5	266.1034	CHNO	0.385	1.308	6	0.316
C12H12O7	267.0510	CHO	0.583	1.000	7	0.412
C13H16O6	267.0874	CHO	0.462	1.231	6	0.300
C14H20O5	267.1238	CHO	0.357	1.429	5	0.217
C13H19N1O5	268.1190	CHNO	0.385	1.462	5	0.211
C12H14O7	269.0667	CHO	0.583	1.167	6	0.294
C11H14O6S1	273.0438	CHOS	0.545	1.273	5	0.143
C14H12O6	275.0561	CHO	0.429	0.857	9	0.545
C15H16O5	275.0925	CHO	0.333	1.067	8	0.440
C16H20O4	275.1289	CHO	0.250	1.250	7	0.357
C14H14O6	277.0718	CHO	0.429	1.000	8	0.455
C15H18O5	277.1081	CHO	0.333	1.200	7	0.360
C16H22O4	277.1445	CHO	0.250	1.375	6	0.286
C14H17N1O5	278.1034	CHNO	0.357	1.214	7	0.381
C14H16O6	279.0874	CHO	0.429	1.143	7	0.364
C15H20O5	279.1238	CHO	0.333	1.333	6	0.280
C16H24O4	279.1602	CHO	0.250	1.500	5	0.214
C14H19N1O5	280.1190	CHNO	0.357	1.357	6	0.286
C13H14O7	281.0667	CHO	0.538	1.077	7	0.368
C14H18O6	281.1031	CHO	0.429	1.286	6	0.273
C15H22O5	281.1394	CHO	0.333	1.467	5	0.200
C14H21N1O5	282.1347	CHNO	0.357	1.500	5	0.190
C12H12O6S1	283.0282	CHOS	0.500	1.000	7	0.375
C13H16O5S1	283.0646	CHOS	0.385	1.231	6	0.263
C13H16O7	283.0823	CHO	0.538	1.231	6	0.263
C14H20O6	283.1187	CHO	0.429	1.429	5	0.182
C15H14O6	289.0718	CHO	0.400	0.933	9	0.500
C16H18O5	289.1081	CHO	0.313	1.125	8	0.407
C17H22O4	289.1445	CHO	0.235	1.294	7	0.333
C15H17N1O5	290.1034	CHNO	0.333	1.133	8	0.435
C14H12O7	291.0510	CHO	0.500	0.857	9	0.524
C15H16O6	291.0874	CHO	0.400	1.067	8	0.417
C16H20O5	291.1238	CHO	0.313	1.250	7	0.333
C17H24O4	291.1602	CHO	0.235	1.412	6	0.267
C14H15N1O6	292.0827	CHNO	0.429	1.071	8	0.450
C15H19N1O5	292.1190	CHNO	0.333	1.267	7	0.348
C14H14O7	293.0667	CHO	0.500	1.000	8	0.429
C15H18O6	293.1031	CHO	0.400	1.200	7	0.333
C16H22O5	293.1394	CHO	0.313	1.375	6	0.259
C14H17N1O6	294.0983	CHNO	0.429	1.214	7	0.350
C15H21N1O5	294.1347	CHNO	0.333	1.400	6	0.261
C14H16O5S1	295.0646	CHOS	0.357	1.143	7	0.333

C14H16O7	295.0823	CHO	0.500	1.143	7	0.333
C15H20O6	295.1187	CHO	0.400	1.333	6	0.250
C16H24O5	295.1551	CHO	0.313	1.500	5	0.185
C13H14O8	297.0616	CHO	0.615	1.077	7	0.333
C14H18O5S1	297.0802	CHOS	0.357	1.286	6	0.238
C15H22O6	297.1344	CHO	0.400	1.467	5	0.167
C13H16O6S1	299.0595	CHOS	0.462	1.231	6	0.222
C16H14O6	301.0718	CHO	0.375	0.875	10	0.538
C13H18O6S1	301.0751	CHOS	0.462	1.385	5	0.111
C15H12O7	303.0510	CHO	0.467	0.800	10	0.565
C16H16O6	303.0874	CHO	0.375	1.000	9	0.462
C17H20O5	303.1238	CHO	0.294	1.176	8	0.379
C18H24O4	303.1602	CHO	0.222	1.333	7	0.313
C16H19N1O5	304.1190	CHNO	0.313	1.188	8	0.400
C15H14O7	305.0667	CHO	0.467	0.933	9	0.478
C16H18O6	305.1031	CHO	0.375	1.125	8	0.385
C18H26O4	305.1758	CHO	0.222	1.444	6	0.250
C15H17N1O6	306.0983	CHNO	0.400	1.133	8	0.409
C16H21N1O5	306.1347	CHNO	0.313	1.313	7	0.320
C14H12O8	307.0459	CHO	0.571	0.857	9	0.500
C15H16O7	307.0823	CHO	0.467	1.067	8	0.391
C16H20O6	307.1187	CHO	0.375	1.250	7	0.308
C17H24O5	307.1551	CHO	0.294	1.412	6	0.241
C16H23N1O5	308.1503	CHNO	0.313	1.438	6	0.240
C14H14O6S1	309.0438	CHOS	0.429	1.000	8	0.400
C14H14O8	309.0616	CHO	0.571	1.000	8	0.400
C15H18O7	309.0980	CHO	0.467	1.200	7	0.304
C16H22O6	309.1344	CHO	0.375	1.375	6	0.231
C15H21N1O6	310.1296	CHNO	0.400	1.400	6	0.227
C16H24O6	311.1500	CHO	0.375	1.500	5	0.154
C14H18O6S1	313.0751	CHOS	0.429	1.286	6	0.200
C13H16O7S1	315.0544	CHOS	0.538	1.231	6	0.176
C17H16O6	315.0874	CHO	0.353	0.941	10	0.500
C14H20O6S1	315.0908	CHOS	0.429	1.429	5	0.100
C16H14O7	317.0667	CHO	0.438	0.875	10	0.520
C17H18O6	317.1031	CHO	0.353	1.059	9	0.429
C18H22O5	317.1394	CHO	0.278	1.222	8	0.355
C19H26O4	317.1758	CHO	0.211	1.368	7	0.294
C16H17N1O6	318.0983	CHNO	0.375	1.063	9	0.458
C17H21N1O5	318.1347	CHNO	0.294	1.235	8	0.370
C16H16O7	319.0823	CHO	0.438	1.000	9	0.440
C17H20O6	319.1187	CHO	0.353	1.176	8	0.357
C16H20N2O5	319.1299	CHNO	0.313	1.250	8	0.391
C18H24O5	319.1551	CHO	0.278	1.333	7	0.290
C19H28O4	319.1915	CHO	0.211	1.474	6	0.235
C16H19N1O6	320.1140	CHNO	0.375	1.188	8	0.375
C17H23N1O5	320.1503	CHNO	0.294	1.353	7	0.296
C15H14O8	321.0616	CHO	0.533	0.933	9	0.455
C16H18O7	321.0980	CHO	0.438	1.125	8	0.360
C17H22O6	321.1344	CHO	0.353	1.294	7	0.286

C18H26O5	321.1707	CHO	0.278	1.444	6	0.226
C16H21N1O6	322.1296	CHNO	0.375	1.313	7	0.292
C15H16O6S1	323.0595	CHOS	0.400	1.067	8	0.364
C16H20O7	323.1136	CHO	0.438	1.250	7	0.280
C17H24O6	323.1500	CHO	0.353	1.412	6	0.214
C14H14O7S1	325.0387	CHOS	0.500	1.000	8	0.368
C15H18O6S1	325.0751	CHOS	0.400	1.200	7	0.273
C16H22O5S1	325.1115	CHOS	0.313	1.375	6	0.200
C14H16O7S1	327.0544	CHOS	0.500	1.143	7	0.263
C18H18O6	329.1031	CHO	0.333	1.000	10	0.467
C15H22O6S1	329.1064	CHOS	0.400	1.467	5	0.091
C17H16O7	331.0823	CHO	0.412	0.941	10	0.481
C18H20O6	331.1187	CHO	0.333	1.111	9	0.400
C19H24O5	331.1551	CHO	0.263	1.263	8	0.333
C20H28O4	331.1915	CHO	0.200	1.400	7	0.278
C17H19N1O6	332.1140	CHNO	0.353	1.118	9	0.423
C16H14O8	333.0616	CHO	0.500	0.875	10	0.500
C17H18O7	333.0980	CHO	0.412	1.059	9	0.407
C18H22O6	333.1344	CHO	0.333	1.222	8	0.333
C17H22N2O5	333.1456	CHNO	0.294	1.294	8	0.360
C19H26O5	333.1707	CHO	0.263	1.368	7	0.273
C17H21N1O6	334.1296	CHNO	0.353	1.235	8	0.346
C18H25N1O5	334.1660	CHNO	0.278	1.389	7	0.276
C16H16O8	335.0772	CHO	0.500	1.000	9	0.417
C17H20O7	335.1136	CHO	0.412	1.176	8	0.333
C18H24O4S1	335.1323	CHOS	0.222	1.333	7	0.267
C18H24O6	335.1500	CHO	0.333	1.333	7	0.267
C19H28O5	335.1864	CHO	0.263	1.474	6	0.212
C17H23N1O6	336.1453	CHNO	0.353	1.353	7	0.269
C16H18O6S1	337.0751	CHOS	0.375	1.125	8	0.333
C17H22O7	337.1293	CHO	0.412	1.294	7	0.259
C18H26O6	337.1657	CHO	0.333	1.444	6	0.200
C15H16O7S1	339.0544	CHOS	0.467	1.067	8	0.333
C16H20O6S1	339.0908	CHOS	0.375	1.250	7	0.250
C17H24O7	339.1449	CHO	0.412	1.412	6	0.185
C15H18O7S1	341.0700	CHOS	0.467	1.200	7	0.238
C18H16O7	343.0823	CHO	0.389	0.889	11	0.517
C15H20O7S1	343.0857	CHOS	0.467	1.333	6	0.143
C19H20O6	343.1187	CHO	0.316	1.053	10	0.438
C18H19N1O6	344.1140	CHNO	0.333	1.056	10	0.464
C18H18O7	345.0980	CHO	0.389	1.000	10	0.448
C17H18N2O6	345.1092	CHNO	0.353	1.059	10	0.500
C20H26O5	345.1707	CHO	0.250	1.300	8	0.314
C17H17N1O7	346.0932	CHNO	0.412	1.000	10	0.480
C18H21N1O6	346.1296	CHNO	0.333	1.167	9	0.393
C19H25N1O5	346.1660	CHNO	0.263	1.316	8	0.323
C17H16O8	347.0772	CHO	0.471	0.941	10	0.462
C18H20O7	347.1136	CHO	0.389	1.111	9	0.379
C19H24O6	347.1500	CHO	0.316	1.263	8	0.313
C20H28O5	347.1864	CHO	0.250	1.400	7	0.257

C17H19N1O7	348.1089	CHNO	0.412	1.118	9	0.400
C18H23N1O6	348.1453	CHNO	0.333	1.278	8	0.321
C17H18O8	349.0929	CHO	0.471	1.059	9	0.385
C19H26O6	349.1657	CHO	0.316	1.368	7	0.250
C20H30O5	349.2020	CHO	0.250	1.500	6	0.200
C18H25N1O6	350.1609	CHNO	0.333	1.389	7	0.250
C16H16O7S1	351.0544	CHOS	0.438	1.000	9	0.391
C17H20O8	351.1085	CHO	0.471	1.176	8	0.308
C19H28O6	351.1813	CHO	0.316	1.474	6	0.188
C16H18O7S1	353.0700	CHOS	0.438	1.125	8	0.304
C18H26O7	353.1606	CHO	0.389	1.444	6	0.172
C16H20O7S1	355.0857	CHOS	0.438	1.250	7	0.217
C19H18O7	357.0980	CHO	0.368	0.947	11	0.484
C20H22O6	357.1344	CHO	0.300	1.100	10	0.412
C19H21N1O6	358.1296	CHNO	0.316	1.105	10	0.433
C19H20O7	359.1136	CHO	0.368	1.053	10	0.419
C20H24O6	359.1500	CHO	0.300	1.200	9	0.353
C21H28O5	359.1864	CHO	0.238	1.333	8	0.297
C18H19N1O7	360.1089	CHNO	0.389	1.056	10	0.444
C19H23N1O6	360.1453	CHNO	0.316	1.211	9	0.367
C18H18O8	361.0929	CHO	0.444	1.000	10	0.429
C19H22O7	361.1293	CHO	0.368	1.158	9	0.355
C20H26O6	361.1657	CHO	0.300	1.300	8	0.294
C21H30O5	361.2020	CHO	0.238	1.429	7	0.243
C18H21N1O7	362.1245	CHNO	0.389	1.167	9	0.370
C17H21N3O6	362.1358	CHNO	0.353	1.235	9	0.409
C19H25N1O6	362.1609	CHNO	0.316	1.316	8	0.300
C18H20O6S1	363.0908	CHOS	0.333	1.111	9	0.357
C18H20O8	363.1085	CHO	0.444	1.111	9	0.357
C20H28O6	363.1813	CHO	0.300	1.400	7	0.235
C17H18O7S1	365.0700	CHOS	0.412	1.059	9	0.360
C19H26O7	365.1606	CHO	0.368	1.368	7	0.226
C20H30O6	365.1970	CHO	0.300	1.500	6	0.176
C17H20O7S1	367.0857	CHOS	0.412	1.176	8	0.280
C19H28O5S1	367.1585	CHOS	0.263	1.474	6	0.161
C17H22O7S1	369.1013	CHOS	0.412	1.294	7	0.200
C20H20O7	371.1136	CHO	0.350	1.000	11	0.455
C17H24O7S1	371.1170	CHOS	0.412	1.412	6	0.120
C21H24O6	371.1500	CHO	0.286	1.143	10	0.389
C20H23N1O6	372.1453	CHNO	0.300	1.150	10	0.406
C15H18O9S1	373.0599	CHOS	0.600	1.200	7	0.158
C19H18O8	373.0929	CHO	0.421	0.947	11	0.467
C20H22O7	373.1293	CHO	0.350	1.100	10	0.394
C21H26O6	373.1657	CHO	0.286	1.238	9	0.333
C22H30O5	373.2020	CHO	0.227	1.364	8	0.282
C19H21N1O7	374.1245	CHNO	0.368	1.105	10	0.414
C20H25N1O6	374.1609	CHNO	0.300	1.250	9	0.344
C19H20O8	375.1085	CHO	0.421	1.053	10	0.400
C20H24O7	375.1449	CHO	0.350	1.200	9	0.333
C21H28O6	375.1813	CHO	0.286	1.333	8	0.278

C19H23N1O7	376.1402	CHNO	0.368	1.211	9	0.345
C18H23N3O6	376.1514	CHNO	0.333	1.278	9	0.375
C20H27N1O6	376.1766	CHNO	0.300	1.350	8	0.281
C18H18O7S1	377.0700	CHOS	0.389	1.000	10	0.407
C21H30O6	377.1970	CHO	0.286	1.429	7	0.222
C17H18O8S1	381.0650	CHOS	0.471	1.059	9	0.333
C17H20O8S1	383.0806	CHOS	0.471	1.176	8	0.250
C19H28O6S1	383.1534	CHOS	0.316	1.474	6	0.133
C20H19N1O7	384.1089	CHNO	0.350	0.950	12	0.516
C20H18O8	385.0929	CHO	0.400	0.900	12	0.500
C17H22O8S1	385.0963	CHOS	0.471	1.294	7	0.167
C21H22O7	385.1293	CHO	0.333	1.048	11	0.429
C18H26O7S1	385.1326	CHOS	0.389	1.444	6	0.111
C22H26O6	385.1657	CHO	0.273	1.182	10	0.368
C18H17N3O7	386.0994	CHNO	0.389	0.944	12	0.609
C20H21N1O7	386.1245	CHNO	0.350	1.050	11	0.452
C21H25N1O6	386.1609	CHNO	0.286	1.190	10	0.382
C16H20O9S1	387.0755	CHOS	0.563	1.250	7	0.143
C20H20O8	387.1085	CHO	0.400	1.000	11	0.438
C21H24O7	387.1449	CHO	0.333	1.143	10	0.371
C22H28O6	387.1813	CHO	0.273	1.273	9	0.316
C20H23N1O7	388.1402	CHNO	0.350	1.150	10	0.387
C19H23N3O6	388.1514	CHNO	0.316	1.211	10	0.423
C21H27N1O6	388.1766	CHNO	0.286	1.286	9	0.324
C19H18O9	389.0878	CHO	0.474	0.947	11	0.448
C20H22O8	389.1242	CHO	0.400	1.100	10	0.375
C21H26O7	389.1606	CHO	0.333	1.238	9	0.314
C22H30O6	389.1970	CHO	0.273	1.364	8	0.263
C18H21N3O7	390.1307	CHNO	0.389	1.167	10	0.435
C18H18O8S1	393.0650	CHOS	0.444	1.000	10	0.385
C18H20O8S1	395.0806	CHOS	0.444	1.111	9	0.308
C18H22O8S1	397.0963	CHOS	0.444	1.222	8	0.231
C19H26O7S1	397.1326	CHOS	0.368	1.368	7	0.172
C20H30O6S1	397.1690	CHOS	0.300	1.500	6	0.125
C17H20O9S1	399.0755	CHOS	0.529	1.176	8	0.217
C21H20O8	399.1085	CHO	0.381	0.952	12	0.471
C18H24O8S1	399.1119	CHOS	0.444	1.333	7	0.154
C22H24O7	399.1449	CHO	0.318	1.091	11	0.405
C19H28O7S1	399.1483	CHOS	0.368	1.474	6	0.103
C23H28O6	399.1813	CHO	0.261	1.217	10	0.350
C19H19N3O7	400.1150	CHNO	0.368	1.000	12	0.560
C22H27N1O6	400.1766	CHNO	0.273	1.227	10	0.361
C17H22O9S1	401.0912	CHOS	0.529	1.294	7	0.130
C21H22O8	401.1242	CHO	0.381	1.048	11	0.412
C22H26O7	401.1606	CHO	0.318	1.182	10	0.351
C23H30O6	401.1970	CHO	0.261	1.304	9	0.300
C18H17N3O8	402.0943	CHNO	0.444	0.944	12	0.591
C21H25N1O7	402.1558	CHNO	0.333	1.190	10	0.364
C20H25N3O6	402.1671	CHNO	0.300	1.250	10	0.393
C21H24O8	403.1398	CHO	0.381	1.143	10	0.353

C22H28O7	403.1762	CHO	0.318	1.273	9	0.297
C23H32O6	403.2126	CHO	0.261	1.391	8	0.250
C19H23N3O7	404.1463	CHNO	0.368	1.211	10	0.400
C22H30O7	405.1919	CHO	0.318	1.364	8	0.243
C19H20O8S1	407.0806	CHOS	0.421	1.053	10	0.357
C18H20O9S1	411.0755	CHOS	0.500	1.111	9	0.280
C19H24O8S1	411.1119	CHOS	0.421	1.263	8	0.214
C20H28O7S1	411.1483	CHOS	0.350	1.400	7	0.161
C22H23N1O7	412.1402	CHNO	0.318	1.045	12	0.457
C21H18O9	413.0878	CHO	0.429	0.857	13	0.515
C22H22O8	413.1242	CHO	0.364	1.000	12	0.444
C19H26O8S1	413.1276	CHOS	0.421	1.368	7	0.143
C23H26O7	413.1606	CHO	0.304	1.130	11	0.385
C24H30O6	413.1970	CHO	0.250	1.250	10	0.333
C20H21N3O7	414.1307	CHNO	0.350	1.050	12	0.519
C21H20O9	415.1035	CHO	0.429	0.952	12	0.455
C18H24O9S1	415.1068	CHOS	0.500	1.333	7	0.120
C22H24O8	415.1398	CHO	0.364	1.091	11	0.389
C23H28O7	415.1762	CHO	0.304	1.217	10	0.333
C24H32O6	415.2126	CHO	0.250	1.333	9	0.286
C19H19N3O8	416.1099	CHNO	0.421	1.000	12	0.542
C20H23N3O7	416.1463	CHNO	0.350	1.150	11	0.444
C22H27N1O7	416.1715	CHNO	0.318	1.227	10	0.343
C22H26O8	417.1555	CHO	0.364	1.182	10	0.333
C23H30O7	417.1919	CHO	0.304	1.304	9	0.282
C24H34O6	417.2283	CHO	0.250	1.417	8	0.238
C22H28O8	419.1711	CHO	0.364	1.273	9	0.278
C20H22O8S1	421.0963	CHOS	0.400	1.100	10	0.333
C19H20O9S1	423.0755	CHOS	0.474	1.053	10	0.333
C20H24O8S1	423.1119	CHOS	0.400	1.200	9	0.267
C19H22O9S1	425.0912	CHOS	0.474	1.158	9	0.259
C20H26O8S1	425.1276	CHOS	0.400	1.300	8	0.200
C18H20O10S1	427.0704	CHOS	0.556	1.111	9	0.250
C19H24O9S1	427.1068	CHOS	0.474	1.263	8	0.185
C23H24O8	427.1398	CHO	0.348	1.043	12	0.421
C20H28O8S1	427.1432	CHOS	0.400	1.400	7	0.133
C24H28O7	427.1762	CHO	0.292	1.167	11	0.366
C25H32O6	427.2126	CHO	0.240	1.280	10	0.318
C21H23N3O7	428.1463	CHNO	0.333	1.095	12	0.483
C18H22O10S1	429.0861	CHOS	0.556	1.222	8	0.167
C22H22O9	429.1191	CHO	0.409	1.000	12	0.429
C19H26O9S1	429.1225	CHOS	0.474	1.368	7	0.111
C24H30O7	429.1919	CHO	0.292	1.250	10	0.317
C20H21N3O8	430.1256	CHNO	0.400	1.050	12	0.500
C21H25N3O7	430.1620	CHNO	0.333	1.190	11	0.414
C22H29N3O6	430.1984	CHNO	0.273	1.318	10	0.344
C23H28O8	431.1711	CHO	0.348	1.217	10	0.316
C20H23N3O8	432.1412	CHNO	0.400	1.150	11	0.423
C22H27N1O8	432.1664	CHNO	0.364	1.227	10	0.324
C23H30O8	433.1868	CHO	0.348	1.304	9	0.263

C22H29N1O8	434.1820	CHNO	0.364	1.318	9	0.265
C20H20O9S1	435.0755	CHOS	0.450	1.000	11	0.379
C20H24O9S1	439.1068	CHOS	0.450	1.200	9	0.241
C25H28O7	439.1762	CHO	0.280	1.120	12	0.395
C21H19N3O8	440.1099	CHNO	0.381	0.905	14	0.607
C19H22O10S1	441.0861	CHOS	0.526	1.158	9	0.231
C23H22O9	441.1191	CHO	0.391	0.957	13	0.459
C20H26O9S1	441.1225	CHOS	0.450	1.300	8	0.172
C24H26O8	441.1555	CHO	0.333	1.083	12	0.400
C21H30O8S1	441.1589	CHOS	0.381	1.429	7	0.125
C21H21N3O8	442.1256	CHNO	0.381	1.000	13	0.536
C22H25N3O7	442.1620	CHNO	0.318	1.136	12	0.452
C19H24O10S1	443.1017	CHOS	0.526	1.263	8	0.154
C23H24O9	443.1348	CHO	0.391	1.043	12	0.405
C20H28O9S1	443.1381	CHOS	0.450	1.400	7	0.103
C21H23N3O8	444.1412	CHNO	0.381	1.095	12	0.464
C23H27N1O8	444.1664	CHNO	0.348	1.174	11	0.361
C22H27N3O7	444.1776	CHNO	0.318	1.227	11	0.387
C23H26O9	445.1504	CHO	0.391	1.130	11	0.351
C24H30O8	445.1868	CHO	0.333	1.250	10	0.300
C22H29N3O7	446.1933	CHNO	0.318	1.318	10	0.323
C22H26O8S1	449.1276	CHOS	0.364	1.182	10	0.294
C21H24O9S1	451.1068	CHOS	0.429	1.143	10	0.290
C20H22O10S1	453.0861	CHOS	0.500	1.100	10	0.286
C21H26O9S1	453.1225	CHOS	0.429	1.238	9	0.226
C26H30O7	453.1919	CHO	0.269	1.154	12	0.378
C22H21N3O8	454.1256	CHNO	0.364	0.955	14	0.567
C20H24O10S1	455.1017	CHOS	0.500	1.200	9	0.214
C21H28O9S1	455.1381	CHOS	0.429	1.333	8	0.161
C22H32O8S1	455.1745	CHOS	0.364	1.455	7	0.118
C26H32O7	455.2075	CHO	0.269	1.231	11	0.333
C20H27N1O9S1	456.1334	CHNOS	0.450	1.350	8	0.148
C22H23N3O8	456.1412	CHNO	0.364	1.045	13	0.500
C24H26O9	457.1504	CHO	0.375	1.083	12	0.385
C21H30O9S1	457.1538	CHOS	0.429	1.429	7	0.097
C25H30O8	457.1868	CHO	0.320	1.200	11	0.333
C22H24O9S1	463.1068	CHOS	0.409	1.091	11	0.333
C22H26O9S1	465.1225	CHOS	0.409	1.182	10	0.273
C21H24O10S1	467.1017	CHOS	0.476	1.143	10	0.267
C23H23N3O8	468.1412	CHNO	0.348	1.000	14	0.531
C21H26O10S1	469.1174	CHOS	0.476	1.238	9	0.200
C25H26O9	469.1504	CHO	0.360	1.040	13	0.415
C22H30O9S1	469.1538	CHOS	0.409	1.364	8	0.152
C26H30O8	469.1868	CHO	0.308	1.154	12	0.364
C27H34O7	469.2232	CHO	0.259	1.259	11	0.319
C23H25N3O8	470.1569	CHNO	0.348	1.087	13	0.469
C24H24O10	471.1297	CHO	0.417	1.000	13	0.421
C21H28O10S1	471.1330	CHOS	0.476	1.333	8	0.133
C22H32O9S1	471.1694	CHOS	0.409	1.455	7	0.091
C22H26O10S1	481.1174	CHOS	0.455	1.182	10	0.250

C23H30O9S1	481.1538	CHOS	0.391	1.304	9	0.200
C23H32O9S1	483.1694	CHOS	0.391	1.391	8	0.143
C21H26O11S1	485.1123	CHOS	0.524	1.238	9	0.172
C25H26O10	485.1453	CHO	0.400	1.040	13	0.400
C22H30O10S1	485.1487	CHOS	0.455	1.364	8	0.125
C26H30O9	485.1817	CHO	0.346	1.154	12	0.349
C23H26O10S1	493.1174	CHOS	0.435	1.130	11	0.294
C24H30O9S1	493.1538	CHOS	0.375	1.250	10	0.243
C23H28O10S1	495.1330	CHOS	0.435	1.217	10	0.235
C28H32O8	495.2024	CHO	0.286	1.143	13	0.375
C29H36O7	495.2388	CHO	0.241	1.241	12	0.333
C23H30O10S1	497.1487	CHOS	0.435	1.304	9	0.176
C22H28O11S1	499.1280	CHOS	0.500	1.273	9	0.161
C23H26O11S1	509.1123	CHOS	0.478	1.130	11	0.273
C23H28O11S1	511.1280	CHOS	0.478	1.217	10	0.212
C24H32O10S1	511.1643	CHOS	0.417	1.333	9	0.167
C28H32O9	511.1974	CHO	0.321	1.143	13	0.362
C26H34O9S1	521.1851	CHOS	0.346	1.308	10	0.220
C26H32O11S1	551.1593	CHOS	0.423	1.231	11	0.231
C14H22O3	237.1496	CHO	0.214	1.571	4	0.200
C14H22O4	253.1445	CHO	0.286	1.571	4	0.167
C15H24O4	267.1602	CHO	0.267	1.600	4	0.154
C14H22O5	269.1394	CHO	0.357	1.571	4	0.130
C16H26O4	281.1758	CHO	0.250	1.625	4	0.143
C17H28O4	295.1915	CHO	0.235	1.647	4	0.133
C16H26O5	297.1707	CHO	0.313	1.625	4	0.111
C14H22O5S1	301.1115	CHOS	0.357	1.571	4	0.048
C18H28O4	307.1915	CHO	0.222	1.556	5	0.188
C17H26O5	309.1707	CHO	0.294	1.529	5	0.172
C19H30O4	321.2071	CHO	0.211	1.579	5	0.176
C18H28O5	323.1864	CHO	0.278	1.556	5	0.161
C17H26O6	325.1657	CHO	0.353	1.529	5	0.143
C18H30O5	325.2020	CHO	0.278	1.667	4	0.097
C19H30O5	337.2020	CHO	0.263	1.579	5	0.152
C18H28O6	339.1813	CHO	0.333	1.556	5	0.133
C21H32O5	363.2177	CHO	0.238	1.524	6	0.189
C21H32O6	379.2126	CHO	0.286	1.524	6	0.167
C21H32O6S1	411.1847	CHOS	0.286	1.524	6	0.118
C21H32O7S1	427.1796	CHOS	0.333	1.524	6	0.091
C12H14O3	205.0870	CHO	0.250	1.167	6	0.429
C13H18O2	205.1234	CHO	0.154	1.385	5	0.333
C12H10O4	217.0506	CHO	0.333	0.833	8	0.600
C13H16O3	219.1027	CHO	0.231	1.231	6	0.391
C13H12O4	231.0663	CHO	0.308	0.923	8	0.545
C14H16O3	231.1027	CHO	0.214	1.143	7	0.440
C15H20O2	231.1391	CHO	0.133	1.333	6	0.357
C14H18O3	233.1183	CHO	0.214	1.286	6	0.360
C15H22O2	233.1547	CHO	0.133	1.467	5	0.286
C11H16O6	243.0874	CHO	0.545	1.455	4	0.125
C15H16O3	243.1027	CHO	0.200	1.067	8	0.481

C16H20O2	243.1391	CHO	0.125	1.250	7	0.400
C14H14O4	245.0819	CHO	0.286	1.000	8	0.500
C15H18O3	245.1183	CHO	0.200	1.200	7	0.407
C16H22O2	245.1547	CHO	0.125	1.375	6	0.333
C13H13N1O4	246.0772	CHNO	0.308	1.000	8	0.550
C9H12O6S1	247.0282	CHOS	0.667	1.333	4	0.000
C15H20O3	247.1340	CHO	0.200	1.333	6	0.333
C11H14O7	257.0667	CHO	0.636	1.273	5	0.200
C12H18O6	257.1031	CHO	0.500	1.500	4	0.111
C16H18O3	257.1183	CHO	0.188	1.125	8	0.448
C15H16O4	259.0976	CHO	0.267	1.067	8	0.462
C16H20O3	259.1340	CHO	0.188	1.250	7	0.379
C10H14O6S1	261.0438	CHOS	0.600	1.400	4	0.000
C16H22O3	261.1496	CHO	0.188	1.375	6	0.310
C17H18O3	269.1183	CHO	0.176	1.059	9	0.484
C12H16O7	271.0823	CHO	0.583	1.333	5	0.176
C16H16O4	271.0976	CHO	0.250	1.000	9	0.500
C13H20O6	271.1187	CHO	0.462	1.538	4	0.100
C17H20O3	271.1340	CHO	0.176	1.176	8	0.419
C12H18O7	273.0980	CHO	0.583	1.500	4	0.059
C16H18O4	273.1132	CHO	0.250	1.125	8	0.429
C17H22O3	273.1496	CHO	0.176	1.294	7	0.355
C14H13N1O5	274.0721	CHNO	0.357	0.929	9	0.571
C15H17N1O4	274.1085	CHNO	0.267	1.133	8	0.458
C17H24O3	275.1653	CHO	0.176	1.412	6	0.290
C17H16O4	283.0976	CHO	0.235	0.941	10	0.533
C18H20O3	283.1340	CHO	0.167	1.111	9	0.455
C16H14O5	285.0768	CHO	0.313	0.875	10	0.556
C13H18O7	285.0980	CHO	0.538	1.385	5	0.158
C17H18O4	285.1132	CHO	0.235	1.059	9	0.467
C18H22O3	285.1496	CHO	0.167	1.222	8	0.394
C16H16O5	287.0925	CHO	0.313	1.000	9	0.481
C17H20O4	287.1289	CHO	0.235	1.176	8	0.400
C18H24O3	287.1653	CHO	0.167	1.333	7	0.333
C16H19N1O4	288.1241	CHNO	0.250	1.188	8	0.423
C14H14N2O5	289.0830	CHNO	0.357	1.000	9	0.579
C18H26O3	289.1809	CHO	0.167	1.444	6	0.273
C15H16O4S1	291.0697	CHOS	0.267	1.067	8	0.417
C18H18O4	297.1132	CHO	0.222	1.000	10	0.500
C19H22O3	297.1496	CHO	0.158	1.158	9	0.429
C17H16O5	299.0925	CHO	0.294	0.941	10	0.517
C14H20O7	299.1136	CHO	0.500	1.429	5	0.143
C18H20O4	299.1289	CHO	0.222	1.111	9	0.438
C19H24O3	299.1653	CHO	0.158	1.263	8	0.371
C17H18O5	301.1081	CHO	0.294	1.059	9	0.448
C18H22O4	301.1445	CHO	0.222	1.222	8	0.375
C19H26O3	301.1809	CHO	0.158	1.368	7	0.314
C16H17N1O5	302.1034	CHNO	0.313	1.063	9	0.480
C12H16O7S1	303.0544	CHOS	0.583	1.333	5	0.067
C15H16N2O5	303.0986	CHNO	0.333	1.067	9	0.524

C18H16O5	311.0925	CHO	0.278	0.889	11	0.548
C19H20O4	311.1289	CHO	0.211	1.053	10	0.471
C17H14O6	313.0718	CHO	0.353	0.824	11	0.571
C18H18O5	313.1081	CHO	0.278	1.000	10	0.484
C19H22O4	313.1445	CHO	0.211	1.158	9	0.412
C17H17N1O5	314.1034	CHNO	0.294	1.000	10	0.519
C18H20O5	315.1238	CHO	0.278	1.111	9	0.419
C19H24O4	315.1602	CHO	0.211	1.263	8	0.353
C17H19N1O5	316.1190	CHNO	0.294	1.118	9	0.444
C13H18O7S1	317.0700	CHOS	0.538	1.385	5	0.059
C16H18N2O5	317.1143	CHNO	0.313	1.125	9	0.478
C13H20O7S1	319.0857	CHOS	0.538	1.538	4	-0.059
C20H22O4	325.1445	CHO	0.200	1.100	10	0.444
C18H16O6	327.0874	CHO	0.333	0.889	11	0.533
C19H20O5	327.1238	CHO	0.263	1.053	10	0.455
C20H24O4	327.1602	CHO	0.200	1.200	9	0.389
C20H26O4	329.1758	CHO	0.200	1.300	8	0.333
C18H21N1O5	330.1347	CHNO	0.278	1.167	9	0.414
C14H20O7S1	331.0857	CHOS	0.500	1.429	5	0.053
C17H20N2O5	331.1299	CHNO	0.294	1.176	9	0.440
C13H20O8S1	335.0806	CHOS	0.615	1.538	4	-0.125
C20H20O5	339.1238	CHO	0.250	1.000	11	0.486
C19H18O6	341.1031	CHO	0.316	0.947	11	0.500
C20H22O5	341.1394	CHO	0.250	1.100	10	0.429
C21H26O4	341.1758	CHO	0.190	1.238	9	0.368
C20H24O5	343.1551	CHO	0.250	1.200	9	0.371
C21H28O4	343.1915	CHO	0.190	1.333	8	0.316
C19H23N1O5	344.1503	CHNO	0.263	1.211	9	0.387
C14H18O8S1	345.0650	CHOS	0.571	1.286	6	0.111
C15H22O7S1	345.1013	CHOS	0.467	1.467	5	0.048
C14H20O8S1	347.0806	CHOS	0.571	1.429	5	0.000
C21H22O5	353.1394	CHO	0.238	1.048	11	0.459
C22H26O4	353.1758	CHO	0.182	1.182	10	0.400
C18H17N3O5	354.1095	CHNO	0.278	0.944	12	0.640
C20H20O6	355.1187	CHO	0.300	1.000	11	0.471
C21H24O5	355.1551	CHO	0.238	1.143	10	0.405
C22H28O4	355.1915	CHO	0.182	1.273	9	0.350
C20H23N1O5	356.1503	CHNO	0.250	1.150	10	0.424
C21H26O5	357.1707	CHO	0.238	1.238	9	0.351
C17H17N3O6	358.1045	CHNO	0.353	1.000	11	0.591
C20H25N1O5	358.1660	CHNO	0.250	1.250	9	0.364
C15H20O8S1	359.0806	CHOS	0.533	1.333	6	0.100
C16H24O7S1	359.1170	CHOS	0.438	1.500	5	0.043
C15H22O8S1	361.0963	CHOS	0.533	1.467	5	0.000
C21H20O6	367.1187	CHO	0.286	0.952	12	0.500
C22H24O5	367.1551	CHO	0.227	1.091	11	0.436
C23H28O4	367.1915	CHO	0.174	1.217	10	0.381
C19H19N3O5	368.1252	CHNO	0.263	1.000	12	0.593
C21H22O6	369.1344	CHO	0.286	1.048	11	0.444
C22H26O5	369.1707	CHO	0.227	1.182	10	0.385

C18H17N3O6	370.1045	CHNO	0.333	0.944	12	0.625
C22H28O5	371.1864	CHO	0.227	1.273	9	0.333
C23H32O4	371.2228	CHO	0.174	1.391	8	0.286
C18H19N3O6	372.1201	CHNO	0.333	1.056	11	0.542
C15H20O9S1	375.0755	CHOS	0.600	1.333	6	0.053
C16H24O8S1	375.1119	CHOS	0.500	1.500	5	0.000
C23H26O5	381.1707	CHO	0.217	1.130	11	0.415
C21H21N1O6	382.1296	CHNO	0.286	1.000	12	0.500
C22H24O6	383.1500	CHO	0.273	1.091	11	0.421
C23H28O5	383.1864	CHO	0.217	1.217	10	0.366
C19H19N3O6	384.1201	CHNO	0.316	1.000	12	0.577
C20H23N3O5	384.1565	CHNO	0.250	1.150	11	0.483
C23H30O5	385.2020	CHO	0.217	1.304	9	0.317
C19H21N3O6	386.1358	CHNO	0.316	1.105	11	0.500
C17H24O8S1	387.1119	CHOS	0.471	1.412	6	0.083
C23H24O6	395.1500	CHO	0.261	1.043	12	0.450
C20H19N3O6	396.1201	CHNO	0.300	0.950	13	0.607
C21H23N3O5	396.1565	CHNO	0.238	1.095	12	0.516
C23H26O6	397.1657	CHO	0.261	1.130	11	0.400
C24H30O5	397.2020	CHO	0.208	1.250	10	0.349
C20H21N3O6	398.1358	CHNO	0.300	1.050	12	0.536
C22H25N1O6	398.1609	CHNO	0.273	1.136	11	0.417
C24H32O5	399.2177	CHO	0.208	1.333	9	0.302
C20H23N3O6	400.1514	CHNO	0.300	1.150	11	0.464
C18H26O8S1	401.1276	CHOS	0.444	1.444	6	0.077
C17H24O9S1	403.1068	CHOS	0.529	1.412	6	0.043
C21H21N3O6	410.1358	CHNO	0.286	1.000	13	0.567
C24H28O6	411.1813	CHO	0.250	1.167	11	0.381
C20H19N3O7	412.1150	CHNO	0.350	0.950	13	0.593
C21H23N3O6	412.1514	CHNO	0.286	1.095	12	0.500
C21H25N3O6	414.1671	CHNO	0.286	1.190	11	0.433
C19H28O8S1	415.1432	CHOS	0.421	1.474	6	0.071
C17H22O10S1	417.0861	CHOS	0.588	1.294	7	0.091
C18H26O9S1	417.1225	CHOS	0.500	1.444	6	0.040
C17H24O10S1	419.1017	CHOS	0.588	1.412	6	0.000
C22H21N3O6	422.1358	CHNO	0.273	0.955	14	0.594
C21H19N3O7	424.1150	CHNO	0.333	0.905	14	0.621
C25H30O6	425.1970	CHO	0.240	1.200	11	0.364
C21H21N3O7	426.1307	CHNO	0.333	1.000	13	0.552
C20H30O8S1	429.1589	CHOS	0.400	1.500	6	0.067
C19H28O9S1	431.1381	CHOS	0.474	1.474	6	0.037
C18H26O10S1	433.1174	CHOS	0.556	1.444	6	0.000
C22H21N3O7	438.1307	CHNO	0.318	0.955	14	0.581
C22H23N3O7	440.1463	CHNO	0.318	1.045	13	0.516
C19H26O10S1	445.1174	CHOS	0.526	1.368	7	0.077
C20H30O9S1	445.1538	CHOS	0.450	1.500	6	0.034
C19H28O10S1	447.1330	CHOS	0.526	1.474	6	0.000
C23H23N3O7	452.1463	CHNO	0.304	1.000	14	0.545
C19H24O11S1	459.0967	CHOS	0.579	1.263	8	0.120
C20H28O10S1	459.1330	CHOS	0.500	1.400	7	0.071

C19H26O11S1	461.1123	CHOS	0.579	1.368	7	0.040
C20H30O10S1	461.1487	CHOS	0.500	1.500	6	0.000
C19H28O11S1	463.1280	CHOS	0.579	1.474	6	-0.040
C20H26O11S1	473.1123	CHOS	0.550	1.300	8	0.111
C21H30O10S1	473.1487	CHOS	0.476	1.429	7	0.067
C24H23N3O8	480.1412	CHNO	0.333	0.958	15	0.559
C22H32O10S1	487.1643	CHOS	0.455	1.455	7	0.063
C21H30O11S1	489.1436	CHOS	0.524	1.429	7	0.034
C25H25N3O8	494.1569	CHNO	0.320	1.000	15	0.528
C18H15N3O7	384.0837	CHNO	0.389	0.833	13	0.696
C20H17N3O6	394.1045	CHNO	0.300	0.850	14	0.679
C19H17N3O7	398.0994	CHNO	0.368	0.895	13	0.640
C20H17N3O7	410.0994	CHNO	0.350	0.850	14	0.667
C21H17N3O8	438.0943	CHNO	0.381	0.810	15	0.679
C14H22O2	221.1547	CHO	0.143	1.571	4	0.231
C11H18O5	229.1081	CHO	0.455	1.636	3	0.059
C12H20O5	243.1238	CHO	0.417	1.667	3	0.053
C11H18O6	245.1031	CHO	0.545	1.636	3	0.000
C14H24O5	271.1551	CHO	0.357	1.714	3	0.043
C13H22O6	273.1344	CHO	0.462	1.692	3	0.000
C14H22O6	285.1344	CHO	0.429	1.571	4	0.091
C15H24O6	299.1500	CHO	0.400	1.600	4	0.083
C16H28O5	299.1864	CHO	0.313	1.750	3	0.037
C14H22O7	301.1293	CHO	0.500	1.571	4	0.048
C15H24O7	315.1449	CHO	0.467	1.600	4	0.043
C14H22O6S1	317.1064	CHOS	0.429	1.571	4	0.000
C15H26O7	317.1606	CHO	0.467	1.733	3	-0.043
C14H22O7S1	333.1013	CHOS	0.500	1.571	4	-0.053
C15H24O7S1	347.1170	CHOS	0.467	1.600	4	-0.048
C14H22O8S1	349.0963	CHOS	0.571	1.571	4	-0.111
C17H26O7S1	373.1326	CHOS	0.412	1.529	5	0.040
C17H28O7S1	375.1483	CHOS	0.412	1.647	4	-0.040
C16H26O8S1	377.1276	CHOS	0.500	1.625	4	-0.091
C18H28O7S1	387.1483	CHOS	0.389	1.556	5	0.037
C21H34O7	397.2232	CHO	0.333	1.619	5	0.086
C19H30O7S1	401.1639	CHOS	0.368	1.579	5	0.034
C18H28O8S1	403.1432	CHOS	0.444	1.556	5	0.000
C18H28O9S1	419.1381	CHOS	0.500	1.556	5	-0.040
C21H32O8S1	443.1745	CHOS	0.381	1.524	6	0.063
C20H32O9S1	447.1694	CHOS	0.450	1.600	5	-0.034
C21H32O10S1	475.1643	CHOS	0.476	1.524	6	0.000

Table S3. Formula, *m/z*, element class, O/C, H/C, DEB, and AI_{mod} of the 394 deep-sea-distributed molecules molecular formulas existed during the 90-day incubation.

Formula	m/z	Class	O/C	H/C	DBE	AI_{mod}
C11H12O4	207.0663	CHO	0.2857	1.2857	6	0.3333
C14H18O4	249.1132	CHO	0.2000	1.4667	5	0.2593
C15H22O3	249.1496	CHO	0.3846	1.2308	6	0.3333
C13H16O5	251.0925	CHO	0.2857	1.4286	5	0.2500
C14H20O4	251.1289	CHO	0.3846	1.3846	5	0.2381
C13H18O5	253.1081	CHO	0.3846	1.5385	4	0.1429
C13H20O5	255.1238	CHO	0.2667	1.2000	7	0.3846
C15H18O4	261.1132	CHO	0.2857	1.2143	7	0.4091
C14H17N1O4	262.1085	CHON	0.3571	1.1429	7	0.3913
C14H16O5	263.0925	CHO	0.2667	1.3333	6	0.3077
C15H20O4	263.1289	CHO	0.1875	1.5000	5	0.2414
C16H24O3	263.1653	CHO	0.3846	1.1538	7	0.4211
C13H15N1O5	264.0877	CHON	0.4615	1.0769	7	0.4000
C13H14O6	265.0718	CHO	0.3571	1.2857	6	0.3043
C14H18O5	265.1081	CHO	0.3846	1.3077	6	0.3158
C13H17N1O5	266.1034	CHON	0.4615	1.2308	6	0.3000
C13H16O6	267.0874	CHO	0.3571	1.4286	5	0.2174
C14H20O5	267.1238	CHO	0.3846	1.4615	5	0.2105
C13H19N1O5	268.119	CHON	0.3333	1.0667	8	0.4400
C15H16O5	275.0925	CHO	0.2500	1.2500	7	0.3571
C16H20O4	275.1289	CHO	0.4286	1.0000	8	0.4545
C14H14O6	277.0718	CHO	0.3333	1.2000	7	0.3600
C15H18O5	277.1081	CHO	0.2500	1.3750	6	0.2857
C16H22O4	277.1445	CHO	0.3571	1.2143	7	0.3810
C14H17N1O5	278.1034	CHON	0.4286	1.1429	7	0.3636
C14H16O6	279.0874	CHO	0.3333	1.3333	6	0.2800
C15H20O5	279.1238	CHO	0.2500	1.5000	5	0.2143
C16H24O4	279.1602	CHO	0.3571	1.3571	6	0.2857
C14H19N1O5	280.119	CHON	0.5385	1.0769	7	0.3684
C13H14O7	281.0667	CHO	0.4286	1.2857	6	0.2727
C14H18O6	281.1031	CHO	0.3333	1.4667	5	0.2000
C15H22O5	281.1394	CHO	0.3571	1.5000	5	0.1905
C14H21N1O5	282.1347	CHON	0.5385	1.2308	6	0.2632
C13H16O7	283.0823	CHO	0.4286	1.4286	5	0.1818
C14H20O6	283.1187	CHO	0.4000	0.9333	9	0.5000
C15H14O6	289.0718	CHO	0.3125	1.1250	8	0.4074
C16H18O5	289.1081	CHO	0.2353	1.2941	7	0.3333
C17H22O4	289.1445	CHO	0.3333	1.1333	8	0.4348
C15H17N1O5	290.1034	CHON	0.4000	1.0667	8	0.4167
C15H16O6	291.0874	CHO	0.3125	1.2500	7	0.3333
C16H20O5	291.1238	CHO	0.2353	1.4118	6	0.2667
C17H24O4	291.1602	CHO	0.4286	1.0714	8	0.4500
C14H15N1O6	292.0827	CHON	0.3333	1.2667	7	0.3478
C15H19N1O5	292.119	CHON	0.5000	1.0000	8	0.4286
C14H14O7	293.0667	CHO	0.4000	1.2000	7	0.3333
C15H18O6	293.1031	CHO	0.3125	1.3750	6	0.2593
C16H22O5	293.1394	CHO	0.4286	1.2143	7	0.3500

C14H17N1O6	294.0983	CHON	0.3333	1.4000	6	0.2609
C15H21N1O5	294.1347	CHON	0.5000	1.1429	7	0.3333
C14H16O7	295.0823	CHO	0.4000	1.3333	6	0.2500
C15H20O6	295.1187	CHO	0.3125	1.5000	5	0.1852
C16H24O5	295.1551	CHO	0.6154	1.0769	7	0.3333
C13H14O8	297.0616	CHO	0.4000	1.4667	5	0.1667
C15H22O6	297.1344	CHO	0.3750	1.0000	9	0.4615
C16H16O6	303.0874	CHO	0.2941	1.1765	8	0.3793
C17H20O5	303.1238	CHO	0.2222	1.3333	7	0.3125
C18H24O4	303.1602	CHO	0.3125	1.1875	8	0.4000
C16H19N1O5	304.119	CHON	0.4667	0.9333	9	0.4783
C15H14O7	305.0667	CHO	0.3750	1.1250	8	0.3846
C16H18O6	305.1031	CHO	0.2222	1.4444	6	0.2500
C18H26O4	305.1758	CHO	0.4000	1.1333	8	0.4091
C15H17N1O6	306.0983	CHON	0.3125	1.3125	7	0.3200
C16H21N1O5	306.1347	CHON	0.5714	0.8571	9	0.5000
C14H12O8	307.0459	CHO	0.4667	1.0667	8	0.3913
C15H16O7	307.0823	CHO	0.3750	1.2500	7	0.3077
C16H20O6	307.1187	CHO	0.2941	1.4118	6	0.2414
C17H24O5	307.1551	CHO	0.3125	1.4375	6	0.2400
C16H23N1O5	308.1503	CHON	0.5714	1.0000	8	0.4000
C14H14O8	309.0616	CHO	0.4667	1.2000	7	0.3043
C15H18O7	309.098	CHO	0.3750	1.3750	6	0.2308
C16H22O6	309.1344	CHO	0.4000	1.4000	6	0.2273
C15H21N1O6	310.1296	CHON	0.3750	1.5000	5	0.1538
C16H24O6	311.15	CHO	0.3529	0.9412	10	0.5000
C17H16O6	315.0874	CHO	0.4286	1.4286	5	0.1000
C14H20O6S1	315.0908	CHOS	0.3529	1.0588	9	0.4286
C17H18O6	317.1031	CHO	0.2778	1.2222	8	0.3548
C18H22O5	317.1394	CHO	0.2105	1.3684	7	0.2941
C19H26O4	317.1758	CHO	0.3750	1.0625	9	0.4583
C16H17N1O6	318.0983	CHON	0.2941	1.2353	8	0.3704
C17H21N1O5	318.1347	CHON	0.4375	1.0000	9	0.4400
C16H16O7	319.0823	CHO	0.3529	1.1765	8	0.3571
C17H20O6	319.1187	CHO	0.3125	1.2500	8	0.3913
C16H20N2O5	319.1299	CHON	0.2778	1.3333	7	0.2903
C18H24O5	319.1551	CHO	0.2105	1.4737	6	0.2353
C19H28O4	319.1915	CHO	0.3750	1.1875	8	0.3750
C16H19N1O6	320.114	CHON	0.2941	1.3529	7	0.2963
C17H23N1O5	320.1503	CHON	0.5333	0.9333	9	0.4545
C15H14O8	321.0616	CHO	0.4375	1.1250	8	0.3600
C16H18O7	321.098	CHO	0.3529	1.2941	7	0.2857
C17H22O6	321.1344	CHO	0.2778	1.4444	6	0.2258
C18H26O5	321.1707	CHO	0.3750	1.3125	7	0.2917
C16H21N1O6	322.1296	CHON	0.4375	1.2500	7	0.2800
C16H20O7	323.1136	CHO	0.3529	1.4118	6	0.2143
C17H24O6	323.15	CHO	0.3333	1.0000	10	0.4667
C18H18O6	329.1031	CHO	0.4118	0.9412	10	0.4815
C17H16O7	331.0823	CHO	0.3333	1.1111	9	0.4000
C18H20O6	331.1187	CHO	0.2632	1.2632	8	0.3333

C19H24O5	331.1551	CHO	0.2000	1.4000	7	0.2778
C20H28O4	331.1915	CHO	0.3529	1.1176	9	0.4231
C17H19N1O6	332.114	CHON	0.5000	0.8750	10	0.5000
C16H14O8	333.0616	CHO	0.4118	1.0588	9	0.4074
C17H18O7	333.098	CHO	0.3333	1.2222	8	0.3333
C18H22O6	333.1344	CHO	0.2941	1.2941	8	0.3600
C17H22N2O5	333.1456	CHON	0.2632	1.3684	7	0.2727
C19H26O5	333.1707	CHO	0.3529	1.2353	8	0.3462
C17H21N1O6	334.1296	CHON	0.2778	1.3889	7	0.2759
C18H25N1O5	334.166	CHON	0.5000	1.0000	9	0.4167
C16H16O8	335.0772	CHO	0.4118	1.1765	8	0.3333
C17H20O7	335.1136	CHO	0.3333	1.3333	7	0.2667
C18H24O6	335.15	CHO	0.2632	1.4737	6	0.2121
C19H28O5	335.1864	CHO	0.3529	1.3529	7	0.2692
C17H23N1O6	336.1453	CHON	0.4118	1.2941	7	0.2593
C17H22O7	337.1293	CHO	0.3333	1.4444	6	0.2000
C18H26O6	337.1657	CHO	0.4118	1.4118	6	0.1852
C17H24O7	339.1449	CHO	0.3889	0.8889	11	0.5172
C18H16O7	343.0823	CHO	0.4667	1.3333	6	0.1429
C15H20O7S1	343.0857	CHOS	0.3158	1.0526	10	0.4375
C19H20O6	343.1187	CHO	0.3333	1.0556	10	0.4643
C18H19N1O6	344.114	CHON	0.3889	1.0000	10	0.4483
C18H18O7	345.098	CHO	0.3529	1.0588	10	0.5000
C17H18N2O6	345.1092	CHON	0.2500	1.3000	8	0.3143
C20H26O5	345.1707	CHO	0.4118	1.0000	10	0.4800
C17H17N1O7	346.0932	CHON	0.3333	1.1667	9	0.3929
C18H21N1O6	346.1296	CHON	0.2632	1.3158	8	0.3226
C19H25N1O5	346.166	CHON	0.4706	0.9412	10	0.4615
C17H16O8	347.0772	CHO	0.3889	1.1111	9	0.3793
C18H20O7	347.1136	CHO	0.3158	1.2632	8	0.3125
C19H24O6	347.15	CHO	0.2500	1.4000	7	0.2571
C20H28O5	347.1864	CHO	0.4118	1.1176	9	0.4000
C17H19N1O7	348.1089	CHON	0.3333	1.2778	8	0.3214
C18H23N1O6	348.1453	CHON	0.4706	1.0588	9	0.3846
C17H18O8	349.0929	CHO	0.3158	1.3684	7	0.2500
C19H26O6	349.1657	CHO	0.2500	1.5000	6	0.2000
C20H30O5	349.202	CHO	0.3333	1.3889	7	0.2500
C18H25N1O6	350.1609	CHON	0.4706	1.1765	8	0.3077
C17H20O8	351.1085	CHO	0.3158	1.4737	6	0.1875
C19H28O6	351.1813	CHO	0.3889	1.4444	6	0.1724
C18H26O7	353.1606	CHO	0.3684	0.9474	11	0.4839
C19H18O7	357.098	CHO	0.3000	1.1000	10	0.4118
C20H22O6	357.1344	CHO	0.3158	1.1053	10	0.4333
C19H21N1O6	358.1296	CHON	0.3684	1.0526	10	0.4194
C19H20O7	359.1136	CHO	0.3000	1.2000	9	0.3529
C20H24O6	359.15	CHO	0.2381	1.3333	8	0.2973
C21H28O5	359.1864	CHO	0.3889	1.0556	10	0.4444
C18H19N1O7	360.1089	CHON	0.3158	1.2105	9	0.3667
C19H23N1O6	360.1453	CHON	0.4444	1.0000	10	0.4286
C18H18O8	361.0929	CHO	0.3684	1.1579	9	0.3548

C19H22O7	361.1293	CHO	0.3000	1.3000	8	0.2941
C20H26O6	361.1657	CHO	0.2381	1.4286	7	0.2432
C21H30O5	361.202	CHO	0.3889	1.1667	9	0.3704
C18H21N1O7	362.1245	CHON	0.3529	1.2353	9	0.4091
C17H21N3O6	362.1358	CHON	0.3158	1.3158	8	0.3000
C19H25N1O6	362.1609	CHON	0.4444	1.1111	9	0.3571
C18H20O8	363.1085	CHO	0.3000	1.4000	7	0.2353
C20H28O6	363.1813	CHO	0.3684	1.3684	7	0.2258
C19H26O7	365.1606	CHO	0.3000	1.5000	6	0.1765
C20H30O6	365.197	CHO	0.3500	1.0000	11	0.4545
C20H20O7	371.1136	CHO	0.2857	1.1429	10	0.3889
C21H24O6	371.15	CHO	0.3000	1.1500	10	0.4063
C20H23N1O6	372.1453	CHON	0.6000	1.2000	7	0.1579
C15H18O9S1	373.0599	CHOS	0.4211	0.9474	11	0.4667
C19H18O8	373.0929	CHO	0.3500	1.1000	10	0.3939
C20H22O7	373.1293	CHO	0.2857	1.2381	9	0.3333
C21H26O6	373.1657	CHO	0.2273	1.3636	8	0.2821
C22H30O5	373.202	CHO	0.3684	1.1053	10	0.4138
C19H21N1O7	374.1245	CHON	0.3000	1.2500	9	0.3438
C20H25N1O6	374.1609	CHON	0.4211	1.0526	10	0.4000
C19H20O8	375.1085	CHO	0.3500	1.2000	9	0.3333
C20H24O7	375.1449	CHO	0.2857	1.3333	8	0.2778
C21H28O6	375.1813	CHO	0.3684	1.2105	9	0.3448
C19H23N1O7	376.1402	CHON	0.3333	1.2778	9	0.3750
C18H23N3O6	376.1514	CHON	0.3000	1.3500	8	0.2813
C20H27N1O6	376.1766	CHON	0.2857	1.4286	7	0.2222
C21H30O6	377.197	CHO	0.4706	1.1765	8	0.2500
C17H20O8S1	383.0806	CHOS	0.3500	0.9500	12	0.5161
C20H19N1O7	384.1089	CHON	0.4000	0.9000	12	0.5000
C20H18O8	385.0929	CHO	0.4706	1.2941	7	0.1667
C17H22O8S1	385.0963	CHOS	0.3333	1.0476	11	0.4286
C21H22O7	385.1293	CHO	0.2727	1.1818	10	0.3684
C22H26O6	385.1657	CHO	0.3889	0.9444	12	0.6087
C18H17N3O7	386.0994	CHON	0.3500	1.0500	11	0.4516
C20H21N1O7	386.1245	CHON	0.2857	1.1905	10	0.3824
C21H25N1O6	386.1609	CHON	0.5625	1.2500	7	0.1429
C16H20O9S1	387.0755	CHOS	0.4000	1.0000	11	0.4375
C20H20O8	387.1085	CHO	0.3333	1.1429	10	0.3714
C21H24O7	387.1449	CHO	0.2727	1.2727	9	0.3158
C22H28O6	387.1813	CHO	0.3500	1.1500	10	0.3871
C20H23N1O7	388.1402	CHON	0.3158	1.2105	10	0.4231
C19H23N3O6	388.1514	CHON	0.2857	1.2857	9	0.3235
C21H27N1O6	388.1766	CHON	0.4737	0.9474	11	0.4483
C19H18O9	389.0878	CHO	0.4000	1.1000	10	0.3750
C20H22O8	389.1242	CHO	0.3333	1.2381	9	0.3143
C21H26O7	389.1606	CHO	0.2727	1.3636	8	0.2632
C22H30O6	389.197	CHO	0.3889	1.1667	10	0.4348
C18H21N3O7	390.1307	CHON	0.5294	1.1765	8	0.2174
C17H20O9S1	399.0755	CHOS	0.3810	0.9524	12	0.4706
C21H20O8	399.1085	CHO	0.3182	1.0909	11	0.4054

C22H24O7	399.1449	CHO	0.2609	1.2174	10	0.3500
C23H28O6	399.1813	CHO	0.3684	1.0000	12	0.5600
C19H19N3O7	400.115	CHON	0.5294	1.2941	7	0.1304
C17H22O9S1	401.0912	CHOS	0.3810	1.0476	11	0.4118
C21H22O8	401.1242	CHO	0.3182	1.1818	10	0.3514
C22H26O7	401.1606	CHO	0.2609	1.3043	9	0.3000
C23H30O6	401.197	CHO	0.4444	0.9444	12	0.5909
C18H17N3O8	402.0943	CHON	0.3333	1.1905	10	0.3636
C21H25N1O7	402.1558	CHON	0.3000	1.2500	10	0.3929
C20H25N3O6	402.1671	CHON	0.3810	1.1429	10	0.3529
C21H24O8	403.1398	CHO	0.3182	1.2727	9	0.2973
C22H28O7	403.1762	CHO	0.2609	1.3913	8	0.2500
C23H32O6	403.2126	CHO	0.3684	1.2105	10	0.4000
C19H23N3O7	404.1463	CHON	0.3182	1.3636	8	0.2432
C22H30O7	405.1919	CHO	0.5000	1.1111	9	0.2800
C18H20O9S1	411.0755	CHOS	0.3636	1.0000	12	0.4444
C22H22O8	413.1242	CHO	0.3043	1.1304	11	0.3846
C23H26O7	413.1606	CHO	0.2500	1.2500	10	0.3333
C24H30O6	413.197	CHO	0.3500	1.0500	12	0.5185
C20H21N3O7	414.1307	CHON	0.4286	0.9524	12	0.4545
C21H20O9	415.1035	CHO	0.5000	1.3333	7	0.1200
C18H24O9S1	415.1068	CHOS	0.3636	1.0909	11	0.3889
C22H24O8	415.1398	CHO	0.3043	1.2174	10	0.3333
C23H28O7	415.1762	CHO	0.2500	1.3333	9	0.2857
C24H32O6	415.2126	CHO	0.4211	1.0000	12	0.5417
C19H19N3O8	416.1099	CHON	0.3500	1.1500	11	0.4444
C20H23N3O7	416.1463	CHON	0.3182	1.2273	10	0.3429
C22H27N1O7	416.1715	CHON	0.3636	1.1818	10	0.3333
C22H26O8	417.1555	CHO	0.3043	1.3043	9	0.2821
C23H30O7	417.1919	CHO	0.2500	1.4167	8	0.2381
C24H34O6	417.2283	CHO	0.3636	1.2727	9	0.2778
C22H28O8	419.1711	CHO	0.5556	1.1111	9	0.2500
C18H20O10S1	427.0704	CHOS	0.4737	1.2632	8	0.1852
C19H24O9S1	427.1068	CHOS	0.2917	1.1667	11	0.3659
C24H28O7	427.1762	CHO	0.2400	1.2800	10	0.3182
C25H32O6	427.2126	CHO	0.3333	1.0952	12	0.4828
C21H23N3O7	428.1463	CHON	0.5556	1.2222	8	0.1667
C18H22O10S1	429.0861	CHOS	0.4091	1.0000	12	0.4286
C22H22O9	429.1191	CHO	0.4737	1.3684	7	0.1111
C19H26O9S1	429.1225	CHOS	0.2917	1.2500	10	0.3171
C24H30O7	429.1919	CHO	0.4000	1.0500	12	0.5000
C20H21N3O8	430.1256	CHON	0.3333	1.1905	11	0.4138
C21H25N3O7	430.162	CHON	0.2727	1.3182	10	0.3438
C22H29N3O6	430.1984	CHON	0.3478	1.2174	10	0.3158
C23H28O8	431.1711	CHO	0.4000	1.1500	11	0.4231
C20H23N3O8	432.1412	CHON	0.3636	1.2273	10	0.3235
C22H27N1O8	432.1664	CHON	0.3478	1.3043	9	0.2632
C23H30O8	433.1868	CHO	0.3636	1.3182	9	0.2647
C22H29N1O8	434.182	CHON	0.5263	1.1579	9	0.2308
C19H22O10S1	441.0861	CHOS	0.3810	1.0000	13	0.5357

C21H21N3O8	442.1256	CHON	0.3182	1.1364	12	0.4516
C22H25N3O7	442.162	CHON	0.5263	1.2632	8	0.1538
C19H24O10S1	443.1017	CHOS	0.3913	1.0435	12	0.4054
C23H24O9	443.1348	CHO	0.4500	1.4000	7	0.1034
C20H28O9S1	443.1381	CHOS	0.3810	1.0952	12	0.4643
C21H23N3O8	444.1412	CHON	0.3478	1.1739	11	0.3611
C23H27N1O8	444.1664	CHON	0.3182	1.2273	11	0.3871
C22H27N3O7	444.1776	CHON	0.3913	1.1304	11	0.3514
C23H26O9	445.1504	CHO	0.3333	1.2500	10	0.3000
C24H30O8	445.1868	CHO	0.3182	1.3182	10	0.3226
C22H29N3O7	446.1933	CHON	0.5000	1.2000	9	0.2143
C20H24O10S1	455.1017	CHOS	0.3636	1.0455	13	0.5000
C22H23N3O8	456.1412	CHON	0.3750	1.0833	12	0.3846
C24H26O9	457.1504	CHO	0.3200	1.2000	11	0.3333
C25H30O8	457.1868	CHO	0.4762	1.2381	9	0.2000
C21H26O10S1	469.1174	CHOS	0.3478	1.0870	13	0.4688
C23H25N3O8	470.1569	CHON	0.4167	1.0000	13	0.4211
C24H24O10	471.1297	CHO	0.4762	1.3333	8	0.1333
C21H28O10S1	471.133	CHOS	0.5238	1.2381	9	0.1724
C21H26O11S1	485.1123	CHOS	0.4545	1.3636	8	0.1250
C22H30O10S1	485.1487	CHOS	0.3462	1.1538	12	0.3488
C26H30O9	485.1817	CHO	0.5000	1.2727	9	0.1613
C22H28O11S1	499.128	CHOS	0.2857	1.5714	4	0.1667
C14H22O4	253.1445	CHO	0.2667	1.6000	4	0.1538
C15H24O4	267.1602	CHO	0.3571	1.5714	4	0.1304
C14H22O5	269.1394	CHO	0.3125	1.6250	4	0.1111
C16H26O5	297.1707	CHO	0.2222	1.5556	5	0.1875
C18H28O4	307.1915	CHO	0.2941	1.5294	5	0.1724
C17H26O5	309.1707	CHO	0.2105	1.5789	5	0.1765
C19H30O4	321.2071	CHO	0.2778	1.5556	5	0.1613
C18H28O5	323.1864	CHO	0.3529	1.5294	5	0.1429
C17H26O6	325.1657	CHO	0.2778	1.6667	4	0.0968
C18H30O5	325.202	CHO	0.2632	1.5789	5	0.1515
C19H30O5	337.202	CHO	0.3333	1.5556	5	0.1333
C18H28O6	339.1813	CHO	0.2381	1.5238	6	0.1892
C21H32O5	363.2177	CHO	0.2857	1.5238	6	0.1667
C21H32O6	379.2126	CHO	0.2667	1.0667	8	0.4615
C15H16O4	259.0976	CHO	0.1875	1.3750	6	0.3103
C16H22O3	261.1496	CHO	0.1765	1.0588	9	0.4839
C17H18O3	269.1183	CHO	0.5833	1.3333	5	0.1765
C12H16O7	271.0823	CHO	0.2500	1.0000	9	0.5000
C16H16O4	271.0976	CHO	0.4615	1.5385	4	0.1000
C13H20O6	271.1187	CHO	0.1765	1.1765	8	0.4194
C17H20O3	271.134	CHO	0.5833	1.5000	4	0.0588
C12H18O7	273.098	CHO	0.2500	1.1250	8	0.4286
C16H18O4	273.1132	CHO	0.1765	1.2941	7	0.3548
C17H22O3	273.1496	CHO	0.2667	1.1333	8	0.4583
C15H17N1O4	274.1085	CHON	0.1765	1.4118	6	0.2903
C17H24O3	275.1653	CHO	0.2353	0.9412	10	0.5333
C17H16O4	283.0976	CHO	0.5385	1.3846	5	0.1579

C13H18O7	285.098	CHO	0.2353	1.0588	9	0.4667
C17H18O4	285.1132	CHO	0.3125	1.0000	9	0.4815
C16H16O5	287.0925	CHO	0.2353	1.1765	8	0.4000
C17H20O4	287.1289	CHO	0.1667	1.3333	7	0.3333
C18H24O3	287.1653	CHO	0.2500	1.1875	8	0.4231
C16H19N1O4	288.1241	CHON	0.3571	1.0000	9	0.5789
C14H14N2O5	289.083	CHON	0.1667	1.4444	6	0.2727
C18H26O3	289.1809	CHO	0.2222	1.0000	10	0.5000
C18H18O4	297.1132	CHO	0.2941	0.9412	10	0.5172
C17H16O5	299.0925	CHO	0.5000	1.4286	5	0.1429
C14H20O7	299.1136	CHO	0.2222	1.1111	9	0.4375
C18H20O4	299.1289	CHO	0.1579	1.2632	8	0.3714
C19H24O3	299.1653	CHO	0.2941	1.0588	9	0.4483
C17H18O5	301.1081	CHO	0.2222	1.2222	8	0.3750
C18H22O4	301.1445	CHO	0.1579	1.3684	7	0.3143
C19H26O3	301.1809	CHO	0.3125	1.0625	9	0.4800
C16H17N1O5	302.1034	CHON	0.3333	1.0667	9	0.5238
C15H16N2O5	303.0986	CHON	0.2105	1.0526	10	0.4706
C19H20O4	311.1289	CHO	0.2105	1.1579	9	0.4118
C19H22O4	313.1445	CHO	0.2941	1.0000	10	0.5185
C17H17N1O5	314.1034	CHON	0.2778	1.1111	9	0.4194
C18H20O5	315.1238	CHO	0.2105	1.2632	8	0.3529
C19H24O4	315.1602	CHO	0.2941	1.1176	9	0.4444
C17H19N1O5	316.119	CHON	0.5385	1.3846	5	0.0588
C13H18O7S1	317.07	CHOS	0.3125	1.1250	9	0.4783
C16H18N2O5	317.1143	CHON	0.5385	1.5385	4	-0.0588
C13H20O7S1	319.0857	CHOS	0.2632	1.0526	10	0.4545
C19H20O5	327.1238	CHO	0.2000	1.2000	9	0.3889
C20H24O4	327.1602	CHO	0.2000	1.3000	8	0.3333
C20H26O4	329.1758	CHO	0.2778	1.1667	9	0.4138
C18H21N1O5	330.1347	CHON	0.5000	1.4286	5	0.0526
C14H20O7S1	331.0857	CHOS	0.2941	1.1765	9	0.4400
C17H20N2O5	331.1299	CHON	0.6154	1.5385	4	-0.1250
C13H20O8S1	335.0806	CHOS	0.3158	0.9474	11	0.5000
C19H18O6	341.1031	CHO	0.2500	1.1000	10	0.4286
C20H22O5	341.1394	CHO	0.1905	1.2381	9	0.3684
C21H26O4	341.1758	CHO	0.2500	1.2000	9	0.3714
C20H24O5	343.1551	CHO	0.2632	1.2105	9	0.3871
C19H23N1O5	344.1503	CHON	0.5714	1.2857	6	0.1111
C14H18O8S1	345.065	CHOS	0.4667	1.4667	5	0.0476
C15H22O7S1	345.1013	CHOS	0.5714	1.4286	5	0.0000
C14H20O8S1	347.0806	CHOS	0.3000	1.0000	11	0.4706
C20H20O6	355.1187	CHO	0.2381	1.1429	10	0.4054
C21H24O5	355.1551	CHO	0.2500	1.1500	10	0.4242
C20H23N1O5	356.1503	CHON	0.2381	1.2381	9	0.3514
C21H26O5	357.1707	CHO	0.3529	1.0000	11	0.5909
C17H17N3O6	358.1045	CHON	0.2500	1.2500	9	0.3636
C20H25N1O5	358.166	CHON	0.5333	1.3333	6	0.1000
C15H20O8S1	359.0806	CHOS	0.4375	1.5000	5	0.0435
C16H24O7S1	359.117	CHOS	0.5333	1.4667	5	0.0000

C15H22O8S1	361.0963	CHOS	0.2857	1.0476	11	0.4444
C21H22O6	369.1344	CHO	0.2273	1.1818	10	0.3846
C22H26O5	369.1707	CHO	0.2273	1.2727	9	0.3333
C22H28O5	371.1864	CHO	0.3333	1.0556	11	0.5417
C18H19N3O6	372.1201	CHON	0.6000	1.3333	6	0.0526
C15H20O9S1	375.0755	CHOS	0.5000	1.5000	5	0.0000
C16H24O8S1	375.1119	CHOS	0.2174	1.3043	9	0.3171
C23H30O5	385.202	CHO	0.3158	1.1053	11	0.5000
C19H21N3O6	386.1358	CHON	0.4706	1.4118	6	0.0833
C17H24O8S1	387.1119	CHOS	0.3000	1.0500	12	0.5357
C20H21N3O6	398.1358	CHON	0.2083	1.3333	9	0.3023
C24H32O5	399.2177	CHO	0.3000	1.1500	11	0.4643
C20H23N3O6	400.1514	CHON	0.4444	1.4444	6	0.0769
C18H26O8S1	401.1276	CHOS	0.5294	1.4118	6	0.0435
C17H24O9S1	403.1068	CHOS	0.3500	0.9500	13	0.5926
C20H19N3O7	412.115	CHON	0.2857	1.0952	12	0.5000
C21H23N3O6	412.1514	CHON	0.2857	1.1905	11	0.4333
C21H25N3O6	414.1671	CHON	0.4211	1.4737	6	0.0714
C19H28O8S1	415.1432	CHOS	0.5882	1.2941	7	0.0909
C17H22O10S1	417.0861	CHOS	0.5000	1.4444	6	0.0400
C18H26O9S1	417.1225	CHOS	0.5882	1.4118	6	0.0000
C17H24O10S1	419.1017	CHOS	0.4737	1.4737	6	0.0370
C19H28O9S1	431.1381	CHOS	0.5556	1.4444	6	0.0000
C18H26O10S1	433.1174	CHOS	0.5263	1.3684	7	0.0769
C19H26O10S1	445.1174	CHOS	0.4500	1.5000	6	0.0345
C20H30O9S1	445.1538	CHOS	0.5263	1.4737	6	0.0000
C19H28O10S1	447.133	CHOS	0.5789	1.2632	8	0.1200
C19H24O11S1	459.0967	CHOS	0.5000	1.4000	7	0.0714
C20H28O10S1	459.133	CHOS	0.5789	1.3684	7	0.0400
C19H26O11S1	461.1123	CHOS	0.5000	1.5000	6	0.0000
C20H30O10S1	461.1487	CHOS	0.5789	1.4737	6	-0.0400
C19H28O11S1	463.128	CHOS	0.5500	1.3000	8	0.1111
C20H26O11S1	473.1123	CHOS	0.4762	1.4286	7	0.0667
C21H30O10S1	473.1487	CHOS	0.4545	1.4545	7	0.0625
C22H32O10S1	487.1643	CHOS	0.5238	1.4286	7	0.0345
C21H30O11S1	489.1436	CHOS	0.3571	1.7143	3	0.0435
C14H24O5	271.1551	CHO	0.4286	1.5714	4	0.0909
C14H22O6	285.1344	CHO	0.4000	1.6000	4	0.0833
C15H24O6	299.15	CHO	0.5000	1.5714	4	0.0476
C14H22O7	301.1293	CHO	0.4667	1.6000	4	0.0435
C15H24O7	315.1449	CHO	0.5000	1.5714	4	-0.0526
C14H22O7S1	333.1013	CHOS	0.4667	1.6000	4	-0.0476
C15H24O7S1	347.117	CHOS	0.5714	1.5714	4	-0.1111
C14H22O8S1	349.0963	CHOS	0.5000	1.6250	4	-0.0909
C16H26O8S1	377.1276	CHOS	0.3333	1.6190	5	0.0857
C21H34O7	397.2232	CHO	0.4444	1.5556	5	0.0000
C18H28O8S1	403.1432	CHOS	0.5000	1.5556	5	-0.0400

Table S4 Medium for the incubation of *Comamonas testosteroni* ATCC11996

Chemicals	Concentration (mg/L)
$CaCl_2·2H_20$	10
$MgSO_4·7H_2O$	500
NH_4Cl	10.698
K_2HPO_4	557.376
KH_2PO_4	108.872
Testosterone	144.21
$FeSO_4·7H_20$	5.2
Na_2-EDTA	5.2
H_3BO_3	0.03
$MnCl_3·4H_2O$	0.1
$CoCl_2·6H_2O$	0.19
$NiCl_2·6H_2O$	0.024
$CuCl_2·2H_2O$	0.029
$ZnSO_4·7H_2O$	0.144
$NaMoO_4·7H_2O$	0.036

Supplementary references

1. Lian J, Zheng X, Zhuo X, Chen Y-L, He C, Zheng Q, Lin T-H, Sun J, Guo W, Shi Q, Jiao N, Cai R. 2021. Microbial transformation of distinct exogenous substrates into analogous composition of recalcitrant dissolved organic matter. Environmental Microbiology 23:2389-2403.
2. Chiang YR, Wei STS, Wang PH, Wu PH, Yu CP. 2020. Microbial degradation of steroid sex hormones: implications for environmental and ecological studies. Microbial Biotechnology 13:926-949.
